# Facial expressions of Asian people exposed to constructed urban forests: Accuracy validation and variation assessment

**DOI:** 10.1371/journal.pone.0253141

**Published:** 2021-06-17

**Authors:** Haoming Guan, Hongxu Wei, Richard J. Hauer, Ping Liu

**Affiliations:** 1 School of Geographical Sciences, Northeast Normal University, Changchun, China; 2 Key Laboratory of Wetland Ecology and Environment, Northeast Institute of Geography and Agroecology, Chinse Academy of Sciences, Changchun, China; 3 University of Chinese Academy of Sciences, Beijing, China; 4 College of Natural Resources, University of Wisconsin-Stevens Point, Stevens Point, Wisconsin, United States of America; 5 College of Forestry, Shenyang Agricultural University, Shenyang, China; National Institutes of Health, UNITED STATES

## Abstract

An outcome of building sustainable urban forests is that people’s well-being is improved when they are exposed to trees. Facial expressions directly represents one’s inner emotions, and can be used to assess real-time perception. The emergence and change in the facial expressions of forest visitors are an implicit process. As such, the reserved character of Asians requires an instrument rating to accurately recognize expressions. In this study, a dataset was established with 2,886 randomly photographed faces from visitors at a constructed urban forest park and at a promenade during summertime in Shenyang City, Northeast China. Six experts were invited to choose 160 photos in total with 20 images representing one of eight typical expressions: angry, contempt, disgusted, happy, neutral, sad, scared, and surprised. The FireFACE ver. 3.0 software was used to test hit-ratio validation as an accuracy measurement (ac.) to match machine-recognized photos with those identified by experts. According to the Kruskal-Wallis test on the difference from averaged scores in 20 recently published papers, contempt (ac. = 0.40%, *P* = 0.0038) and scared (ac. = 25.23%, *P* = 0.0018) expressions do not pass the validation test. Both happy and sad expression scores were higher in forests than in promenades, but there were no difference in net positive response (happy minus sad) between locations. Men had a higher happy score but lower disgusted score in forests than in promenades. Men also had a higher angry score in forests. We conclude that FireFACE can be used for analyzing facial expressions in Asian people within urban forests. Women are encouraged to visit urban forests rather than promenades to elicit more positive emotions.

## Introduction

The purpose of planting, growing, and managing urban tree populations is to provide the ecological services of urban forests and to promote human well-being in urban green spaces [1]. The principle of the construction of sustainable urban forests is that a community should promote the social and ecological needs of people [2, 3]. An urban forest experience can elicit more positive emotional responses of pedestrians compared to a promenade experience [4, 5]. The efficiency of said emotional response, however, may not always be at a level that improves mental health [6, 7].

Emotion is a transitory mental response to an external stimuli [8]. Emotional response to forests can be assessed using questionnaire methodology [9]. Pilot studies in this context mainly argues that a forest experience can counter negative emotions by alleviating anxiety and stress [10–12]. However, one vital issue in these studies is that there is an absence of systematic validation on the use of questionnaires for forest visitors [13]. It is uncertain whether facial expressions were related to internal emotions and the extent to which emotions matched expression scores. Validation can solve this issue.

Emotions can be categorized as either a felt expression with an emotional cue (Duchenne) or an unfelt expression with a communicative cue (non-Duchenne) [14]. Both kinds of facial expressions can be assessed using facial expression recognition techniques [8]. For over five decades, artificial recognition of facial expressions was believed to be a reliable evaluation of emotions [15]. Automatic recognition instruments outperform traditional approaches in efficiency and accuracy [16, 17]. However, validation of facial expression recognition instruments is needed to understand the level of accuracy and precision of this recognition. It is uncertain whether facial expressions were related to internal emotions and the extent to which emotions matched expression scores. Validation can solve this issue.

The validation of face reading is a test that quantifies the percentage of agreement between artificially matching the aimed emotion [15, 18] and automatic machine reading [16, 17, 19, 20]. Early methods to validate the accuracy of recognizing emotional expressions used “matching scores” that were rated by observers regarding photos with intended expressions [15]. This was termed as “the percentage of observers who choose the predicted label” [21]. Early methods were further developed by instruments trained using datasets of expressions. Currently, notable software for facial expression recognition include Facial Action Coding System (FACS) [16, 19], FaceReader [16, 19], Affectiva Media Analytics [20], Face Analysis Computer Expression Toolbox (FACET), and iMotion [17, 20]. Datasets of facial expressions frequently used to train machine learning mostly come from the Warsaw Set of Emotional Facial Expression Pictures and the Amsterdam Dynamic Facial Expression Set [19]. The arithmetic coding for the movement of facial muscles originated from prototypical expressions on western-race faces. However, emotional expressions are not universal [22]. To more accurately detect Asian facial expressions, software that is trained by datasets of facial expressions of Asian volunteers is used [23–25]. Validation can increase the accuracy of facial expression recognition by excluding inaccurate responses. The precision of matching facial expressions to internal emotions can be increased when photos were recognized and rated by Asian volunteers.

FireFACE (Zhilunpudao Agric. S&T Inc., Changchun, China) is a software that was produced to analyze the facial expressions of Asian people [23]. The basic arithmetic was established through machine-training using 30,000 photos of facial expressions from Asian people. Version (ver.) 1.0 documented three expressions (happy, sad, and neutral) that were classified by experts [23, 25], and ver. 2.0 documented five expressions (happy, sad, neutral, angry, and scared). Ver. 3.0 was analyzed eight basic expressions (happy, sad, neutral, angry, scared, surprised, disgusted, and contempt) using the initial dataset and a subsequent dataset of photos of Asian urban forest visitors from across mainland China [24, 26]. FireFACE ver. 1.0 has been successfully used for assessing the variation of emotional expressions in university campuses [23] and at an urban forest park [25]. FireFACE ver. 3.0 has been used to detect the combined effects of geographical variation across urbanization gradient on facial expressions of urban forest visitors in Northeast China [24]. FireFACE has shown the desired precision in facial analysis for people in urban forests, although most expressions were subtle. It is necessary to further increase matching accuracy by validating facial expression scores.

The change in setting along the urbanization gradient serves as cues for people to respond with varied emotional expressions [27, 28]. The perception to infrastructure and openness are determinants of emotional variation at different places [23, 28]. When urban forests are considered an objective infrastructure, people will show particular expressions different from those in city settings [24, 25]. In this study, FireFACE ver. 3.0 was used to test the difference in the emotional perceptions between people in constructed urban forests and people in promenades. Only facial expression that passed the validation test were used for geographical comparison. Based on the current success of subtle expressions analysis using FireFACE, we hypothesized that: (i) at least five out of the eight matching scores can meet the validation accuracy of a commercial software, and (ii) people in urban forests will show a significant difference in emotions, not only in basic expressions (happy, sad, and neutral) but also in implicit ones (angry, surprised, scared, disgusted, and contempt).

## Materials and methods

### Field data collection

Field data were collected from an urban forest park and an promenade in Shenyang City (41°11’–42°17’ N, 122°21’–123°48’ E). Shenyang is located in the transitional belt between Changbai Mountains and the alluvial plain of Liaohe River. Shenyang had 8.3 million permanent residents distributed across an area of 6.3 million km^2^ built-up region in 2018. Shenyang is located in a semi-humid, temperate continental climate zone with annual average temperatures of 6.2–9.7 °C and a range of -32.9°C and 38.4°C. Annual rainfall in Shenyang ranged between 600 and 800 mm with a historical maximum precipitation of 716.2 mm. Yearly frost-free periods lasted for 155–180 d. Climatic data spanned from 1951 to 2018 [29].

**Fig 1:**
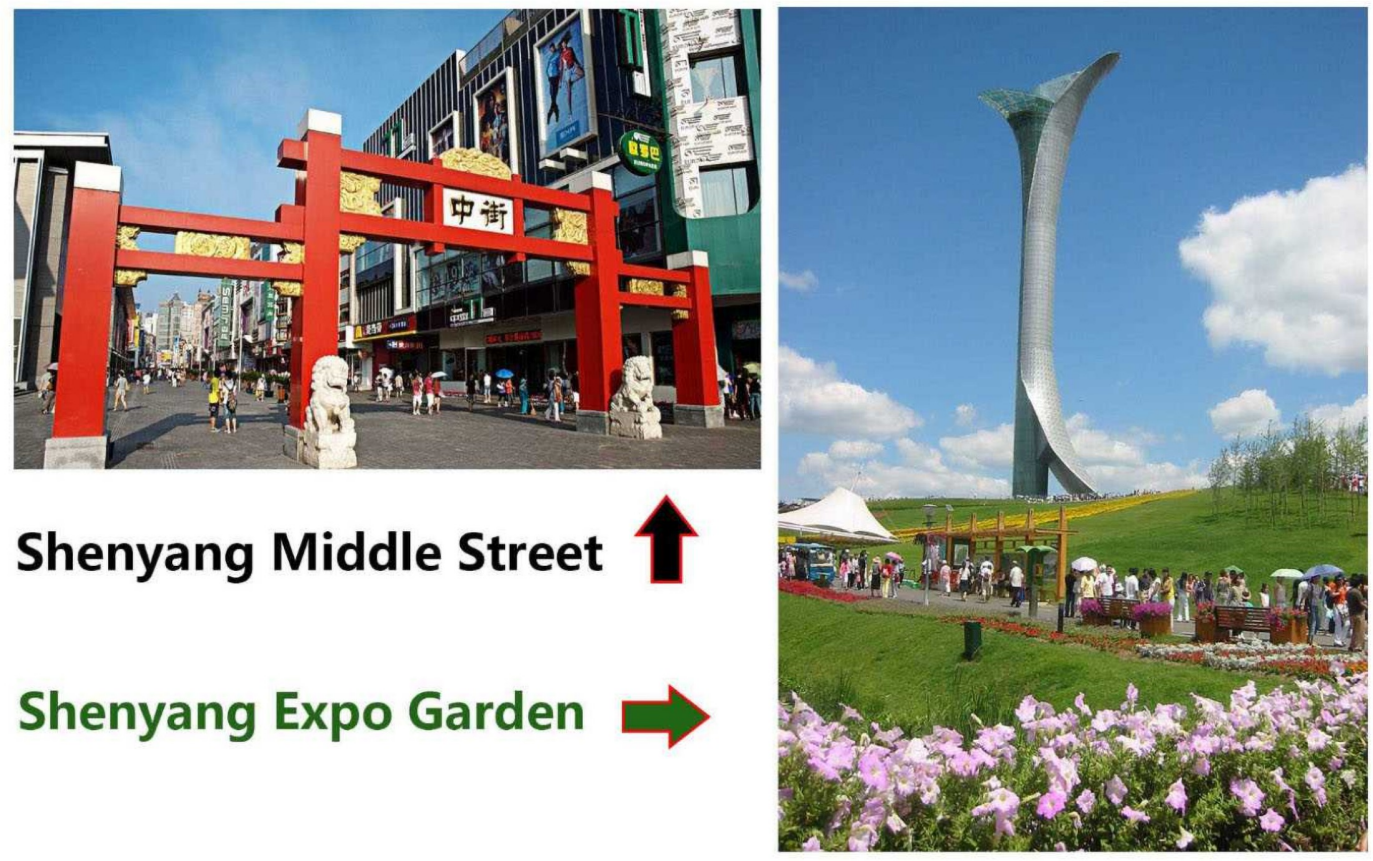
Shenyang Expo Garden (forest) and Shenyang Middle Street (Promenade or Urban) in Shenyang City, Northeast China.

Shenyang Expo Garden (SEG) (41°49’ N, 123°37’ E) was chosen as the site of urban forests and Shenyang Middle Street (SMS) (41°48’ N, 123°25’ E) as the promenade (Fig 1). SEG was established in February of 1959 with an openness area of 211 ha with 196 ha of green lands and 6.5 ha of watershed. Urban forests in SEG were constructed since 1988. The daily number of visitors in SEG ranged between 0.3 million and 0.7 million which is the highest record for all green spaces around the plains of Liaohe River. SMS has a length of 579.3 m and a width of 11.7 m, the longest promenade in mainland China. SMS has had a long history of use from 1625 up to now. SMS is rarely greened along the sidewalk and has areas fully occupied by groceries, markets, and plazas, which attracts anywhere from 0.4 to 2 million daily visitors. Therefore, SEG and SMS are two typical infrastructures with contrasting green spaces and constructed landscapes.

### Participants

Eight students from College of Forestry, Shenyang Agricultural University were recruited as data collectors in this study. They were assembled as a group of volunteers on 19 June, 2020. All had been informed about the aim, process, and possible obstacles of the study. Only those who agreed to all the details of the study were recruited. The constant of participants has been documented as the S1 Raw data where participants provided written informed consent. Candidates with smoking or alcohol consumption habits were excluded in the recruitment. Eight students were randomly assigned to two groups with four in each group. One group investigated SEG and the other investigated SMS the first day. In the following day, places were exchanged. Two students in one group took photos and the other two asked participants for the consent of using photos for scientific work. Photographers used a camera of imx-586 (Sony NEC Optiarc Inc., Tokyo, Japan) with 4 million px which was embedded in the cellphone.

The Ethic Committee of the Research Group of Urban Forests and Wetlands, Northeast Institute of Geography and Agroecology, Chinese Academy of Sciences, provided approval for your study. On weekends of the 20^th^ and 21^st^ of June, 2020, all visitors with typical Asian faces were photographed at SEG and SMS. All visitors whose faces have been photoed and recorded for this study had been informed about the aim of the study and provided their oral consents. This procedure was approved by the Ethics committee. Participants for photo collection supplied written informed consent. Visitors with faces characteristic of Chinese populations were subjects to be photographed [30]. Faces were easily identified through subjective recognition by students, but it was hard to distinguish between those of Chinese ethnicity and those of other East Asian countries. Therefore, we extended the standard to Asians in general. Both days were sunny and cloudless except for June 21^st^ from 12:00 to 14:00. The temperature ranged between 21°C and 32 °C in daytime with southwesterly winds at a velocity of Beaufort force 4 (24 km/h average speed). Photos in both sites were taken from 09:00 am to 05:00 pm (GMT+8) in accordance with the opening time of SEG. The route in SEG started at the entrance and ended at the exit with 4 repeated cycles of data collection along the sidewalks, while the route in SMS started at the northern entrance along the western side of the sidewalk in the morning and the eastern side in the afternoon to avoid building shadows.

### Available photos with facial expressions

All photos that fulfilled the standard for further analysis needed to connect at least one visitor’s face with the five facial organs—eyebrows, eyes, nose, mouth, and ears—no matter which angle the face was photographed from. Photos were labeled as potential candidates when only one ear can be seen, with the rest of the facial organs visible. All photos were cropped so that the subject’s face is in the center and all organs are clearly exposed. A singular photo with all attributes is the best for facial expression analysis, but multiple photos with some attributes can be pieced together. A total of 2,886 photos met the criteria for further analysis. The approval for ethic statement has been documented as the S1 Raw data.

### Validation of matching accuracy

A dataset of facial photos was generated from all documented photos for validation. Groups of 20 photos were selected from the pool of both SEG and SMS with each group demonstrating a particular emotion: angry, contempt, disgusted, happy, neutral, sad, scared, and surprised. A total of 160 photos were reviewed by six experts in the domain of urban ecology from four affiliations. The constant of experts for the dataset review has been documented to the S1 Raw data. The final edition of the dataset was revised according to suggestions from all experts and selections received unanimous agreement.

Validation was determined by the ‘matching accuracy’ variable, which is the matching percentage of the number of photos that were correctly recognized by the instrument for the predicted emotional expressions of prototypical faces for each of the 20 images [31–33]. Therefore, matching accuracy can be regarded as the percentage for correct matching. It is possible that facial photos may contain multiple expressions in different emerging values. Only the expression with highest value was considered for matching [20].

**Fig 2:**
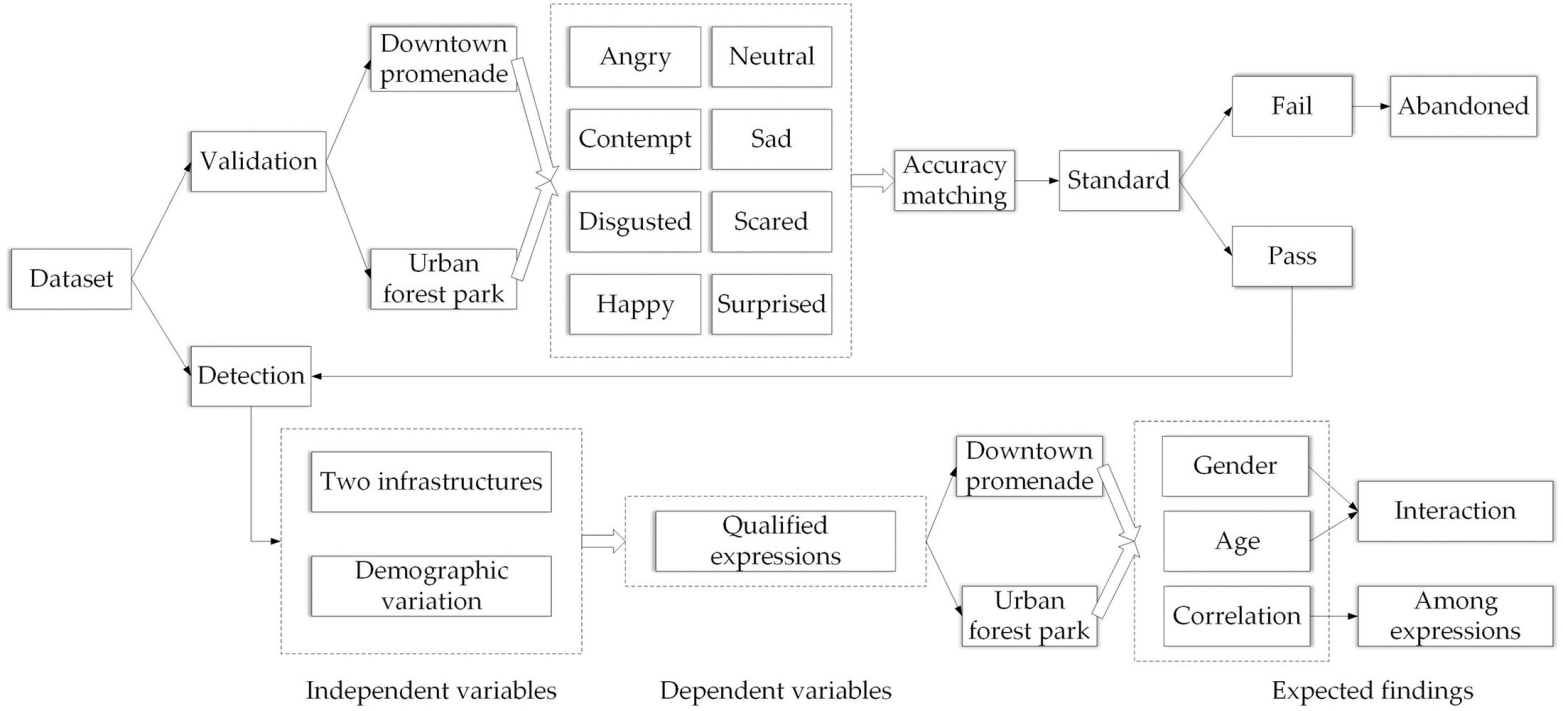
The whole process of the layout of whole study from validation to analysis.

Give that the matching accuracy of validation for facial expressions varied widly depending on the choice of database, methodology, and instrument, we established a set of standards to screen for the validation of each of the eight expressions in our photos from SEG or SMS. A combination of the keywords of ‘validation’ + ‘accuracy’ + ‘facial expression’ was checked in the search engine of Web of Science (Clarivate Analytics, Philadelphia, Pennsylvania, USA). The 20 most relevant studies with specific sources of data (either from figure, words, or tables) were documented for data extraction. The criteria when screening for usage was adapted as the mean of 20 studies (Table 1) [16, 30–48]. The specific process is shown in Fig 2. Only expressions that passed the criteria were used for further assessment.

**Table 1:** Summary of matching accuracy for validation studies on facial expressions.

Source	Instrument	Data source	Matching accuracy (%)							
			Ang.^1^	Con.^2^	Dis.^3^	Hap.^4^	Neu.^5^	Sad	Sca.^6^	Sur.^7^
Banziger et al. [34]	Artificial recognition	Figure	27	-	85	56	-	47	-	-
Ebner et al. [36]	Artificial recognition	Figure	80	-	69	-	84	73	84	-
Besel and Yuille [35]	Artificial recognition	Table	82	-	55	69	75	58	54	51
Matuszewski et al. [37]	Eigenface, Fisherface	Table	62	-	55	83	-	51	35	62
Huang et al. [30]	E-Prime	Figure	54	-	32	63	43	33	27	21
Maniglio et al. [38]	Artificial recognition	Table	50	-	-	93	65	75	65	-
Zhang et al. [40]	Artificial recognition	Table	43	-	-	69	-	43	-	-
Olszanowski et al. [39]	Artificial recognition	Figure	95	-	100	78	57	95	78	76
Wingenbach et al. [41]	E-Prime	Figure	74	35	65	84	89	78	62	92
Vaiman et al. [43]	FACS	Table	85	-	80	95	87	78	63	91
Kim et al. [42]	FACS	Figure	87	-	63	97	93	84	50	93
Saeed et al. [46]	AERS ^8^, GSNMF ^9^, TPTSR ^10^	Figure	60	-	52	54	-	57	52	50
Mishra et al. [44]	E-Prime	Figure	80	-	79	96	88	86	80	88
Prada et al. [45]	Artificial recognition	Table	79	-	69	89	75	70	58	80
Chung et al. [47]	Artificial recognition	Figure	90	-	60	99	73	83	36	92
Verpaalen et al. [48]	Artificial recognition	Figure	59	34	53	93	63	70	47	66
Skiendziel et al. [16]	FaceReader Ver. 7	Words	76	-	92	96	94	86	82	94
Yang et al. [33]	Amazon Rekognition, Baidu Research, Face++, Microsoft Azure, Affectiva	Table	64	-	91	99	98	85	56	97
Krumhuber et al. [32]	FACET	Figure	48	-	57	88	-	54	34	46
Bijsterbosch et al. [31]	Artificial recognition	Table	77	39	68	92	81	75	63	64
Mean			68.60	36.00	68.06	83.53	77.67	69.05	57.00	72.69
Standard deviation			17.41	2.16	16.70	14.05	14.90	16.51	16.71	21.57

Note:

^1^ Ang., angry;

^2^ Con., contempt;

^3^ Dis., disgusted;

^4^ Hap., happy;

^5^ Neu., neutral;

^6^ Sca., scared;

^7^ Sur., surprised;

^8^ AERS, automatic expression recognition system;

^9^ GSNMF, graph-preserving sparse nonnegative matrix factorization;

^10^ TPTSR, two-phase test sample representation.

### Assessment of variation and statistics

The eight students were invited again to create demographic categories. All photos were classified by gender (man vs woman) and age (senior [over 60 years-old], middle-aged [35–50 years-old], youth [15–25 years-old], toddler [0–5 years-old]). We categorized gender according to visually identifiable, biological characteristics. Age was categorized by empirical identification to visual standards of the median of each age group. Some age categorizations were ambiguous for identification between senior and middle ages or between young and toddler ages. All eight students assembled together, discussed, and voted for the final choice.

Each of the validated expressions, the dependent variable, was analyzed in response to the combined independent variables of gender (*n* = 2), age (*n* = 4), and location (*n* = 2). Only facial expressions that passed the validation with no difference from the average in other literature was used for next-step analysis.

Data were analyzed using SAS software (STS Institute, Cary, NC, USA). A new parameter, termed positive response index (PRI) [23–25], was employed to evaluate the net difference between happy and sad expression scores. In validation, the Kruskal-Wallis test was repeatedly used to detect the difference between the critical standard for expressions from literature (*n* = 20) and expressions from our database. The basic probability of significance was taken at the 0.05 level as adjusted by the Bonferroni method to 0.00625 due to eight repeated comparisons. Thus, scores for contempt, neutral, scared, and surprised expressions were not recorded in the 20 documented studies for validation (Table 1). Only the expressions that did not show significant difference between our database and previous studies were used as parameters for further assessment. In variation assessment, all data were ranked to avoid abnormally distributed data that invalidated use of a general linear model. Every expression was tested for response to three-way analysis of variance (ANOVA) across gender, age, and location. When significant effect was found, data were ranked and compared by a one-way ANOVA with all combined factors together as the single source of variance (*α* = 0.05). The principle component analysis (PCA) was used to bridge the grouped tendency of correlation that had been used in several former studies [49, 50].

## Results

### Validation of recognition accuracy

As shown in Table 1, the selected 20 publications reporting facial expression accuracy did not supply data for all eight expressions. For example, only three out of the 20 publications evaluated accuracy for contempt expression and 15 out of 20 for neutral expression. The highest accuracy was found for happy expression scores, followed by neutral and surprised expressions. The lowest accuracy was found for contempt expression scores, and the rest were all above 50%.

Results on FireFACE’s accuracy in recognizing the eight facial expressions are shown in Table 2. The accuracy in recognizing contempt and scared expressions was significantly lower than the average from the 20 publications. Although averaged accuracy, when comparing our database to historical ones, was lower by 18–69% for the rest of the facial expressions, repeated Kruskal-Wallis tests did not indicate any significant difference because raw data showed a large variation in both our and previous databases. Therefore, we accept FireFACE’s accuracy in recognizing facial expressions, but only for the six expressions of anger, disgust, happiness, sadness, neutral, and surprise.

**Table 2:** Accuracy of eight facial expressions using FireFACE software and repeated Kruskal-Wallis tests on the difference from records in documented studies that are shown in Table 1.

Facial expressions	DF	Accuracy (%)	SD ^1^	Chi-Square	Pr > Chi-Square ^2^
Angry	1	56.11	13.81	5.5388	0.0186
Contempt	1	0.40	0.90	8.3623	0.0038*
Disgusted	1	43.98	22.72	6.0610	0.0138
Happy	1	60.55	35.24	1.0296	0.3102
Neutral	1	23.85	20.89	7.3023	0.0069
Sad	1	55.53	11.69	7.1742	0.0074
Scared	1	25.23	20.57	9.6959	0.0018*
Surprised	1	47.57	15.75	2.8156	0.0934

Note:

^1^ SD, standard deviation;

^2^ asterisk indicates significant difference at the 0.00625 level.

### Analysis of variance on facial expressions in Shenyang

As shown in Table 3, excluding scores for anger and PRI, scores for the rest of the facial expressions demonstrated significant responses to variation between forest and urban locations. All facial expression scores, except scores for surprise, were different by gender. Happy expression scores did not vary by visitor age, but responded significantly to the interaction between location and age. Happy expression scores also responded to the interaction between gender and age. In addition, angry, surprised, and disgusted expression scores showed a significant response to the interaction between location and gender as well.

**Table 3:** *P* values from analysis of variance (ANOVA) of location (L), gender (G), age (A), and their interactions on ranked scores of neutral, happy, sad, angry, surprised, and disgusted expressions.

Source	DF ^1^	Neutral	Happy	Sad	PRI ^2^	Angry	Surprised	Disgusted
L	1	**< .0001** ^3^	**< .0001**	**0.0002**	0.5283	0.0978	**< .0001**	**0.0005**
G	1	**0.0046**	**0.0022**	**< .0001**	**0.0002**	**0.0455**	0.6032	**< .0001**
A	9	**< .0001**	0.0704	**< .0001**	**< .0001**	**0.0001**	**< .0001**	**< .0001**
L×G	1	0.2388	0.8089	0.1089	0.1537	**0.0082**	**0.0406**	**0.0151**
L×A	3	0.7786	**0.0083**	0.0771	0.2625	0.5605	0.5979	0.4644
G×A	3	0.1385	**0.0467**	0.6569	0.9874	0.6179	0.5579	0.9863
L×G×A	3	0.8708	0.5846	0.2112	0.1217	0.1611	0.2955	0.2107

Note:

^1^ DF, degree of freedom;

^2^ PRI, positive response index;

^3^ Bold font highlights significant effect.

### Response of happy expression scores

**Fig 3:**
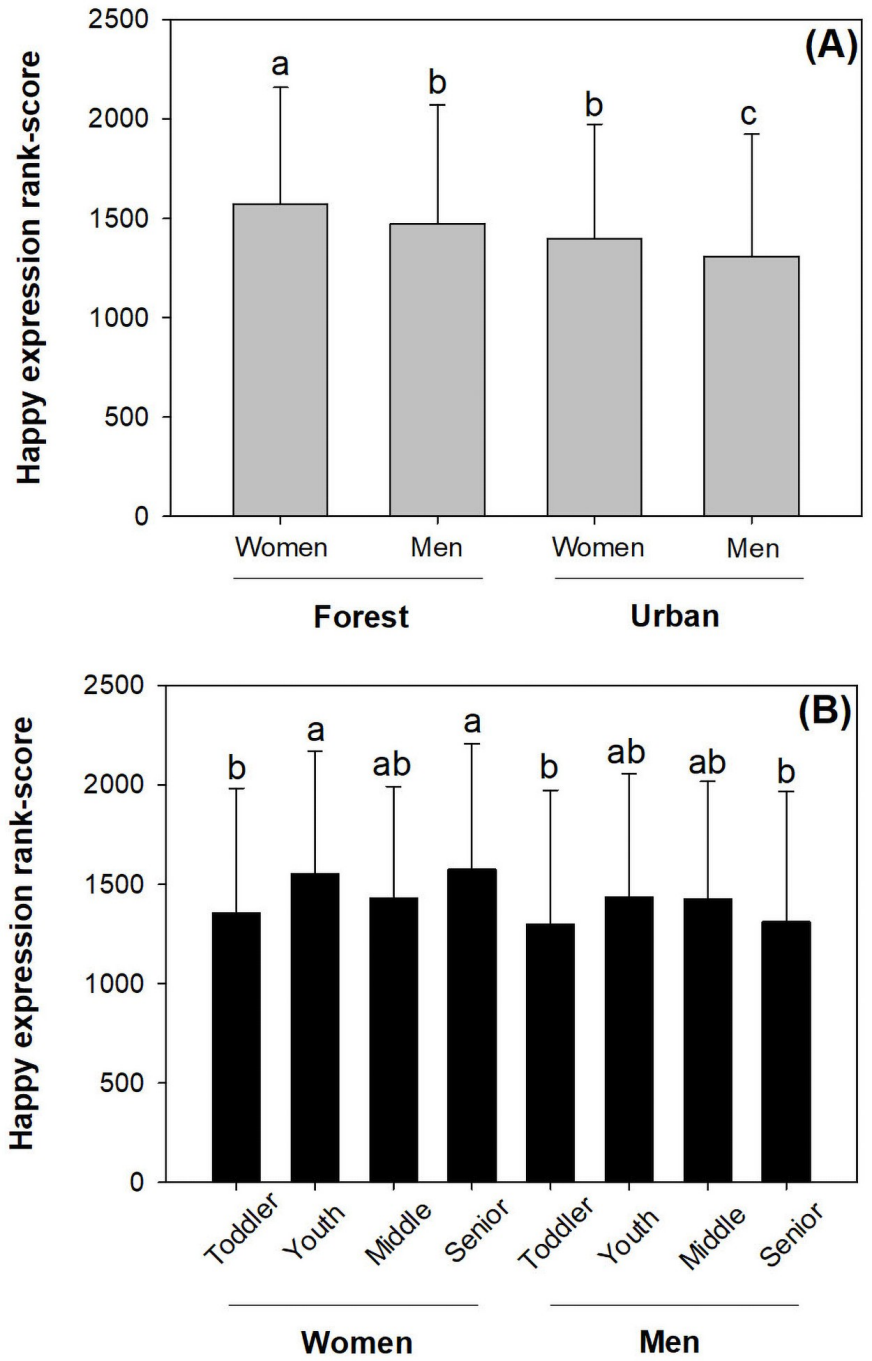
Happy expression scores for men and women at different ages in forest and urban locations. Scores have been transformed by ranking. Bars stand for standard errors. Different letters indicate significant difference according to Duncan test at 0.05 level.

Women had higher happy expression scores than men in both urban forest and promenade (Fig 3A). Women had the highest happy expression score in the forest locations. Youths and senior women had higher happy expression scores than toddlers and senior men (Fig 3B).

Forest visitors had higher happy expression scores than those in promenades by 13%. Women had higher happy expression scores than men by 8%. Young visitors had higher happy expression scores than toddlers by 12%. The happy expression scores of youths were not different from middle-aged or old-aged visitors.

### Responses of angry, surprised, and disgusted expression scores

**Fig 4:**
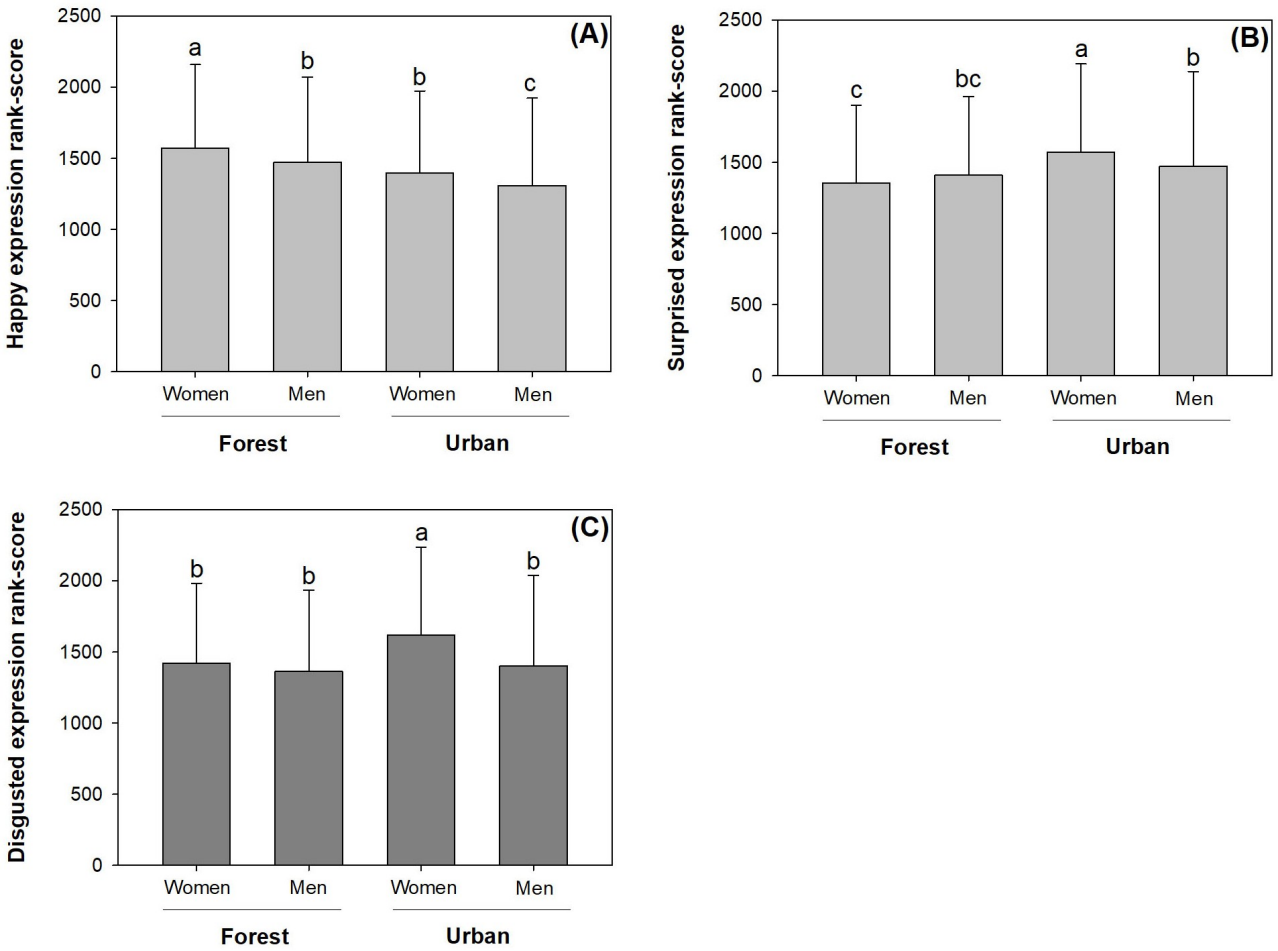
Expression scores for angry, surprised, and disgusted emotions in men and women in forest and urban locations. Scores have been transformed by ranking. Bars stand for standard errors. Different letters indicate significant difference according to Duncan test at 0.05 level.

Men in forest areas had the highest angry expression score in response to interaction between gender and location (Fig 4A). In contrast, women in promenades had the highest surprised and disgusted expression scores (Fig 4B and 4C). Although the surprised expression scores of men in promenades were lower than women, men’s scores were higher than those of women in forest areas (Fig 4B).

There were no distinct angry expression scores between visitor in different locations (Table 3). Women generally had a higher angry expression score than men by 4%. Middle-aged visitors had higher angry scores than toddlers and senior citizens by 22% and 11%, respectively.

Both surprised and disgusted expression scores were higher in promenade than in forests. Both expression scores were also higher for youths than for toddlers and senior citizens. Women had higher disgusted expression scores than men by 9%.

### Responses of neutral and sad expression scores

**Fig 5:**
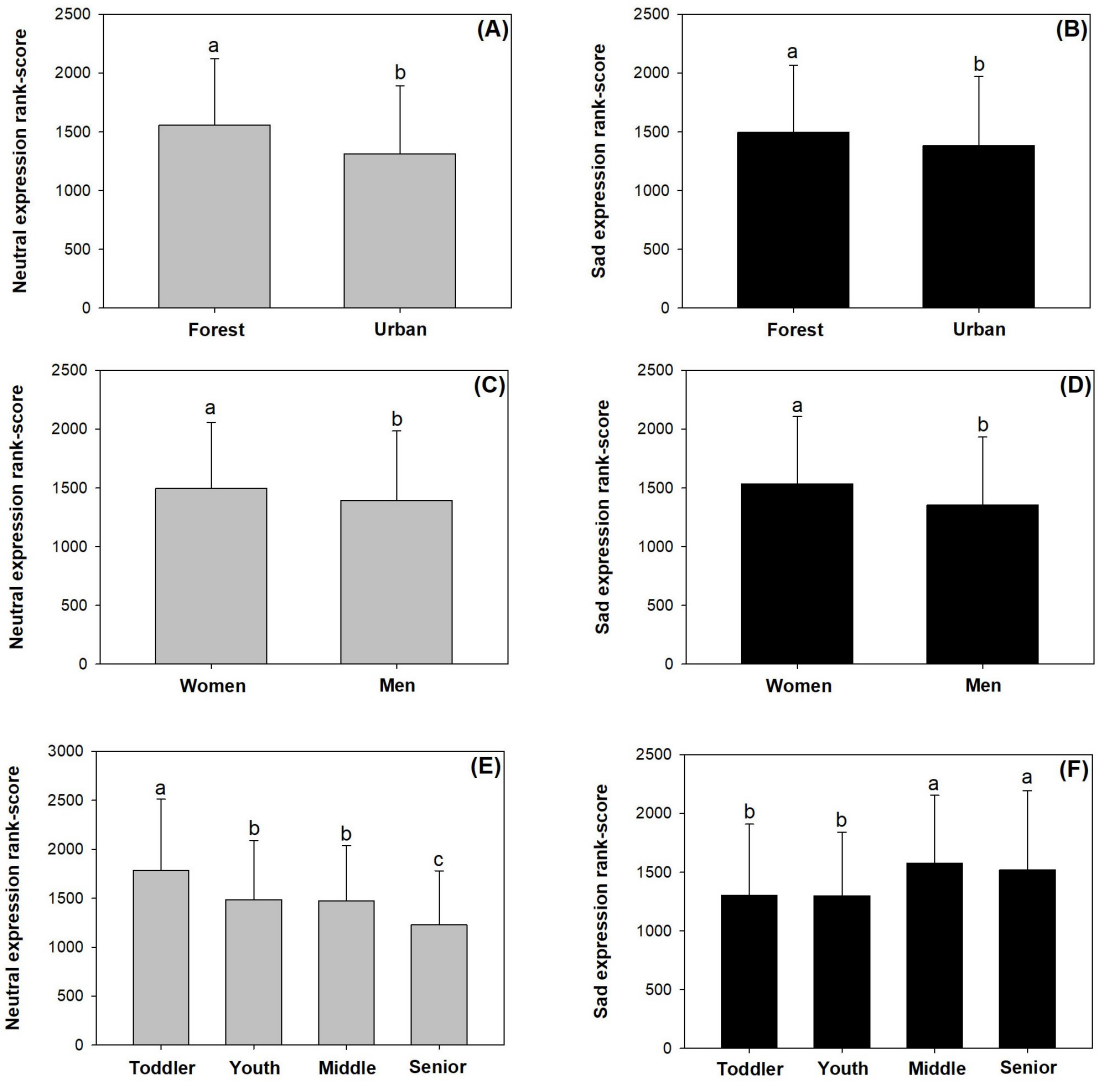
Neutral and sad scores with variations by location (forest vs urban), gender (men vs women), and age (toddler, youth, middle, senior). Scores have been transformed by ranking. Bars stand for standard errors. Different letters indicate significant difference according to Duncan test at 0.05 level.

Both neutral and sad expression scores were higher in the urban forests than in cities (Fig 5A and 5B). Women had higher neutral and sad expression scores than men (Fig 5C and 5D). The neutral expression scores generally decreased as age increased, but older visitors had higher sad expression scores than toddlers and young visitors (Fig 5E and 5F).

### Response of PRI

PRI did not show any significant response to the difference between forest and urban locations (Table 3). Men had higher PRI than women by 8%. Toddlers and youths had higher PRI than older visitors.

### PCA analysis

**Fig 6:**
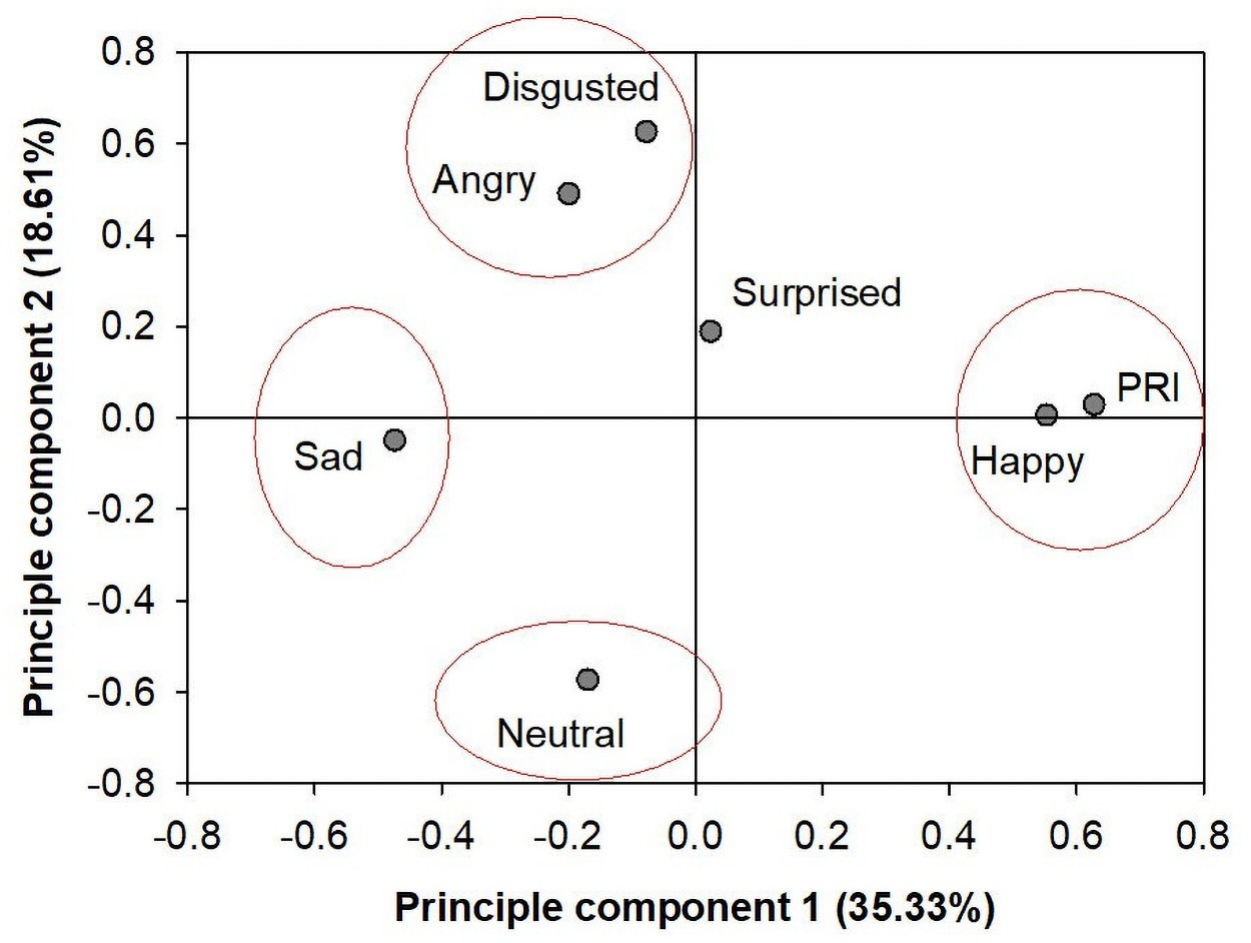
Eigenvalues of happy, sad, neutral, angry, disgusted, and surprised expression scores and positive response index (PRI) in the first two principle components.

The data pool of the six facial expressions and PRI combined indicate a variation in which the first PC explains 35.3% and the first two PC cumulatively explains 53.93%. Therefore, an explanation above 50% supports a further analysis on the synthesis of the first two PCs. The sad expression score had an inverse relationship with the happy expression score and PRI (Fig 6). Both the disgusted and angry expression scores had an inverse relationship with the neutral expression score, but the relationship was weaker for anger than for disgust. The surprised expression score did not show any obvious relationship with any other emotional expressions.

## Discussion

### Validation of recognizing accuracy

In our study, contempt and scared expression scores evaluated by FireFACE failed to pass the validation test. The scared expression was also recognized at a low accuracy for the faces of Chinese people even when using the three-dimensional paradigm technique [30]. The contempt emotion is difficult to detect, not only with FireFACE but also with other instruments with a wider range of users. This is further corroborated by the fact that only three out of the recent 20 relevant publications had accuracy in recognizing the contempt expression. The matching accuracy in these three cases were around 30%, which was much lower than the recognition of other expressions. In addition, two cases of accuracies were achieved through artificial rating [31, 48], and only one case published a matching accuracy that was given by an instrument, E-Prime [41]. Therefore, further improvement is needed for FireFACE to recognize the contempt expression because scores were too low for accurate determination. Machine learning techniques need improvement to recognize the exhibition of the contempt emotion in different groups of people.

Chinese people, characteristic of how Asians typically display emotions, show emotions implicitly rather than explicitly. For example, Chinese people’s expression of fear forms more slowly than other negative expressions when depicting pain, even after sad emotions priming [51]. Chinese people’s tendency to suppress expressions of fear by adopting a self-reserved character can be extended to the Korean population’s Yonsei database [47]. Krumhuber et al. [32] compared human and machine (FACET software) validations across 14 datasets of dynamic facial expressions and only obtained a 34% accuracy in recognizing a scared expression. They further found that the scared expression was easily confused with the surprised expression. Matuszewski et al. [37] checked a dataset of facial expressions from 80 clinic patients and, again, found a low recognition of the scared expression and corroborated the easy confusion with the surprised expression. Matuszewski et al. [37] further compared different levels of the scared scores and indicated that patients directly expressed fear only when exposed to extreme pain. Otherwise, they would choose to reserve their expressions to avoid perception by others. Overall, more precisely distinguishing between scared and surprise expressions is suggested to increase the accuracy of recognition.

Our matching accuracy was generally lower than those found in previous studies. This can be explained with two reasons. First, our dataset that was used to train FireFACE contained subjective errors when artificially documenting different facial photos into any of the eight types of expressions. Second, our objects receiving the tests were collected by randomly photographing visitors and subjectively labeling the type of facial expressions, hence, the precision was limited in addition to subjective error. In contrast, both machine-training and objects-testing in the 20 studies reviewed in this paper employed models with instructions to exhibit aimed expressions. Even so, our results on the matching accuracies for the six facial expressions of anger, disgust, happiness, sadness, neutral, and surprise were not statistically different from current ones. Therefore, FireFACE recognition for Asian facial expressions has an acceptable accuracy for six expressions. Thus, we can accept our first hypothesis.

### Facial expression in constructed forests and promenade

It is unexpected that both happy and sad expressions were higher for visitors in constructed forests than in urban promenades. However, there was no difference in net positive score between the two locations that differed in greenspace. Therefore, the experience in the forest did not result in expressions that were extremely different from experience in the promenade. Instead, forest visitors showed less disgusted expressions than those in the promenade. Disgust is a type of negative emotion that is less extreme than sadness. Therefore, our results confer findings that more negative emotions can be reduced in forests than in built-up regions [10–12] according to the reason that people in cities elicited more negative emotions in the form of disgust than compared to those in forests. Wei et al. [24] also found that people in an urban forest park near the center of a city would show more negative expressions than those in forest parks in remote rural regions. Eigenvalues of the neutral scores were positively correlated with happy scores and both were inverse to sad scores, which suggests that people generally perceived net positive emotion scores in forests. Results of Wei et al. [25] concur with our finding in that people in forests showed more happiness than in an urban street and PRI. We can accept our second hypothesis.

Both men and women showed less happy expressions in urban locations than in forests, supporting the above-mentioned higher happy scores in the forest. Women also showed more positive emotions from a forest experience than in the city. These results concur with findings of Wei et al. [24] who also reported that women showed more positive expressions than men. Our results also revealed that women in forests showed lower surprised and disgusted expressions than those in the city. Negative expressions of men were not statistically different. These findings suggest that women are more sensitive and responsive than men, and thus showed more positive and negative expressions in forests and promenades, respectively. This corroborates a previous investigation which indicated that healthy men showed reduced emotion processing efficiency relative to women [52]. However, men reacted to forest locations by processing highly efficient angry expressions while women did show any differences in angry expressions between urban and forest locations. This did not lead to a divergence of angry expressions between urban and forest locations; hence men’s higher angry score in forests was the result of their psychological response. A review using a functional-evolutionary analysis indicated that it is more advantageous for men to show angry facial expression as it signals dominance, averts aggression, and deters mate poaching; it is more advantageous for women to display happy facial expressions as it signals their willingness for childcare, tending, and befriending [53].

There was a negative relationship between neutral expressions and disgusted and surprised expressions. There was a higher neutral expressions score in forests than in promenades in accordance with less disgust and surprise expressions. From this, we concluded that people in forests are more calm than in the city, whereas the excited emotion was easily confused with, and represented as, a disgusted expression. It is the peaceful environment with abundant green color and moisture in the forests that caused the calm feeling expressed as neutral faces for visitors in forests [25].

### Limits of the study

Because of accuracy, we excluded ratings on contempt and scared expressions from the analysis. It may be hard to obtain desired accuracy in recognizing contempt expressions until deep learning techniques can improve upon current limitations. A scared emotion is an important facial expression that frequently emerges in daily life. Some other instruments, such as E-Prime [44] and FaceReader [16], were reported to recognize fear in face reading at an accuracy as high as 80%, and deserves to be used in future studies to test the hit ratio for our database on Asian faces in urban forest parks. We turned to experts to help classify the typical facial expressions from our dataset. This is a useful approach to document different types of expressions. However, a more reasonable way is to classify the facial expressions by running the same photo through different software. The average score across results from different machines will provide a more reliable identification of emotions than human perception can. Furthermore, we did not discuss the interaction between gender and age on facial expressions because they do not match the theme of this study and our study results cannot support a deeper analysis on this relationship. Finally, only two locations were employed in this study, which is enough to support a frontier study on validation and assessment. Although we employed practical methodology with validation, our data still may suffer uncertainties from collection and unexpected errors. It is likely that more tests on dataset from more cities and locations would increase accuracy with the results. The inaccuracy of matching human and machine-recognized scores will be reduced with the increase of object number. Future work is encouraged to build upon this study and include additional demographic data.

## Conclusions

We compared the accuracies to match the recognized facial expressions given by FireFACE with those assessed by other instruments or artificial approach. Facial expressions of angry, disgusted, happy, sad, neutral, and surprised emotions passed the validation test because their scores were within a level of statistical acceptance. However, contempt and scared expression scores were too low and these were excluded from further analysis. We collected a total of 2,886 photos from visitors in constructed urban forests and in a promenade during summertime in Shenyang, Northeast China. There were no extreme differences in emotional expressions between forest and urban locations. A gender interaction with location showed that women exhibited more positive, but less negative, expressions in forests than in the promenade. Synthesizing these findings, we suggest that women visit constructed urban forest parks more often to elicit greater happiness and decreased negative emotions.

## Supporting information

S1 Raw data (PDF)
