# Supplementary material for: Facial expressions of Asian people exposed to constructed urban forests: Accuracy validation and variation assessment
Source: PLoS One. 2021 Jun 17;16(6):e0253141. doi: 10.1371/journal.pone.0253141 (PMC8211262; doi:10.1371/journal.pone.0253141)
Supplement: S1 Raw data — (PDF) [file pone.0253141.s001.pdf]

Number	Gender	Age	Location	Neutral	Happy	Sad	Angry
1	Female	Middle	Forest	18.71	0.47	52.73	7.54
2	Female	Middle	Forest	85.99	1.3	9.86	1.57
3	Female	Middle	Forest	10.34	0.65	68.95	2.52
4	Female	Middle	Forest	8.33	0.39	72.29	5
5	Female	Middle	Forest	5.56	0.47	18.08	54.71
6	Female	Middle	Forest	3.11	0.02	25.27	36.66
7	Female	Middle	Forest	38.43	1.89	31.07	10.14
8	Female	Middle	Forest	53.6	0.09	30.5	10.44
9	Female	Middle	Forest	3.64	0.01	34.24	41.05
10	Female	Middle	Forest	3.31	0.87	44.04	25.44
11	Female	Middle	Forest	26.5	15.92	17.21	10.58
12	Female	Middle	Forest	2.48	0.49	34.25	32.48
13	Female	Middle	Forest	49.53	0.66	34.59	3.36
14	Female	Middle	Forest	1.04	0.22	32.69	13.69
15	Female	Middle	Forest	4.7	1.03	13.04	69.36
16	Female	Middle	Forest	7.45	31.62	17.84	15.07
17	Female	Middle	Forest	49.48	31.72	5.85	9.26
18	Female	Middle	Forest	11.73	5.45	4.03	56.84
19	Female	Middle	Forest	19.36	0.7	57.23	7.28
20	Female	Middle	Forest	0	0	0	0
21	Female	Middle	Forest	13.59	4.61	11.75	44.53
22	Female	Middle	Forest	33.03	23.7	8.56	10.91
23	Female	Middle	Forest	5.67	47.12	11.44	22.67
24	Female	Middle	Forest	68.35	0.02	24.35	5.32
25	Female	Middle	Forest	9.52	0.09	67.74	9.93
26	Female	Middle	Forest	18.16	0.47	50.92	10.08
27	Female	Middle	Forest	11.92	0.11	12.08	15.71
28	Female	Middle	Forest	3.72	0.01	45.77	12.71
29	Female	Middle	Forest	8.46	2.04	22.98	29.08
30	Female	Middle	Forest	34.96	0.74	14.15	8.93
31	Female	Middle	Forest	15.58	1.28	62.74	6.35
32	Female	Middle	Forest	12.18	0.65	40.66	21.94
33	Female	Middle	Forest	25.97	41.42	7.56	17.88
34	Female	Middle	Forest	9.65	19.78	61.29	3.32
35	Female	Middle	Forest	4.38	0.02	50.66	26.8
36	Female	Middle	Forest	79.06	0.08	16.04	2.71
37	Female	Middle	Forest	64.02	0.51	10.9	5.78
38	Female	Middle	Forest	26.02	0.05	37.94	22.01
39	Female	Middle	Forest	0.74	3.27	5.1	17.44
40	Female	Middle	Forest	10.32	0.13	38.69	1.84
41	Female	Toddler	Forest	5.77	0.75	8.23	5.29
42	Female	Toddler	Forest	39.96	0.16	23.49	9.8
43	Female	Toddler	Forest	24.01	0.14	4.84	46.77
44	Female	Old	Forest	0	0	0	0
45	Female	Old	Forest	26.09	0.51	31.77	20.57
46	Female	Old	Forest	26.3	1.58	30.84	27.23
47	Female	Old	Forest	0.92	7.8	4.89	38.96
48	Female	Old	Forest	27.01	0.12	27.97	18.28
49	Female	Old	Forest	6.7	1.81	44.79	14.01
50	Female	Old	Forest	40.44	10.46	23.79	18.03
51	Female	Old	Forest	20.29	1.81	43.82	1.08
52	Female	Old	Forest	24.82	17.28	41.49	6.37
53	Female	Old	Forest	15.74	3.52	44.86	12.86
54	Female	Old	Forest	15.32	0.21	41.82	7.62
55	Female	Old	Forest	20.27	29.74	40.68	3.08
56	Female	Old	Forest	0	0	0	0

57	Female	Old	Forest	13.09	1.67	33.15	39.09
58	Female	Old	Forest	24.84	62.88	7.88	1.37
59	Female	Old	Forest	12.83	35.73	30.01	8.85
60	Female	Old	Forest	33.78	19.98	40.54	4.19
61	Female	Old	Forest	24.35	8.26	24.94	8.4
62	Female	Youth	Forest	16.07	8.19	42.96	8.64
63	Female	Youth	Forest	8.04	2.89	14.21	10.34
64	Female	Youth	Forest	7.2	0.29	44.88	27.62
65	Female	Youth	Forest	34.33	0.26	18.49	29.55
66	Female	Youth	Forest	14.11	0.36	26.08	11.07
67	Female	Youth	Forest	34.49	2.83	50.73	2.26
68	Female	Youth	Forest	4.79	0.1	37.03	31.75
69	Female	Youth	Forest	6.9	12.15	3.89	32.82
70	Female	Youth	Forest	0	0	0	0
71	Male	Middle	Forest	19.13	0.24	25.98	25.9
72	Male	Middle	Forest	2.81	0.66	61.33	18.4
73	Male	Middle	Forest	1.15	12.04	24.73	30.39
74	Male	Middle	Forest	0.13	60.8	1.1	25.39
75	Male	Middle	Forest	21.71	0.16	61.64	9.79
76	Male	Middle	Forest	6.1	0.01	18.61	69.11
77	Male	Middle	Forest	47.62	0.3	22.76	14.34
78	Male	Middle	Forest	1.24	6.6	27.65	19.87
79	Male	Middle	Forest	33.76	0.15	15.09	33.94
80	Male	Middle	Forest	53.92	6.83	20.21	10.51
81	Male	Middle	Forest	32.87	0.79	36.69	19.34
82	Male	Middle	Forest	25.26	0.91	9.88	51.77
83	Male	Middle	Forest	36.81	0.68	14.19	29.55
84	Male	Middle	Forest	21.11	11.27	12.42	37.51
85	Male	Middle	Forest	34.57	2.2	25.74	28.22
86	Male	Middle	Forest	10.04	0.13	46.34	17.29
87	Male	Middle	Forest	0	0	0	0
88	Male	Middle	Forest	0	0	0	0
89	Male	Middle	Forest	36.72	3.22	17.61	22.96
90	Male	Middle	Forest	8.82	42.94	25.17	8.22
91	Male	Middle	Forest	18.13	74.79	2.95	0.81
92	Male	Middle	Forest	12.05	0.28	14.52	46.89
93	Male	Middle	Forest	62.94	0.89	8.13	7.32
94	Male	Middle	Forest	3.09	0.05	6.79	79.06
95	Male	Middle	Forest	26.54	0.13	53.45	12.73
96	Male	Middle	Forest	3.72	0	18.75	33.89
97	Male	Middle	Forest	18.77	0.65	10.81	50.81
98	Male	Middle	Forest	34.89	0.16	21.84	32.02
99	Male	Middle	Forest	16.25	0.54	13.3	28.18
100	Male	Middle	Forest	0	0	0	0
101	Male	Middle	Forest	50.95	2.56	17.48	14.96
102	Male	Middle	Forest	31.15	0.07	10.84	46.37
103	Male	Middle	Forest	34.88	0.59	22.08	24.27
104	Male	Toddler	Forest	1.32	72.5	2.91	16.32
105	Male	Old	Forest	0	0	0	0
106	Male	Old	Forest	68.96	0.34	11.05	12.39
107	Male	Old	Forest	6.95	2.25	16.67	21.89
108	Male	Old	Forest	25.47	3.51	51.74	10.36
109	Male	Old	Forest	5.71	0.35	10.81	19.24
110	Male	Old	Forest	0	0	0	0
111	Male	Old	Forest	7.18	0.39	40.92	18.04
112	Male	Old	Forest	3.73	0.62	38.31	21.41
113	Male	Old	Forest	48.32	4.37	30	10.07

114	Male	Old	Forest	43.66	0.07	29.84	15.46
115	Male	Old	Forest	22.98	0.33	24.1	30.67
116	Male	Old	Forest	23.09	7.44	31.17	24.27
117	Male	Old	Forest	0	0	0	0
118	Male	Old	Forest	16.04	0.17	19.12	44.61
119	Male	Old	Forest	6.6	0.13	9.06	61.9
120	Male	Old	Forest	61.67	0.02	13.82	20.59
121	Male	Youth	Forest	16.09	0.52	37.42	13.89
122	Male	Youth	Forest	50.62	26.86	2.65	15.08
123	Male	Youth	Forest	38.77	1.66	23.11	11.55
124	Male	Youth	Forest	7.17	0.11	48.8	13.62
125	Male	Youth	Forest	9.11	0.33	11.37	63.98
126	Male	Youth	Forest	29.05	1.29	44.88	9.04
127	Male	Youth	Forest	9.42	0.04	49.05	32.58
128	Male	Youth	Forest	15.9	0.03	41.09	24.64
129	Male	Youth	Forest	0	0	0	0
130	Male	Youth	Forest	0	0	0	0
131	Male	Youth	Forest	20.77	0.47	31.56	34.47
132	Male	Youth	Forest	8.05	0.53	47.11	29.78
133	Male	Youth	Forest	9	0.01	23.53	57.21
134	Male	Youth	Forest	71.48	0.08	14.55	8.91
135	Male	Youth	Forest	4.3	0.02	59.83	11.39
136	Male	Youth	Forest	8.24	0	48.09	32.35
137	Male	Youth	Forest	18.61	1.55	17.4	29.87
138	Male	Youth	Forest	3.94	0.14	40.62	10.04
139	Male	Youth	Forest	25.23	0.16	23.39	36.15
140	Male	Youth	Forest	9.53	0.2	11.91	30.44
141	Male	Youth	Forest	5.88	0.71	24.71	32.4
142	Male	Youth	Forest	8.75	0.12	31.59	22.25
143	Female	Middle	Forest	66.54	2.38	25.38	3.02
144	Female	Middle	Forest	7.22	9.39	56.34	3.62
145	Female	Middle	Forest	19.95	14.49	19.44	8.28
146	Female	Middle	Forest	53.35	1.08	16.55	20.44
147	Female	Middle	Forest	41.38	5.11	8.22	14.82
148	Female	Middle	Forest	12.66	0.68	43.21	17.83
149	Female	Middle	Forest	2.37	79.97	13.38	1.29
150	Female	Middle	Forest	41.05	0.05	44.57	4.22
151	Female	Middle	Forest	33.78	0.32	45.81	4.31
152	Female	Middle	Forest	38.08	0.47	36.71	5.79
153	Female	Middle	Forest	3.33	0.94	37.68	17.2
154	Female	Middle	Forest	1.32	0.26	38.23	32.54
155	Female	Middle	Forest	14.83	3.75	48.51	16.99
156	Female	Middle	Forest	30.54	0.03	38.01	19.26
157	Female	Middle	Forest	47.82	7.56	31.26	6.42
158	Female	Middle	Forest	0	0	0	0
159	Female	Middle	Forest	74.71	2.11	10.19	8.14
160	Female	Middle	Forest	28.04	9.75	18.77	6.7
161	Female	Middle	Forest	9.73	0.57	17.82	8.6
162	Female	Middle	Forest	28.95	32.42	23.94	7.16
163	Female	Middle	Forest	35.62	8.55	38.24	12.74
164	Female	Middle	Forest	11.99	0.21	22.7	1.79
165	Female	Middle	Forest	44.41	11.87	23.29	6.8
166	Female	Middle	Forest	12.1	0.89	38.54	21.99
167	Female	Middle	Forest	12.23	0.38	32.71	21.36
168	Female	Middle	Forest	17.86	0.11	30.05	29.36
169	Female	Middle	Forest	28.86	0.18	34.17	0.98
170	Female	Middle	Forest	7.19	2.82	41.26	27.08

171	Female	Toddler	Forest	0	0	0	0
172	Female	Old	Forest	0.95	0.39	38.18	50.18
173	Female	Old	Forest	7.62	0.04	21.38	59.88
174	Female	Old	Forest	0	0	0	0
175	Female	Old	Forest	6.72	75.22	8.43	7.48
176	Female	Old	Forest	0	0	0	0
177	Female	Old	Forest	7.39	0.8	31.09	43.53
178	Female	Old	Forest	3.95	33.84	7.07	7.94
179	Female	Old	Forest	15.99	0.25	52.65	8.87
180	Female	Old	Forest	49.99	0.59	29.15	8.04
181	Female	Old	Forest	6.8	0.48	75.79	8.13
182	Female	Old	Forest	10.18	0.46	61.88	14.56
183	Female	Old	Forest	0	0	0	0
184	Female	Old	Forest	29.39	15.85	36.45	12.77
185	Female	Youth	Forest	9.7	20.22	33.28	6.42
186	Female	Youth	Forest	17.01	1.9	55.12	11.65
187	Female	Youth	Forest	86.79	0.34	9.27	1.59
188	Female	Youth	Forest	14.29	36.62	11.9	14.1
189	Female	Youth	Forest	3.12	2.86	25	38.33
190	Female	Youth	Forest	15.59	1.58	50.38	14.18
191	Female	Youth	Forest	12.64	0.46	60.07	7.89
192	Female	Youth	Forest	92.18	0.45	3.15	2.09
193	Female	Youth	Forest	25.82	0.13	28.05	19.03
194	Female	Youth	Forest	10.63	0.06	4.87	1.7
195	Female	Youth	Forest	88.79	0.08	4.56	3.36
196	Female	Youth	Forest	39.65	0.17	26.55	14.13
197	Female	Youth	Forest	51.09	27.64	12.53	4.75
198	Male	Middle	Forest	13.6	2.96	48.54	18.65
199	Male	Middle	Forest	14.1	0.12	43.79	9.15
200	Male	Middle	Forest	29.57	0.82	18.94	30.66
201	Male	Middle	Forest	25.75	9.21	19.63	24
202	Male	Middle	Forest	5.31	21.13	35.68	18.88
203	Male	Middle	Forest	9.19	83.81	4.5	0.52
204	Male	Middle	Forest	17.77	4.42	46.77	21.14
205	Male	Middle	Forest	1.73	73.85	4.8	13.03
206	Male	Middle	Forest	83.35	0.77	7.44	7.07
207	Male	Middle	Forest	14.44	5.07	65.54	8.11
208	Male	Middle	Forest	63.87	0.03	30.03	3.45
209	Male	Middle	Forest	58.85	9.13	12.33	6.76
210	Male	Middle	Forest	16.8	4.37	39.35	13.79
211	Male	Middle	Forest	2.25	69.68	20.08	1.73
212	Male	Middle	Forest	10.51	10.22	44.58	19.34
213	Male	Middle	Forest	24.19	0.19	46.2	17.05
214	Male	Middle	Forest	34.99	0.08	28.4	10.2
215	Male	Middle	Forest	68.2	5.14	5.28	14.43
216	Male	Toddler	Forest	3.67	0.1	53.82	15.86
217	Male	Toddler	Forest	97.16	0.05	0.98	0.88
218	Male	Toddler	Forest	50.51	0.1	14.87	16.3
219	Male	Old	Forest	3.21	44.51	6.19	35.05
220	Male	Old	Forest	10.05	63.07	21.83	3.37
221	Male	Old	Forest	37.03	37.8	12.84	8.04
222	Male	Old	Forest	24.19	2	26.48	37.26
223	Male	Old	Forest	6.98	41.34	38.36	3.98
224	Male	Old	Forest	7.7	0.36	47.61	25.42
225	Male	Old	Forest	0	0	0	0
226	Male	Old	Forest	12.41	6.18	10.59	17.7
227	Male	Old	Forest	75.89	0.86	8.69	12.32

228	Male	Old	Forest	9.26	3.51	37.44	36.02
229	Male	Old	Forest	20.41	30.04	31.89	3.24
230	Male	Old	Forest	40.86	6.19	39.82	1.36
231	Male	Old	Forest	3.32	7.1	70.2	5.99
232	Male	Old	Forest	4.22	0.23	34.1	26.45
233	Male	Old	Forest	0	0	0	0
234	Male	Old	Forest	40.06	15.91	26.03	7.34
235	Male	Old	Forest	0	0	0	0
236	Male	Old	Forest	28.36	0.3	11.27	47.59
237	Male	Youth	Forest	0	0	0	0
238	Male	Youth	Forest	19.13	59.88	4.26	10.88
239	Male	Youth	Forest	87.11	0.21	6.07	4.35
240	Male	Youth	Forest	22.86	2.8	47.47	7.59
241	Male	Youth	Forest	70.48	0.66	4.92	22
242	Male	Youth	Forest	0	0	0	0
243	Male	Youth	Forest	54.15	2.04	14.77	19.3
244	Male	Youth	Forest	33.66	0.99	15.55	29.68
245	Male	Youth	Forest	45.22	0.11	19.22	16.52
246	Male	Youth	Forest	53.73	0.05	20.79	10.03
247	Male	Youth	Forest	13.17	0.33	55.09	15.42
248	Male	Youth	Forest	21.65	0.14	19.18	11.64
249	Female	Middle	Forest	0.8	5.6	28.14	21.49
250	Female	Middle	Forest	4.41	18.28	33.48	21.44
251	Female	Middle	Forest	17.66	3.65	13.6	31.06
252	Female	Middle	Forest	25.59	0.9	45.25	22.01
253	Female	Middle	Forest	10.66	0.63	45.67	11.91
254	Female	Middle	Forest	25.09	1.36	30.16	21.3
255	Female	Middle	Forest	8.25	0.43	15.07	70.76
256	Female	Middle	Forest	15.13	0.17	64.33	10.15
257	Female	Middle	Forest	5.3	1.47	77.34	9.11
258	Female	Middle	Forest	44.18	1.9	40.5	8.28
259	Female	Middle	Forest	26.38	1.12	54.48	7.27
260	Female	Middle	Forest	7.92	0.06	20.74	60.4
261	Female	Middle	Forest	9.83	0.45	79.01	3.22
262	Female	Middle	Forest	15.44	0.77	47.87	11.36
263	Female	Middle	Forest	0.48	0.89	0.76	83.61
264	Female	Middle	Forest	15.46	0.97	49.45	5.71
265	Female	Middle	Forest	30.26	0.47	33.13	13.84
266	Female	Middle	Forest	17.46	1.76	64.41	5.06
267	Female	Middle	Forest	37.36	3.79	22.96	20.18
268	Female	Middle	Forest	5.34	0.06	63.44	12.52
269	Female	Middle	Forest	23.94	0.4	59.47	8.02
270	Female	Middle	Forest	60.35	7.07	28	1.7
271	Female	Middle	Forest	29.74	0.77	53.3	10.08
272	Female	Middle	Forest	5.4	14.63	63.41	3.25
273	Female	Middle	Forest	40.7	0.02	32.34	15.51
274	Female	Middle	Forest	44.78	0.01	41.03	8.26
275	Female	Middle	Forest	6.27	0.07	32.78	32.91
276	Female	Middle	Forest	53.57	1.45	27	6.53
277	Female	Middle	Forest	0	0	0	0
278	Female	Middle	Forest	17.97	0.03	45.23	3.57
279	Female	Middle	Forest	2.95	5.56	77.54	7.26
280	Female	Middle	Forest	20.09	14.17	52.28	1.79
281	Female	Middle	Forest	20.4	66.03	9.33	0.59
282	Female	Middle	Forest	30.4	0.45	48.38	6.65
283	Female	Middle	Forest	10.34	7.53	29.24	21.45
284	Female	Middle	Forest	12.19	0.1	11.54	16.22

285	Female	Middle	Forest	10.19	0.18	31.16	16.24
286	Female	Middle	Forest	23.18	0.04	4.62	22.98
287	Female	Middle	Forest	9.8	6.82	49.57	4.15
288	Female	Middle	Forest	28.51	1.47	39.77	9.96
289	Female	Middle	Forest	16.64	1.28	47.77	5.8
290	Female	Middle	Forest	3.38	0.73	12.96	2.06
291	Female	Middle	Forest	8.03	44.47	14.73	3.58
292	Female	Middle	Forest	63.91	0.89	20.18	9.05
293	Female	Middle	Forest	79.26	0.66	15.05	1.43
294	Female	Middle	Forest	25.16	0.04	38.87	20.31
295	Female	Middle	Forest	3.3	9.24	15.77	5.05
296	Female	Middle	Forest	0	0	0	0
297	Female	Middle	Forest	40.13	2.11	24.05	15.86
298	Female	Middle	Forest	38.52	1.06	24.71	22.09
299	Female	Middle	Forest	47.75	5.16	43.33	0.95
300	Female	Middle	Forest	82.92	1	14.72	0.65
301	Female	Middle	Forest	12.18	5.3	27.58	16.84
302	Female	Middle	Forest	11.03	5.35	36.65	19.61
303	Female	Middle	Forest	39.86	0.51	34.25	14
304	Female	Toddler	Forest	86.71	0.86	10.44	0.63
305	Female	Toddler	Forest	6.56	0.53	51.49	12.72
306	Female	Toddler	Forest	93.44	0.72	1.89	3.43
307	Female	Old	Forest	0	0	0	0
308	Female	Old	Forest	25.74	1.6	53.73	12.34
309	Female	Old	Forest	44.47	0.18	22.6	15.16
310	Female	Old	Forest	14.4	0.16	33.87	31.59
311	Female	Old	Forest	22.75	58.47	3.9	10.91
312	Female	Old	Forest	4.08	0.89	77.68	6.39
313	Female	Old	Forest	11.99	20.3	47.4	7.98
314	Female	Old	Forest	15.29	10.94	56.34	7.3
315	Female	Old	Forest	8.17	1.06	66.32	9.68
316	Female	Old	Forest	29.54	1.52	37.62	12.75
317	Female	Old	Forest	53.1	3.95	32.66	6.73
318	Female	Youth	Forest	66.32	20.38	11.96	0.26
319	Female	Youth	Forest	5.78	0.07	27.34	40.23
320	Female	Youth	Forest	13.93	0.79	27.67	15.57
321	Female	Youth	Forest	73.32	0.12	25.58	0.38
322	Female	Youth	Forest	55.38	3.43	34.37	4.6
323	Female	Youth	Forest	1.83	0.36	10.84	14.02
324	Female	Youth	Forest	4.97	0.01	43.95	22.83
325	Female	Youth	Forest	14.65	0.03	47.42	17.93
326	Female	Youth	Forest	44.29	1.45	35.55	7.37
327	Female	Youth	Forest	64.32	0.06	25.81	7.22
328	Female	Youth	Forest	40.45	11.42	22.29	16.96
329	Female	Youth	Forest	0.42	0.32	2.07	3.24
330	Female	Youth	Forest	51.37	0.61	17.03	21.15
331	Female	Youth	Forest	29.21	2.08	50.65	10.57
332	Female	Youth	Forest	4.24	0.04	9.4	9.67
333	Female	Youth	Forest	5.27	0.01	45.36	10.23
334	Female	Youth	Forest	9.51	3.3	29.67	24.44
335	Female	Youth	Forest	27.91	0.8	50.98	9.39
336	Female	Youth	Forest	0.74	14.5	0.49	81.88
337	Female	Youth	Forest	3.73	0.01	31.33	24.27
338	Female	Youth	Forest	11.88	0.27	62.83	6.75
339	Female	Youth	Forest	23.77	0.53	48.98	17.78
340	Female	Youth	Forest	0	0	0	0
341	Male	Middle	Forest	7.87	2.26	15.01	45.68

342	Male	Middle	Forest	2.94	1.51	55.05	21.18
343	Male	Middle	Forest	20.05	3.6	39.61	19.15
344	Male	Middle	Forest	0	0	0	0
345	Male	Middle	Forest	29.87	2.24	48.08	12.06
346	Male	Middle	Forest	37.28	1.47	35.85	14.18
347	Male	Middle	Forest	18.63	27.23	42.97	2.35
348	Male	Middle	Forest	43.42	1.51	32.72	6.42
349	Male	Middle	Forest	3.85	12.77	33.04	11.53
350	Male	Middle	Forest	20.84	0.04	46.3	26.32
351	Male	Middle	Forest	7.3	0.36	20.15	43.69
352	Male	Middle	Forest	32.94	55.98	6.06	1.91
353	Male	Middle	Forest	22.44	0.57	26.52	28.43
354	Male	Middle	Forest	3.63	6.51	42.46	7.49
355	Male	Middle	Forest	27.84	0.07	47.35	19.71
356	Male	Middle	Forest	45.6	1.41	33.71	10.24
357	Male	Middle	Forest	81.35	0.02	6.23	10.35
358	Male	Middle	Forest	39.12	24.72	11.36	8.19
359	Male	Middle	Forest	32.04	0.17	22.27	35.98
360	Male	Middle	Forest	42.17	0.09	41.52	8.35
361	Male	Middle	Forest	6.79	0.2	37.56	46.53
362	Male	Middle	Forest	4.61	0.08	48.7	3.76
363	Male	Middle	Forest	9.39	78.28	8.66	1.83
364	Male	Middle	Forest	0	0	0	0
365	Male	Middle	Forest	14.84	0.44	20.62	36.48
366	Male	Middle	Forest	10.16	0.27	37.32	16.19
367	Male	Middle	Forest	17.98	2.78	50.61	6.19
368	Male	Toddler	Forest	61.58	0.09	28.49	1.3
369	Male	Toddler	Forest	24.62	4.34	8.42	47.29
370	Male	Toddler	Forest	64.46	7.67	22.79	3.03
371	Male	Toddler	Forest	39.41	21.44	30.72	5.19
372	Male	Old	Forest	40.86	6.19	39.82	1.36
373	Male	Old	Forest	39.07	33.39	19.21	1.58
374	Male	Old	Forest	12.2	2.71	31.6	29.51
375	Male	Old	Forest	30.91	0.49	41.55	16.39
376	Male	Old	Forest	43.18	8.89	43.02	1.49
377	Male	Old	Forest	14.29	61.58	19.06	1.91
378	Male	Old	Forest	0	0	0	0
379	Male	Old	Forest	15.94	0.64	52.24	5.27
380	Male	Old	Forest	16.84	1.61	33.96	30.71
381	Male	Old	Forest	0	0	0	0
382	Male	Old	Forest	42.76	12.63	35.29	4.69
383	Male	Old	Forest	7.28	0.2	40.75	29.88
384	Male	Old	Forest	16.84	47.31	32.46	2.2
385	Male	Old	Forest	65.62	0.3	22.14	6.81
386	Male	Old	Forest	29.02	0.11	29.88	27.04
387	Male	Old	Forest	26.06	0.33	17.77	53.37
388	Male	Old	Forest	65.76	0.75	20.67	6.9
389	Male	Old	Forest	4.79	0.02	49.61	24
390	Male	Old	Forest	19.74	3.67	45.01	18.89
391	Male	Old	Forest	15.34	7.44	32.02	30.91
392	Male	Old	Forest	6.76	1.73	44.36	10.32
393	Male	Old	Forest	29.16	0.05	19.49	14.24
394	Male	Old	Forest	13.37	2.88	30.93	18.75
395	Male	Old	Forest	12.81	0.08	38.71	23.34
396	Male	Old	Forest	67.06	1.9	10.2	16.99
397	Male	Old	Forest	45.26	0.41	14.5	29.85
398	Male	Old	Forest	9.93	0.18	72.21	5.23

399	Male	Old	Forest	27.81	1	44.05	21.38
400	Male	Old	Forest	15.13	0.42	61.06	8.08
401	Male	Old	Forest	7.99	0.2	63.12	12.25
402	Male	Old	Forest	3.69	6.87	47.55	11.07
403	Male	Old	Forest	2.68	0.28	25.62	36.26
404	Male	Old	Forest	26.41	20.6	27.93	14.68
405	Male	Old	Forest	42.26	0.23	48.22	2.76
406	Male	Youth	Forest	6.15	0.25	57.09	10.57
407	Male	Youth	Forest	32.47	0.27	25.22	31.82
408	Male	Youth	Forest	42.16	37.04	8.53	7.95
409	Male	Youth	Forest	7.66	1.38	14.76	59.68
410	Male	Youth	Forest	4.29	0.47	19.38	40.74
411	Male	Youth	Forest	46.37	0.16	34.38	7.61
412	Male	Youth	Forest	57.86	0.27	11.85	14.02
413	Male	Youth	Forest	55.14	1.58	11.61	21.67
414	Male	Youth	Forest	51.29	0.77	39.44	3.88
415	Male	Youth	Forest	9.15	85.35	1.49	0.49
416	Male	Youth	Forest	77.67	1.77	16.76	1.48
417	Male	Youth	Forest	12.05	24.94	11.36	27.46
418	Male	Youth	Forest	21.95	3.85	50.63	13.37
419	Male	Youth	Forest	70.96	0.35	3.73	12.43
420	Male	Youth	Forest	4.28	0.15	28.25	15.95
421	Male	Youth	Forest	25.06	0.13	21.44	38.31
422	Male	Youth	Forest	3.48	39.06	26.66	6.21
423	Female	Middle	Forest	0.58	96.63	1.06	0.39
424	Female	Middle	Forest	48.69	33.05	16.26	1.16
425	Female	Middle	Forest	0.33	93.03	1.6	3.62
426	Female	Middle	Forest	30.55	5.01	57.73	2.81
427	Female	Middle	Forest	0	0	0	0
428	Female	Middle	Forest	8.07	87.91	1.73	0.66
429	Female	Middle	Forest	1.95	97.04	0.37	0.41
430	Female	Middle	Forest	32.98	14.4	35.6	4.17
431	Female	Middle	Forest	2.04	13.48	20.21	48.95
432	Female	Middle	Forest	4.69	30.47	8.3	6.5
433	Female	Middle	Forest	1.3	47.59	2	18
434	Female	Middle	Forest	46.79	6.07	40.3	1.45
435	Female	Middle	Forest	29.29	51.6	14.04	2.41
436	Female	Middle	Forest	46.39	12.91	33.19	4.73
437	Female	Middle	Forest	15.45	2.62	18.81	47.47
438	Female	Middle	Forest	1.48	95.01	1.04	0.95
439	Female	Middle	Forest	4.77	53.34	16.59	16.24
440	Female	Middle	Forest	43.1	1.94	26.17	16.98
441	Female	Middle	Forest	27.75	46.63	11.38	5.31
442	Female	Middle	Forest	57.6	0.3	6.28	22.87
443	Female	Middle	Forest	6.44	90.02	1	0.96
444	Female	Middle	Forest	24.18	66.07	8.07	0.69
445	Female	Middle	Forest	0.99	11.48	4.58	45.41
446	Female	Middle	Forest	34.57	1.04	54.7	3.7
447	Female	Middle	Forest	2.49	93.49	2.57	0.51
448	Female	Middle	Forest	16.56	48.12	15.58	6.1
449	Female	Middle	Forest	27.69	30	8.28	14.77
450	Female	Middle	Forest	7.97	81.14	5.57	1.28
451	Female	Middle	Forest	40.91	42.58	10.09	3.05
452	Female	Middle	Forest	0.07	99.71	0.06	0.07
453	Female	Middle	Forest	68.52	7.37	10.31	5.73
454	Female	Middle	Forest	35.78	36.98	13.64	4.45
455	Female	Middle	Forest	5.11	60.58	16.61	5.79

456	Female	Middle	Forest	0.49	99.4	0.07	0.02
457	Female	Middle	Forest	1.08	94.65	2.68	0.58
458	Female	Middle	Forest	22.91	49.72	10.73	6.81
459	Female	Middle	Forest	68.89	7.35	17.74	3.8
460	Female	Middle	Forest	5.78	92.78	1.13	0.04
461	Female	Middle	Forest	4.39	38.04	23.45	8.66
462	Female	Middle	Forest	2.2	96.86	0.54	0.16
463	Female	Middle	Forest	0.18	99.16	0.22	0.22
464	Female	Middle	Forest	0	99.98	0	0.01
465	Female	Middle	Forest	89.14	0.99	5.04	2.56
466	Female	Middle	Forest	11.93	60.3	6.4	4.37
467	Female	Middle	Forest	0.55	97.49	1.52	0.1
468	Female	Middle	Forest	75.46	9.16	9.17	4.43
469	Female	Middle	Forest	4.03	90.11	2.47	0.7
470	Female	Middle	Forest	0.55	99.29	0.13	0.01
471	Female	Middle	Forest	1.11	88.27	5.29	2.21
472	Female	Middle	Forest	2.93	86.93	6.78	1.96
473	Female	Middle	Forest	0.21	99.26	0.13	0.21
474	Female	Middle	Forest	9.67	78.83	8.25	1.21
475	Female	Toddler	Forest	30.46	6.13	45.81	8.9
476	Female	Toddler	Forest	0	0	0	0
477	Female	Toddler	Forest	1.1	94.96	2.46	0.56
478	Female	Toddler	Forest	0.61	99.22	0.06	0.04
479	Female	Toddler	Forest	5.22	84.62	7.94	0.57
480	Female	Toddler	Forest	54.37	10.76	27.11	3.57
481	Female	Toddler	Forest	6.46	5.98	79.45	1.81
482	Female	Toddler	Forest	8.62	83.75	4.91	0.96
483	Female	Toddler	Forest	90.26	1.77	3.71	1.33
484	Female	Toddler	Forest	35.27	12.86	29.5	3.95
485	Female	Toddler	Forest	12.92	2.1	15.04	31.1
486	Female	Toddler	Forest	30.64	6.79	48.21	2.28
487	Female	Old	Forest	32.37	5.48	57.7	2.75
488	Female	Old	Forest	14.49	2.3	22.36	49.68
489	Female	Old	Forest	27.06	45.01	25.39	1.24
490	Female	Old	Forest	8.52	54.06	19.35	12.15
491	Female	Old	Forest	36.59	9.21	34.04	15.96
492	Female	Old	Forest	0.01	99.97	0.01	0
493	Female	Old	Forest	1.31	91.98	5.17	0.77
494	Female	Old	Forest	0	0	0	0
495	Female	Old	Forest	0.9	96.03	2.12	0.43
496	Female	Old	Forest	8.87	1.65	51.49	11.79
497	Female	Old	Forest	22.78	50.95	11.49	9.03
498	Female	Old	Forest	0	0	0	0
499	Female	Old	Forest	6.2	87.99	2.62	2.58
500	Female	Old	Forest	0	0	0	0
501	Female	Old	Forest	4.83	87.07	3.64	0.91
502	Female	Old	Forest	21.21	66.8	6.29	2.81
503	Female	Old	Forest	4.17	50.75	5.07	16.95
504	Female	Old	Forest	19.09	44.07	21.17	8.93
505	Female	Old	Forest	0.05	99.63	0.17	0.04
506	Female	Old	Forest	32.62	46.06	11.08	2.36
507	Female	Old	Forest	8.8	58.99	28.09	1.15
508	Female	Old	Forest	15.88	71.87	8.97	0.74
509	Female	Old	Forest	0.22	99.75	0.01	0
510	Female	Old	Forest	0.67	13.89	18.02	32.33
511	Female	Old	Forest	5.63	41	35.26	7.24
512	Female	Old	Forest	0.14	95.4	1.87	0.46

513	Female	Old	Forest	1.84	54.42	32.51	3.2
514	Female	Old	Forest	13.54	71.12	10.33	0.26
515	Female	Old	Forest	8.48	47.72	39.97	1.67
516	Female	Old	Forest	1.22	97.37	0.61	0.31
517	Female	Old	Forest	27.33	70.13	1.61	0.53
518	Female	Old	Forest	0.45	98.57	0.33	0.32
519	Female	Old	Forest	15.14	62.12	15.72	3.72
520	Female	Old	Forest	18.61	71.7	5.86	0.87
521	Female	Old	Forest	22.72	28.58	37.08	8.33
522	Female	Old	Forest	0.76	96.9	1.92	0.11
523	Female	Old	Forest	21.98	7.65	63.36	1.55
524	Female	Old	Forest	2.66	94.43	2.04	0.18
525	Female	Old	Forest	24.01	64.78	7.31	0.46
526	Female	Old	Forest	14.38	23.38	16.98	4.97
527	Female	Old	Forest	12.37	67.4	6.06	3.18
528	Female	Youth	Forest	17.96	48.64	15.22	5.7
529	Female	Youth	Forest	0.95	92.88	3.85	0.92
530	Female	Youth	Forest	0.46	26.3	0.75	67.67
531	Female	Youth	Forest	2.6	76.85	1.71	14.27
532	Female	Youth	Forest	83.1	1.25	11.3	2.69
533	Female	Youth	Forest	1.73	95	2.63	0.18
534	Female	Youth	Forest	0	0	0	0
535	Female	Youth	Forest	0.46	99.32	0.03	0.12
536	Female	Youth	Forest	0.86	96.99	0.33	0.45
537	Female	Youth	Forest	1.78	97.63	0.12	0.2
538	Female	Youth	Forest	30.72	60.45	5.63	1.16
539	Female	Youth	Forest	41.46	7.05	32.09	7.66
540	Female	Youth	Forest	67.56	3.08	20.73	1.64
541	Female	Youth	Forest	3.58	93.04	1.07	0.79
542	Female	Youth	Forest	0.57	72.18	5.58	9.12
543	Female	Youth	Forest	14.21	68.07	6.11	7.14
544	Female	Youth	Forest	0	0	0	0
545	Female	Youth	Forest	0.48	93.83	1.59	0.77
546	Female	Youth	Forest	5.36	21.82	1.35	58.49
547	Female	Youth	Forest	21.55	74.98	2.63	0.38
548	Female	Youth	Forest	0.05	97.14	0.06	2.08
549	Female	Youth	Forest	11.72	13.48	12	29.16
550	Female	Youth	Forest	24.97	48.23	6.4	10.79
551	Female	Youth	Forest	10.16	54.79	4.08	8.2
552	Female	Youth	Forest	0.02	99.52	0.01	0.36
553	Female	Youth	Forest	0.26	83.85	1.15	9.84
554	Female	Youth	Forest	61.25	20.18	15.54	1.45
555	Female	Youth	Forest	8.41	60.79	24.14	1.63
556	Female	Youth	Forest	2.52	90.15	2.03	1.7
557	Female	Youth	Forest	12.5	55.18	9.63	8.82
558	Female	Youth	Forest	11.2	4.13	49.36	23.1
559	Female	Youth	Forest	2.3	16.92	16.04	29.63
560	Female	Youth	Forest	0.05	99.69	0.05	0.11
561	Female	Youth	Forest	0.24	79.84	2.07	13.64
562	Female	Youth	Forest	35.81	0.96	26.46	15.84
563	Female	Youth	Forest	17.88	37.55	19.62	12.35
564	Female	Youth	Forest	0.62	93.02	0.24	4.94
565	Female	Youth	Forest	0.09	99.5	0.07	0.09
566	Female	Youth	Forest	4.34	94.07	1.05	0.17
567	Female	Youth	Forest	6.4	43.56	4.13	23.6
568	Female	Youth	Forest	3.18	34.37	5.77	48.12
569	Female	Youth	Forest	14.27	0.3	51.06	17.31

570	Female	Youth	Forest	50.21	3.41	24.57	13.96
571	Female	Youth	Forest	0.85	40.84	5.1	18.76
572	Female	Youth	Forest	0.3	99.36	0.09	0.1
573	Female	Youth	Forest	1.91	71.93	6.89	7.25
574	Female	Youth	Forest	1.34	96.76	0.66	0.8
575	Female	Youth	Forest	0.63	30.86	0.84	45.6
576	Female	Youth	Forest	0.12	97.36	0.49	0.71
577	Female	Youth	Forest	23.53	35.1	25.92	1.34
578	Female	Youth	Forest	17.97	0.26	29.31	16.18
579	Female	Youth	Forest	1.56	86.1	3.94	1.64
580	Female	Youth	Forest	0.25	97.85	0.25	0.98
581	Female	Youth	Forest	57.93	0.54	19.89	16.51
582	Female	Youth	Forest	30.67	37.74	15.6	4.93
583	Female	Youth	Forest	0	0	0	0
584	Female	Youth	Forest	0.71	95.85	1.48	0.8
585	Female	Youth	Forest	13.33	54.01	17.22	5.22
586	Female	Youth	Forest	2.82	90.19	2.47	0.58
587	Female	Youth	Forest	26.22	57.06	14.03	1.1
588	Female	Youth	Forest	9.28	79.13	4.63	0.9
589	Female	Youth	Forest	22.54	12.65	32.96	6.51
590	Female	Youth	Forest	0.02	99.59	0.15	0.03
591	Female	Youth	Forest	0.22	98.52	1.1	0.03
592	Female	Youth	Forest	10.26	46.82	10.8	2.25
593	Female	Youth	Forest	4.91	69.05	16.46	1.64
594	Female	Youth	Forest	7.93	56.03	6.12	9.77
595	Female	Youth	Forest	0.01	99.98	0.01	0
596	Female	Youth	Forest	0.11	99.8	0.02	0.04
597	Female	Youth	Forest	11.36	81.44	1.06	3.35
598	Female	Youth	Forest	0.17	99.63	0.08	0.04
599	Female	Youth	Forest	33.4	5.59	54.78	2.29
600	Female	Youth	Forest	58.27	15.92	14.74	3.8
601	Female	Youth	Forest	28.3	13.34	33.11	3.05
602	Female	Youth	Forest	73.54	13.24	4.87	2.61
603	Female	Youth	Forest	0.19	99.78	0.01	0
604	Female	Youth	Forest	48.73	3.28	28.57	3.32
605	Female	Youth	Forest	3.71	88.24	2.65	2.03
606	Female	Youth	Forest	36.65	10.38	19.01	22.8
607	Female	Youth	Forest	9.17	47.16	8.8	11.9
608	Female	Youth	Forest	5.7	66.79	12.18	10.64
609	Female	Youth	Forest	5.81	83.74	4	1.72
610	Female	Youth	Forest	4.22	81.83	3.8	5.84
611	Male	Middle	Forest	0	0	0	0
612	Male	Middle	Forest	5.35	44.72	26.42	6.98
613	Male	Middle	Forest	6.5	3.83	74.6	9.25
614	Male	Middle	Forest	3.77	94.39	0.45	0.57
615	Male	Middle	Forest	7.44	84.64	4.46	1.98
616	Male	Middle	Forest	0.16	99.49	0.18	0.12
617	Male	Middle	Forest	19.59	11.11	34.51	17.23
618	Male	Middle	Forest	35.68	59.46	3.64	0.59
619	Male	Middle	Forest	6.76	46.88	18.86	20.26
620	Male	Middle	Forest	2.22	94.56	1.03	0.88
621	Male	Middle	Forest	27.87	54.41	2.11	12.06
622	Male	Middle	Forest	2.58	94.53	1.89	0.52
623	Male	Middle	Forest	3.9	1.6	68.43	5.86
624	Male	Middle	Forest	0.01	98.44	0.07	0.71
625	Male	Middle	Forest	26.79	34.49	15.52	14.08
626	Male	Middle	Forest	9.24	21.08	49.76	3.15

627	Male	Middle	Forest	1.63	96.56	0.74	0.38
628	Male	Middle	Forest	4.06	93.58	1.4	0.14
629	Male	Middle	Forest	76.86	16.6	2.18	3.25
630	Male	Middle	Forest	18.49	74.73	4.66	0.3
631	Male	Middle	Forest	12.21	63.94	10.72	8.84
632	Male	Middle	Forest	6.47	65.14	11.56	5.07
633	Male	Middle	Forest	0.42	85.22	0.13	8.32
634	Male	Middle	Forest	3.84	87.34	1.4	2.46
635	Male	Middle	Forest	7.2	26.06	43.78	7.29
636	Male	Middle	Forest	95.02	0.89	2.39	1.09
637	Male	Middle	Forest	6.42	71.9	7.44	7.27
638	Male	Middle	Forest	14.91	19.42	30.81	11.78
639	Male	Middle	Forest	24.27	46.77	17.44	5.47
640	Male	Middle	Forest	39.14	36.97	15.89	2.11
641	Male	Middle	Forest	23.57	3.01	45.94	13.62
642	Male	Middle	Forest	7.28	5.06	69.14	6.76
643	Male	Middle	Forest	23.65	51.6	5.5	7.46
644	Male	Middle	Forest	1.66	96.28	1.74	0.06
645	Male	Middle	Forest	2.08	73.47	4.03	12.78
646	Male	Middle	Forest	3.22	81.24	9.85	0.81
647	Male	Middle	Forest	0.06	99.65	0.1	0.1
648	Male	Middle	Forest	3.56	28.39	56.6	2.22
649	Male	Toddler	Forest	1.22	71.49	15.14	1.05
650	Male	Toddler	Forest	86.47	2.28	6.51	3
651	Male	Toddler	Forest	2.3	90.1	3.9	2.17
652	Male	Toddler	Forest	55.29	12.63	27.35	1.73
653	Male	Toddler	Forest	0	0	0	0
654	Male	Toddler	Forest	8.16	57.49	12.61	14.56
655	Male	Toddler	Forest	17.55	11.67	27.19	19.42
656	Male	Toddler	Forest	13.3	86.04	0.31	0.03
657	Male	Toddler	Forest	0.03	99.67	0.02	0.13
658	Male	Old	Forest	3.95	73.19	14.04	1.97
659	Male	Old	Forest	34.99	45.38	10.51	3.94
660	Male	Old	Forest	30.33	9.3	47.63	1.86
661	Male	Old	Forest	8.24	68.66	8.04	1.62
662	Male	Old	Forest	20.67	56.42	19.31	1.75
663	Male	Old	Forest	20.13	8.33	50.59	11.51
664	Male	Old	Forest	4.17	49.87	33.48	1.84
665	Male	Old	Forest	0	0	0	0
666	Male	Old	Forest	19.62	12.47	41.88	10.71
667	Male	Old	Forest	1.9	34.5	5.86	49.02
668	Male	Old	Forest	2.34	96.5	0.88	0.08
669	Male	Old	Forest	5.52	64.97	10.6	6.16
670	Male	Old	Forest	7.22	71.88	0.13	16.25
671	Male	Old	Forest	0	0	0	0
672	Male	Old	Forest	1.66	86.36	9.18	1.38
673	Male	Old	Forest	19.34	47.29	8.39	17.94
674	Male	Old	Forest	46.47	22.46	20.86	5.94
675	Male	Old	Forest	0	0	0	0
676	Male	Old	Forest	30.4	5.46	29.61	13.39
677	Male	Old	Forest	0	0	0	0
678	Male	Youth	Forest	2.39	56.93	11.64	8.65
679	Male	Youth	Forest	23.45	48.24	9.29	9.48
680	Male	Youth	Forest	4.11	94.33	0.2	0.18
681	Male	Youth	Forest	6.98	84.81	3.67	2.77
682	Male	Youth	Forest	30.01	1.92	43.88	8.6
683	Male	Youth	Forest	4.77	93.06	1.66	0.19

684	Male	Youth	Forest	69.16	0.72	13.15	9
685	Male	Youth	Forest	80.48	6.39	11.22	0.69
686	Male	Youth	Forest	32.73	0.16	34.34	21.04
687	Male	Youth	Forest	20.03	27.34	42.2	3.33
688	Male	Youth	Forest	18.63	61.92	0.51	2.28
689	Male	Youth	Forest	0.68	99.25	0.03	0.02
690	Male	Youth	Forest	4.91	4.62	6.41	34.6
691	Male	Youth	Forest	29.34	25.77	10.87	18.32
692	Male	Youth	Forest	6.16	89.64	0.48	2.46
693	Male	Youth	Forest	4.5	65.36	2.62	16.15
694	Male	Youth	Forest	7.75	86.37	3.55	1.54
695	Male	Youth	Forest	0	0	0	0
696	Male	Youth	Forest	13.14	80.37	2.39	2.75
697	Male	Youth	Forest	45.24	31.95	8.42	6.53
698	Male	Youth	Forest	59.08	34.75	1.4	0.94
699	Male	Youth	Forest	0.19	99.54	0.06	0.09
700	Male	Youth	Forest	26.34	30.02	22.75	18.9
701	Male	Youth	Forest	43.36	2.58	11.89	20.17
702	Male	Youth	Forest	0	0	0	0
703	Male	Youth	Forest	75.88	22.87	0.49	0.33
704	Male	Youth	Forest	0	0	0	0
705	Male	Youth	Forest	0	0	0	0
706	Male	Youth	Forest	10.25	86.23	0.94	0.83
707	Male	Youth	Forest	20.13	68.76	4.42	3.03
708	Male	Youth	Forest	0.17	99.71	0.01	0.03
709	Male	Youth	Forest	9.51	52.45	8.3	8.21
710	Male	Youth	Forest	7.49	23.71	46.99	12.74
711	Male	Youth	Forest	19.14	32	4.96	17.11
712	Male	Youth	Forest	66.18	7.81	17.63	4.2
713	Male	Youth	Forest	43.63	5.14	32.2	9.16
714	Male	Youth	Forest	2.67	96.71	0.14	0.05
715	Male	Youth	Forest	0	0	0	0
716	Male	Youth	Forest	30.03	13.02	21.33	7.33
717	Male	Youth	Forest	0.61	99.05	0.11	0.07
718	Male	Youth	Forest	11.76	70.07	12.91	3.17
719	Male	Youth	Forest	14.56	67.57	3.13	2.18
720	Male	Youth	Forest	0.35	94.34	0.15	1.65
721	Male	Youth	Forest	5.65	81.13	3.67	0.45
722	Male	Youth	Forest	14.66	51.87	9.01	12.61
723	Male	Youth	Forest	74.09	2.97	15.81	1.41
724	Male	Youth	Forest	32.87	0.27	10.5	15.02
725	Male	Youth	Forest	1.97	30.77	1.58	6.17
726	Male	Youth	Forest	8.04	67.95	1.39	6.91
727	Male	Youth	Forest	1.46	0.17	5.89	21.88
728	Male	Youth	Forest	0.44	98.14	0.83	0.12
729	Male	Youth	Forest	84.4	12.22	1.83	1
730	Male	Youth	Forest	3.37	84.25	6.2	2.87
731	Male	Youth	Forest	8.32	57.8	14.62	9.67
732	Male	Youth	Forest	55.01	23.15	12.39	3.73
733	Male	Youth	Forest	1.01	97.87	0.05	0.86
734	Male	Youth	Forest	9.54	14.44	13.74	11.93
735	Male	Youth	Forest	1.23	98.28	0.09	0.18
736	Male	Youth	Forest	0	0	0	0
737	Male	Youth	Forest	69.03	20.37	4.02	3.86
738	Male	Youth	Forest	22.83	5.34	9.43	34.43
739	Male	Youth	Forest	22.11	51.65	11.83	4.88
740	Male	Youth	Forest	49.51	3.68	9.83	12.14

741	Male	Youth	Forest	0.04	96.01	0.18	2.86
742	Male	Youth	Forest	0.03	99.54	0.01	0.22
743	Male	Youth	Forest	26.11	0.65	14	18.32
744	Male	Youth	Forest	54.3	0.25	17.13	6.12
745	Male	Youth	Forest	39.92	54.88	1.36	2.59
746	Male	Youth	Forest	4.61	92.61	1.82	0.47
747	Male	Youth	Forest	4.41	92.2	0.2	1.86
748	Male	Youth	Forest	1.83	97.71	0.22	0.11
749	Male	Youth	Forest	1.76	96.76	0.73	0.41
750	Male	Youth	Forest	54.05	4.05	2.97	14.47
751	Male	Youth	Forest	22.34	27.9	8.16	4.78
752	Male	Youth	Forest	0	99.87	0.01	0.05
753	Male	Youth	Forest	0.96	98.33	0.2	0.19
754	Male	Youth	Forest	97.16	1.02	0.51	0.28
755	Male	Youth	Forest	0.01	99.52	0.01	0.41
756	Male	Youth	Forest	4.95	39.7	8.19	26.26
757	Male	Youth	Forest	7.32	80.95	2.35	2.89
758	Male	Youth	Forest	42.42	3.49	43.62	3.21
759	Male	Youth	Forest	27.36	46.8	15.98	7.29
760	Male	Youth	Forest	17.01	0.44	33.84	22.1
761	Male	Youth	Forest	2.47	89.37	4.85	1.38
762	Male	Youth	Forest	19.99	27.91	13.25	18.79
763	Male	Youth	Forest	8.59	84.86	2.92	0.94
764	Male	Youth	Forest	6.9	84.4	2.57	2.04
765	Female	Middle	Forest	8.6	0.34	48.99	8.75
766	Female	Middle	Forest	91.08	0.1	3.1	1.41
767	Female	Middle	Forest	34.18	6.22	47.23	7.27
768	Female	Middle	Forest	25.72	0.04	42.99	15.66
769	Female	Middle	Forest	11.03	2.13	23.89	25.87
770	Female	Middle	Forest	13.17	0.08	38.77	7.27
771	Female	Middle	Forest	0.32	1.19	5.6	62.23
772	Female	Middle	Forest	19.61	0.1	65.99	6.96
773	Female	Middle	Forest	36.49	0.87	45.3	11.25
774	Female	Middle	Forest	24.85	1.5	28.2	24.91
775	Female	Middle	Forest	72.01	6.15	8.88	0.34
776	Female	Middle	Forest	41.04	0.9	25.79	8.36
777	Female	Middle	Forest	0	0	0	0
778	Female	Middle	Forest	17.35	5.22	23.13	24.34
779	Female	Middle	Forest	33.92	7.1	29.4	13.72
780	Female	Middle	Forest	14.63	1.43	67.41	5.6
781	Female	Middle	Forest	25.57	1.37	52.35	10.14
782	Female	Middle	Forest	35.2	0.07	38.74	15.5
783	Female	Middle	Forest	8.76	0.82	35.14	35.8
784	Female	Middle	Forest	66.24	0.17	6.6	23.23
785	Female	Middle	Forest	10.59	3.67	59.17	11.43
786	Female	Middle	Forest	11.7	0.42	3.51	58.02
787	Female	Middle	Forest	41.2	0.02	36.42	12.46
788	Female	Middle	Forest	24.88	0.34	24.7	14.26
789	Female	Middle	Forest	60.44	1.97	30.59	2.11
790	Female	Middle	Forest	6.53	0.05	87.04	1.63
791	Female	Middle	Forest	53.88	21.3	7.25	8
792	Female	Middle	Forest	46.09	35.88	8.21	8.04
793	Female	Middle	Forest	17.51	29.01	30.55	11.52
794	Female	Middle	Forest	87.46	0.31	11.44	0.11
795	Female	Middle	Forest	71.52	4.31	5.33	7.74
796	Female	Middle	Forest	28.94	6.43	48.09	7.33
797	Female	Middle	Forest	31.57	5.83	39.03	9.11

798	Female	Middle	Forest	17.79	2.93	36.69	30.02
799	Female	Middle	Forest	63.65	3.89	24.03	3.24
800	Female	Middle	Forest	13.08	52.88	27.97	3.37
801	Female	Middle	Forest	31.68	2.22	47.19	2.89
802	Female	Middle	Forest	76.37	0.66	10.6	5.57
803	Female	Middle	Forest	18.47	2.98	16.18	7.58
804	Female	Middle	Forest	40.61	3.1	35.66	7.96
805	Female	Middle	Forest	26.33	0.78	28.31	18.37
806	Female	Middle	Forest	34.58	0.51	5.71	54.65
807	Female	Middle	Forest	0	0	0	0
808	Female	Middle	Forest	27.41	6.67	28.2	3.66
809	Female	Middle	Forest	77.86	3.84	8.4	4.02
810	Female	Middle	Forest	68.57	8.05	10.22	9.41
811	Female	Middle	Forest	21.15	47.35	4.18	24.36
812	Female	Middle	Forest	35.49	1.42	27.56	8.17
813	Female	Middle	Forest	27.12	1.56	54.51	9.37
814	Female	Middle	Forest	25.85	3.12	21.43	24.42
815	Female	Middle	Forest	7.54	0.01	50.28	28.14
816	Female	Middle	Forest	33.03	25.2	36.04	2.65
817	Female	Middle	Forest	76.78	0.02	16.02	6.13
818	Female	Middle	Forest	12.68	4.39	31.49	9.08
819	Female	Middle	Forest	14.43	4.78	24.62	2.41
820	Female	Middle	Forest	23.73	10.19	19.29	10.29
821	Female	Middle	Forest	57.27	19.38	19.37	1.23
822	Female	Middle	Forest	32.73	19.98	28.54	8.36
823	Female	Middle	Forest	39.26	0.21	5.42	27.68
824	Female	Middle	Forest	1.16	96.48	1.85	0.22
825	Female	Middle	Forest	18.7	28.21	29.73	4.81
826	Female	Middle	Forest	39.14	6.11	46.1	2.23
827	Female	Middle	Forest	78.57	0.82	14.62	1.64
828	Female	Middle	Forest	29.09	2.83	39.79	13.21
829	Female	Middle	Forest	20.38	0.05	70.19	0.98
830	Female	Middle	Forest	43.41	0.2	34.95	12.11
831	Female	Middle	Forest	12.99	43.28	2.94	7.75
832	Female	Middle	Forest	38.3	2.16	20.29	33.14
833	Female	Middle	Forest	47.38	9.53	15.78	11.96
834	Female	Middle	Forest	37.27	0.03	53.31	3.91
835	Female	Middle	Forest	38.78	0.08	30.63	22.18
836	Female	Middle	Forest	21.29	0.11	65.67	3.9
837	Female	Middle	Forest	11.61	1.68	62.46	6.78
838	Female	Middle	Forest	37.55	0.72	38.52	14.98
839	Female	Middle	Forest	0	0	0	0
840	Female	Middle	Forest	31.52	6.26	25.53	25.18
841	Female	Middle	Forest	79.66	0.14	11.59	4.86
842	Female	Middle	Forest	15.43	5.95	35.28	24.51
843	Female	Middle	Forest	0	0	0	0
844	Female	Middle	Forest	62.61	0.25	29.18	3.78
845	Female	Middle	Forest	36.44	2.16	30.16	21.2
846	Female	Middle	Forest	73.71	2.43	9.25	11.07
847	Female	Middle	Forest	16.69	0.06	42.71	5.68
848	Female	Middle	Forest	33.08	0.07	31.54	21.22
849	Female	Middle	Forest	13.17	1.22	6.69	18.39
850	Female	Middle	Forest	0	0	0	0
851	Female	Middle	Forest	33.59	0.07	6.01	25.86
852	Female	Middle	Forest	7.19	0.02	6.61	40.06
853	Female	Middle	Forest	18.07	0.06	18.21	31.76
854	Female	Middle	Forest	6.33	0.23	61.8	15.99

855	Female	Middle	Forest	79.65	0.19	10.9	5.81
856	Female	Middle	Forest	11.07	1.79	45.26	7.12
857	Female	Middle	Forest	13.23	0.28	38.71	16.91
858	Female	Middle	Forest	30	3.9	46.14	8
859	Female	Middle	Forest	23.88	56.21	13.51	1.92
860	Female	Middle	Forest	27.49	5.3	35.17	2.64
861	Female	Middle	Forest	70.27	1.74	12.1	8.55
862	Female	Middle	Forest	25.66	6.02	9.95	19.83
863	Female	Toddler	Forest	20.4	0.21	63.47	8.39
864	Female	Toddler	Forest	58.29	0.13	15.66	20.23
865	Female	Toddler	Forest	31.56	1.11	31.63	18.32
866	Female	Toddler	Forest	27.71	0.05	52.02	10.95
867	Female	Toddler	Forest	44.98	0.31	38.07	11.79
868	Female	Toddler	Forest	52.65	0.7	36.71	7.52
869	Female	Toddler	Forest	63.22	0.15	24.52	9.25
870	Female	Toddler	Forest	43.35	0.06	23.25	29.35
871	Female	Toddler	Forest	38.83	0.41	7.2	24.79
872	Female	Toddler	Forest	55.03	4.18	19.09	5.67
873	Female	Toddler	Forest	38.07	0.79	20.95	36.03
874	Female	Toddler	Forest	71.64	3.51	13.91	1.48
875	Female	Toddler	Forest	29.58	0.18	54.32	6.19
876	Female	Toddler	Forest	33.67	0.85	43.36	6.97
877	Female	Toddler	Forest	63.29	0.06	27.18	6.51
878	Female	Toddler	Forest	0	0	0	0
879	Female	Toddler	Forest	61.15	29.37	4.31	3.68
880	Female	Toddler	Forest	10.99	3.43	47.18	13.4
881	Female	Toddler	Forest	0	0	0	0
882	Female	Toddler	Forest	25.53	1.77	16.36	17.42
883	Female	Toddler	Forest	82.96	3.88	5.87	3.52
884	Female	Toddler	Forest	71.85	0.06	8.5	14.94
885	Female	Toddler	Forest	20.36	0.33	47.87	8.08
886	Female	Toddler	Forest	12.66	0.01	58.39	24.67
887	Female	Toddler	Forest	62.94	1.58	19.13	6.75
888	Female	Toddler	Forest	96.84	0.01	2.1	0.67
889	Female	Toddler	Forest	54.11	1.17	31.04	2.9
890	Female	Toddler	Forest	0	0	0	0
891	Female	Old	Forest	70.43	1.07	14.9	11.65
892	Female	Old	Forest	57.43	3.35	36.57	1.15
893	Female	Old	Forest	6.03	0	34.29	33.84
894	Female	Old	Forest	12.89	0.16	66.7	10.21
895	Female	Old	Forest	0	0	0	0
896	Female	Old	Forest	54.85	16.05	20.51	3.55
897	Female	Old	Forest	26.88	6.87	54.62	6.06
898	Female	Old	Forest	34.5	0.45	13.63	15.08
899	Female	Old	Forest	28.99	5.08	37.65	5.26
900	Female	Old	Forest	16.48	5.78	67.44	2.31
901	Female	Old	Forest	40.38	2.27	50.08	1.23
902	Female	Old	Forest	50.59	35.04	5.36	7.63
903	Female	Old	Forest	66.65	3.7	15.94	5.25
904	Female	Old	Forest	26.75	0.6	54.03	8.63
905	Female	Old	Forest	7.34	0.64	45.47	23.39
906	Female	Old	Forest	0	0	0	0
907	Female	Old	Forest	24.51	2.19	54.37	11.58
908	Female	Old	Forest	2.01	65.6	13.82	14.22
909	Female	Old	Forest	20.32	1.15	52.62	19.02
910	Female	Old	Forest	73.95	5.98	15.09	3.09
911	Female	Old	Forest	18.12	1.98	40.35	12.01

912	Female	Old	Forest	39.52	5.71	38.18	7.16
913	Female	Old	Forest	43.17	1.32	47.02	3.67
914	Female	Old	Forest	4.47	59.5	15.31	8.22
915	Female	Old	Forest	0	0	0	0
916	Female	Old	Forest	1.13	94.97	3.14	0.42
917	Female	Old	Forest	5.16	0.98	53.35	9.26
918	Female	Old	Forest	4.4	12.01	34.07	35.37
919	Female	Old	Forest	0	0	0	0
920	Female	Old	Forest	15.03	0.84	37.98	25.25
921	Female	Old	Forest	10.68	0.03	49.19	24.18
922	Female	Old	Forest	28.06	67.83	1.73	1.37
923	Female	Old	Forest	88.26	0.49	6	1.63
924	Female	Old	Forest	34.58	2.5	49.79	4.32
925	Female	Old	Forest	36.62	0.22	19.15	29.05
926	Female	Old	Forest	36.78	2.94	33.47	18.04
927	Female	Old	Forest	15.27	1.16	52.03	6.33
928	Female	Old	Forest	18.45	0.48	28.76	14.08
929	Female	Old	Forest	12.2	1.14	67.54	8.37
930	Female	Old	Forest	18.23	27.39	40.3	8.13
931	Female	Old	Forest	30.81	0.34	28.33	32.74
932	Female	Old	Forest	35.72	47.45	10.99	1.69
933	Female	Old	Forest	31.5	5.73	20.51	30.34
934	Female	Old	Forest	7.76	7.88	55.44	5.71
935	Female	Old	Forest	6.4	6.3	68.45	5.04
936	Female	Old	Forest	28.03	3	58.59	7.19
937	Female	Old	Forest	42.71	7.79	27.33	1.5
938	Female	Old	Forest	0	0	0	0
939	Female	Old	Forest	17.74	1.79	20.79	28.6
940	Female	Youth	Forest	38.71	0.58	26.91	12.89
941	Female	Youth	Forest	0	0	0	0
942	Female	Youth	Forest	23.63	0.82	55.39	6.93
943	Female	Youth	Forest	8.19	0.2	61.98	5.88
944	Female	Youth	Forest	34.03	0.7	29.74	22.24
945	Female	Youth	Forest	71.96	0.12	17.25	2.73
946	Female	Youth	Forest	14.14	0.26	44.41	20.35
947	Female	Youth	Forest	63.48	7.59	16.88	4.02
948	Female	Youth	Forest	30.61	1.26	17.43	10.27
949	Female	Youth	Forest	25.32	0.09	67.72	3.88
950	Female	Youth	Forest	76.5	2.4	17.43	1.57
951	Female	Youth	Forest	52.94	1.5	32.77	10.01
952	Female	Youth	Forest	40.55	0.43	43	8.71
953	Female	Youth	Forest	35.74	0.2	54.59	3.8
954	Female	Youth	Forest	80.09	0.03	17.2	1.68
955	Female	Youth	Forest	9.15	0.07	47.27	27.93
956	Female	Youth	Forest	84.11	11.17	4.07	0.24
957	Female	Youth	Forest	47.17	0.01	16.03	6.79
958	Female	Youth	Forest	24.08	8.03	20.47	15.37
959	Female	Youth	Forest	16.6	0.31	44.17	6.89
960	Female	Youth	Forest	26.05	0.07	43.79	11.66
961	Female	Youth	Forest	0	0	0	0
962	Female	Youth	Forest	3.33	3.56	86.38	1.54
963	Female	Youth	Forest	69.35	6.31	18.48	3.6
964	Female	Youth	Forest	48.76	1.15	30.2	12.13
965	Female	Youth	Forest	54.2	6.1	21.87	6.51
966	Female	Youth	Forest	77.08	0.73	14.8	3.79
967	Female	Youth	Forest	84.6	0.82	9.64	3
968	Female	Youth	Forest	5.14	67.03	20.17	2.56

969	Female	Youth	Forest	63.87	10.17	16.63	6.49
970	Female	Youth	Forest	13.35	14.74	37.66	10.15
971	Female	Youth	Forest	72.78	0.12	24.66	1.03
972	Female	Youth	Forest	50.44	0.14	14.14	14.22
973	Female	Youth	Forest	41.56	0.07	33.9	6.41
974	Female	Youth	Forest	22	0.4	37.65	31.07
975	Female	Youth	Forest	22.69	3.46	22.21	7.83
976	Female	Youth	Forest	57.9	22.9	9.96	1.65
977	Female	Youth	Forest	23.76	0.88	10.45	16.5
978	Female	Youth	Forest	26.3	0.66	14.39	8.63
979	Female	Youth	Forest	2.87	28.83	3.96	21.2
980	Female	Youth	Forest	13.77	7.45	32.37	15.72
981	Female	Youth	Forest	20.63	0.13	64.71	7.88
982	Female	Youth	Forest	69.21	0.17	16.15	10.83
983	Female	Youth	Forest	87.55	0.5	6.7	1.66
984	Female	Youth	Forest	41.77	3.81	13.73	0.82
985	Female	Youth	Forest	60.87	3.02	19.43	3.16
986	Female	Youth	Forest	77.83	0.31	7.39	11.4
987	Female	Youth	Forest	17.62	0.01	17.31	5.65
988	Female	Youth	Forest	81.39	1.21	8.69	3.86
989	Female	Youth	Forest	17.75	0.23	9.11	18.09
990	Female	Youth	Forest	40.1	1.46	18.59	8.08
991	Female	Youth	Forest	9.97	0.08	59.56	11.08
992	Female	Youth	Forest	39.32	0.02	48.7	7.61
993	Female	Youth	Forest	72.28	2.33	12.84	3.23
994	Female	Youth	Forest	7.57	25.18	57.75	0.7
995	Female	Youth	Forest	8.28	1.38	26.09	27.4
996	Female	Youth	Forest	79.63	0.63	14.52	1.44
997	Female	Youth	Forest	78.65	0.23	18.28	0.65
998	Female	Youth	Forest	45.72	0.36	42.72	6.72
999	Female	Youth	Forest	44.5	0.07	32.6	8.24
1000	Female	Youth	Forest	0	0	0	0
1001	Female	Youth	Forest	38.94	4.17	31.12	8.86
1002	Female	Youth	Forest	18.01	0.63	70.95	1.15
1003	Female	Youth	Forest	35.78	0.01	36.16	7.93
1004	Female	Youth	Forest	79.96	0.17	13.34	4.46
1005	Female	Youth	Forest	30.09	0.07	4.34	3.68
1006	Female	Youth	Forest	12.22	0.5	37.34	9.24
1007	Female	Youth	Forest	9.5	0.07	8.2	1.6
1008	Female	Youth	Forest	26.46	0.19	62.55	1.51
1009	Female	Youth	Forest	15.12	0.98	38.2	20.31
1010	Female	Youth	Forest	63.81	0.09	11.51	17.04
1011	Female	Youth	Forest	0	0	0	0
1012	Female	Youth	Forest	54.08	1.93	30.07	6.58
1013	Female	Youth	Forest	73.02	1.9	17.45	1.42
1014	Female	Youth	Forest	22.52	0.5	56.49	8.78
1015	Female	Youth	Forest	33.44	1.27	42.25	8.1
1016	Female	Youth	Forest	65.84	0.08	19.58	11.33
1017	Female	Youth	Forest	22.98	32.17	3.43	23.05
1018	Female	Youth	Forest	25.62	24.24	18.69	4.22
1019	Female	Youth	Forest	56.9	2.3	14.51	10.6
1020	Female	Youth	Forest	70.77	0.02	17.86	7.39
1021	Female	Youth	Forest	78.69	0.13	10.93	1.86
1022	Female	Youth	Forest	36.25	0.18	21.03	24.8
1023	Female	Youth	Forest	52.92	8.05	17.69	13.1
1024	Female	Youth	Forest	2.76	14.23	1.34	49.42
1025	Female	Youth	Forest	9.38	48.29	16.15	10.33

1026	Female	Youth	Forest	66.67	13.4	5.05	6.56
1027	Female	Youth	Forest	64.72	26.57	4.81	2
1028	Female	Youth	Forest	24.14	0.06	46.99	9.87
1029	Female	Youth	Forest	45.28	0.14	38.33	5.54
1030	Female	Youth	Forest	27.21	3.13	18.33	5.9
1031	Male	Middle	Forest	7.44	0.07	78.44	3.75
1032	Male	Middle	Forest	51.89	0.27	34.73	7.46
1033	Male	Middle	Forest	57.29	6.98	23.68	5.21
1034	Male	Middle	Forest	63.93	10.81	4.89	4.16
1035	Male	Middle	Forest	0	0	0	0
1036	Male	Middle	Forest	46.25	0.98	46.43	2.03
1037	Male	Middle	Forest	9.94	0.03	55.68	8.34
1038	Male	Middle	Forest	3.03	2.56	84.12	4.2
1039	Male	Middle	Forest	4.5	2.38	70.7	5.67
1040	Male	Middle	Forest	36.94	5.73	27.15	19.58
1041	Male	Middle	Forest	0	0	0	0
1042	Male	Middle	Forest	61.83	1.72	26.22	5.83
1043	Male	Middle	Forest	28.69	59.76	3.79	4.51
1044	Male	Middle	Forest	0	0	0	0
1045	Male	Middle	Forest	26.47	0.7	10.45	33.72
1046	Male	Middle	Forest	58.93	0.36	8.96	18.17
1047	Male	Middle	Forest	67.16	0.62	24.78	3.17
1048	Male	Middle	Forest	7.95	27.9	28.22	17.96
1049	Male	Middle	Forest	49.83	2.18	27.37	13.3
1050	Male	Middle	Forest	0.68	3.09	1.45	65.39
1051	Male	Middle	Forest	66.74	8.32	15.6	7.41
1052	Male	Middle	Forest	11.56	0.54	68.91	5.69
1053	Male	Middle	Forest	63.49	0.86	30.04	2.61
1054	Male	Middle	Forest	26.37	0.21	41.4	26.09
1055	Male	Middle	Forest	34.67	33.44	24.33	5.04
1056	Male	Middle	Forest	11.61	0.1	37.19	33.17
1057	Male	Middle	Forest	0.67	0.05	34.87	4.77
1058	Male	Middle	Forest	8.48	18.8	5.66	52.89
1059	Male	Middle	Forest	24.75	0.11	27.99	30.69
1060	Male	Middle	Forest	4.63	0.04	84.4	5.5
1061	Male	Middle	Forest	65.51	1.19	9.01	3.65
1062	Male	Middle	Forest	65.21	0.36	13.33	4.4
1063	Male	Middle	Forest	84.84	2	7.18	2.14
1064	Male	Middle	Forest	46.74	0.03	25.11	23.14
1065	Male	Middle	Forest	40.2	10.79	16.35	19.49
1066	Male	Middle	Forest	43.62	8.06	11.45	30.2
1067	Male	Middle	Forest	23.95	11.43	2.68	11.6
1068	Male	Middle	Forest	9.21	1.44	48.56	13.84
1069	Male	Middle	Forest	65.92	1.26	10.83	18.76
1070	Male	Middle	Forest	36.62	0.53	46.55	13.01
1071	Male	Middle	Forest	17.3	4.53	58.85	7.19
1072	Male	Middle	Forest	33.74	0.11	30.4	24.99
1073	Male	Middle	Forest	0	0	0	0
1074	Male	Middle	Forest	80.37	3	7	7.39
1075	Male	Middle	Forest	61.71	0.3	11.67	4.98
1076	Male	Middle	Forest	5.69	0.6	29.33	17.07
1077	Male	Middle	Forest	70.05	6.71	6.56	6.68
1078	Male	Middle	Forest	38.71	0.12	10.16	10.71
1079	Male	Middle	Forest	24.47	1.16	27.82	17.2
1080	Male	Middle	Forest	42.27	8.48	26.75	14.64
1081	Male	Middle	Forest	75.28	0.08	14.03	4.7
1082	Male	Middle	Forest	11.68	0.64	15.38	24.45

1083	Male	Toddler	Forest	0	0	0	0
1084	Male	Toddler	Forest	42.08	0.17	41.54	3.61
1085	Male	Toddler	Forest	47.11	0.32	34.85	3.64
1086	Male	Toddler	Forest	66.14	0.31	25.76	4.23
1087	Male	Toddler	Forest	38.17	9.81	28.3	12.01
1088	Male	Toddler	Forest	0	0	0	0
1089	Male	Toddler	Forest	22.1	0.13	53.76	7.23
1090	Male	Toddler	Forest	0	0	0	0
1091	Male	Toddler	Forest	15.7	0.01	52.63	26.48
1092	Male	Toddler	Forest	18.65	1.1	13.56	59.58
1093	Male	Toddler	Forest	61.74	0.38	23.24	7.8
1094	Male	Toddler	Forest	22.96	0.36	40.46	23.25
1095	Male	Toddler	Forest	0	0	0	0
1096	Male	Toddler	Forest	0	0	0	0
1097	Male	Toddler	Forest	0	0	0	0
1098	Male	Toddler	Forest	53.96	1.39	24.99	4.39
1099	Male	Toddler	Forest	0	0	0	0
1100	Male	Toddler	Forest	56.9	1.55	7.4	9.56
1101	Male	Toddler	Forest	61.88	0.33	29.77	5.5
1102	Male	Toddler	Forest	5.25	80.51	11.69	0.97
1103	Male	Toddler	Forest	91.37	0.17	5.31	1.76
1104	Male	Toddler	Forest	65.06	0.53	18.18	10.74
1105	Male	Toddler	Forest	57.28	5.17	14.15	5.67
1106	Male	Toddler	Forest	97.64	0.18	1.22	0.32
1107	Male	Toddler	Forest	0	0	0	0
1108	Male	Toddler	Forest	90.34	0.07	6.58	2.13
1109	Male	Toddler	Forest	31.56	0.29	6.97	42.53
1110	Male	Toddler	Forest	74.48	0.59	15.27	5.42
1111	Male	Toddler	Forest	87.78	1.81	4.61	1.98
1112	Male	Old	Forest	48.04	3.64	25.36	7.87
1113	Male	Old	Forest	24.89	6.37	52.17	11.41
1114	Male	Old	Forest	34.67	0.84	38.09	15.23
1115	Male	Old	Forest	21.68	16.62	13.21	15.97
1116	Male	Old	Forest	9.78	0.19	68.13	4.09
1117	Male	Old	Forest	64.51	5.9	23.12	5.87
1118	Male	Old	Forest	60.68	0.26	16.86	15.42
1119	Male	Old	Forest	0	0	0	0
1120	Male	Old	Forest	8.12	72.17	14.52	2.73
1121	Male	Old	Forest	13.03	80.5	3.86	1.18
1122	Male	Old	Forest	11.04	11.73	7.73	53.66
1123	Male	Old	Forest	31.75	0.85	47.69	10.13
1124	Male	Old	Forest	46.66	0.48	19.86	14.46
1125	Male	Old	Forest	18.67	1.67	64.35	6.13
1126	Male	Old	Forest	56.32	1.91	23.23	13.13
1127	Male	Old	Forest	1.01	6.02	27.72	29.42
1128	Male	Old	Forest	8.39	0.59	68.54	8.26
1129	Male	Old	Forest	50.49	1.56	25.1	15.32
1130	Male	Old	Forest	4.82	94.2	0.69	0.16
1131	Male	Old	Forest	27.8	4.41	25.71	27.33
1132	Male	Old	Forest	0	0	0	0
1133	Male	Old	Forest	24.82	3.29	33.47	29.18
1134	Male	Old	Forest	0	0	0	0
1135	Male	Old	Forest	12.7	76.6	9.37	0.85
1136	Male	Old	Forest	0	0	0	0
1137	Male	Old	Forest	0	0	0	0
1138	Male	Old	Forest	35.75	1.66	30.17	6.24
1139	Male	Old	Forest	0	0	0	0

1140	Male	Old	Forest	5.92	0.26	63.5	3.48
1141	Male	Old	Forest	36.81	7.05	20.57	26.5
1142	Male	Old	Forest	10.62	1.44	38.77	7.94
1143	Male	Old	Forest	90.34	1.19	4.47	2.33
1144	Male	Old	Forest	0	0	0	0
1145	Male	Old	Forest	43.31	1.31	27.8	20.37
1146	Male	Old	Forest	0	0	0	0
1147	Male	Old	Forest	5.63	1.6	72.73	8.93
1148	Male	Old	Forest	6.11	5.16	28.06	25.55
1149	Male	Old	Forest	11.91	2.17	71.98	6.65
1150	Male	Old	Forest	3.54	0.4	49.19	19.34
1151	Male	Old	Forest	0	0	0	0
1152	Male	Old	Forest	28.08	49.44	13.11	3.72
1153	Male	Old	Forest	19.82	2.61	49.26	3.41
1154	Male	Old	Forest	0	0	0	0
1155	Male	Old	Forest	3.22	5.89	79.63	1.89
1156	Male	Old	Forest	26.41	0.69	40.65	4.67
1157	Male	Old	Forest	13.8	17.32	42.91	10.44
1158	Male	Old	Forest	0	0	0	0
1159	Male	Old	Forest	14.63	0.86	51.49	21.49
1160	Male	Old	Forest	23.42	1.04	29.39	32.26
1161	Male	Youth	Forest	18.17	0.75	12.72	14.43
1162	Male	Youth	Forest	42.16	46.89	5.91	2.47
1163	Male	Youth	Forest	76.43	7.35	12.72	1.21
1164	Male	Youth	Forest	38.19	3.52	21.38	8.24
1165	Male	Youth	Forest	24.13	1.44	45.35	8.66
1166	Male	Youth	Forest	80.04	4.17	6.62	3.97
1167	Male	Youth	Forest	91.29	0.09	5.76	1.73
1168	Male	Youth	Forest	47.4	0.5	21.39	3.06
1169	Male	Youth	Forest	78.29	1.29	8.03	5.41
1170	Male	Youth	Forest	16.88	1.99	49.68	21.25
1171	Male	Youth	Forest	23.06	1.25	41.2	6.77
1172	Male	Youth	Forest	50.4	2.7	32.5	9.43
1173	Male	Youth	Forest	23.66	2.25	29.68	25.4
1174	Male	Youth	Forest	78.75	0.31	4.15	8.47
1175	Male	Youth	Forest	0	0	0	0
1176	Male	Youth	Forest	82.45	0.65	12.28	1.16
1177	Male	Youth	Forest	58.87	12.89	18.17	6.5
1178	Male	Youth	Forest	61.53	10.93	15.76	7.38
1179	Male	Youth	Forest	47.23	0.1	32.09	6.97
1180	Male	Youth	Forest	4.65	0.28	45.64	8.46
1181	Male	Youth	Forest	0	0	0	0
1182	Male	Youth	Forest	52.65	0.31	13.01	21.29
1183	Male	Youth	Forest	22.68	0.13	16.24	31.7
1184	Male	Youth	Forest	41.77	0.46	33.35	8.91
1185	Male	Youth	Forest	0	0	0	0
1186	Male	Youth	Forest	4.42	0.14	50.79	24.67
1187	Male	Youth	Forest	58.89	0.01	23.36	10.36
1188	Male	Youth	Forest	75.65	0.4	19.11	2.71
1189	Male	Youth	Forest	23.84	25.81	1.92	11.68
1190	Male	Youth	Forest	27.1	0.09	33.73	29.81
1191	Male	Youth	Forest	41.2	3.74	35.82	8.91
1192	Male	Youth	Forest	0	0	0	0
1193	Male	Youth	Forest	76.43	2.1	16.4	1.87
1194	Male	Youth	Forest	3.11	0.11	66.02	9.58
1195	Male	Youth	Forest	31.67	2.55	14.83	20.11
1196	Male	Youth	Forest	14.63	1.78	6.35	19.33

1197	Male	Youth	Forest	84.29	0.43	7.11	2.52
1198	Male	Youth	Forest	65.29	0.82	23.08	6.15
1199	Male	Youth	Forest	10.78	0.05	22.65	57.37
1200	Male	Youth	Forest	6.42	0.46	14.47	27.7
1201	Male	Youth	Forest	28.41	8.58	53.41	6.47
1202	Male	Youth	Forest	37.02	0.3	33.58	15.9
1203	Male	Youth	Forest	7.96	2.06	19.9	46.57
1204	Male	Youth	Forest	65.18	1.74	14.69	10.41
1205	Male	Youth	Forest	24	13.02	30.48	16.67
1206	Male	Youth	Forest	0	0	0	0
1207	Male	Youth	Forest	0	0	0	0
1208	Male	Youth	Forest	0	0	0	0
1209	Male	Youth	Forest	24.79	0.23	28.44	16.27
1210	Male	Youth	Forest	60.07	14.37	10.65	8.21
1211	Male	Youth	Forest	51.84	0.63	25.39	11.51
1212	Male	Youth	Forest	9.66	0.06	20.61	60.54
1213	Male	Youth	Forest	47.56	0.01	29.65	17.02
1214	Male	Youth	Forest	41.09	4.77	30.67	17.75
1215	Male	Youth	Forest	24.74	2.41	43.17	14.74
1216	Male	Youth	Forest	8.15	0.09	78.76	4.79
1217	Male	Youth	Forest	25.7	0.16	44.03	5.99
1218	Male	Youth	Forest	59.99	1.41	7.84	6.42
1219	Male	Youth	Forest	71.67	0.08	8.07	5.19
1220	Male	Youth	Forest	72.4	0.65	15.98	7.55
1221	Male	Youth	Forest	25.3	4.01	13.76	20.29
1222	Male	Youth	Forest	39.29	0.52	25.56	21.36
1223	Male	Youth	Forest	21.35	0.48	24.98	9.42
1224	Male	Youth	Forest	44.05	4.88	19.09	16.33
1225	Male	Youth	Forest	64.27	0.68	14.27	18.77
1226	Male	Youth	Forest	56.61	0.36	20.01	9.93
1227	Male	Youth	Forest	69.46	0.6	9.62	6.23
1228	Male	Youth	Forest	74.76	2.07	9.43	5.5
1229	Male	Youth	Forest	11.7	0.26	44.07	34.97
1230	Male	Youth	Forest	19.27	0.09	20.93	37.28
1231	Male	Youth	Forest	27.28	0.71	29.6	27.07
1232	Male	Youth	Forest	6.97	0.96	9.46	14.25
1233	Male	Youth	Forest	18.35	0.08	23.57	21.08
1234	Male	Youth	Forest	5.33	54.03	9.6	9.02
1235	Male	Youth	Forest	55.58	0.05	20.98	10.49
1236	Male	Youth	Forest	22.87	3.46	5.81	15.71
1237	Male	Youth	Forest	27.87	0.53	17.49	24.45
1238	Male	Youth	Forest	4.26	0.04	65.82	12.13
1239	Male	Youth	Forest	49.7	0.08	12.18	20.64
1240	Male	Youth	Forest	17.77	5.07	27.54	19.18
1241	Male	Youth	Forest	8.78	0.11	28.52	38.23
1242	Male	Youth	Forest	56.37	0.31	13.31	11.14
1243	Male	Youth	Forest	0	0	0	0
1244	Male	Youth	Forest	44.06	1.69	21.17	18.41
1245	Male	Youth	Forest	44.76	0.51	17.45	16.81
1246	Male	Youth	Forest	90.15	0.09	3.31	5.6
1247	Male	Youth	Forest	30.26	0.08	9.53	20.92
1248	Male	Youth	Forest	0	0	0	0
1249	Male	Youth	Forest	65.64	3.65	20.34	5.05
1250	Male	Youth	Forest	30.18	1.57	21.14	28.53
1251	Male	Youth	Forest	72.08	0.08	11.2	4.39
1252	Male	Youth	Forest	36.1	0.13	32.17	10.72
1253	Male	Youth	Forest	16.02	0.07	55.42	8.84

1254	Male	Youth	Forest	83.36	14.26	1	0.78
1255	Male	Youth	Forest	39.68	0.12	26.13	16.51
1256	Male	Youth	Forest	43.1	1.11	7.99	30.66
1257	Male	Youth	Forest	40.57	0.1	11.11	27.58
1258	Male	Youth	Forest	7.67	0.01	10.15	17.74
1259	Male	Youth	Forest	35.06	2.43	19.77	17.25
1260	Male	Youth	Forest	46.49	0.93	15.58	12.34
1261	Male	Youth	Forest	12.42	0.31	16.1	9.76
1262	Male	Youth	Forest	12.14	0.03	39.15	12.75
1263	Male	Youth	Forest	71.95	4.77	6.84	13.81
1264	Male	Youth	Forest	62.14	0.37	18.88	6.86
1265	Male	Youth	Forest	5.93	0.86	85.42	1.2
1266	Male	Youth	Forest	24.22	0.46	19.24	20.08
1267	Male	Youth	Forest	8.79	3.66	33.71	6.68
1268	Male	Youth	Forest	0	0	0	0
1269	Male	Youth	Forest	50.32	8.81	20.98	3.65
1270	Male	Youth	Forest	22.88	0.08	42.83	13.09
1271	Female	Middle	Forest	60.57	0.45	19.76	14.55
1272	Female	Middle	Forest	26.9	0.32	10.42	7.07
1273	Female	Middle	Forest	3.29	0	74.94	16.56
1274	Female	Middle	Forest	60.8	0.59	16.59	8.93
1275	Female	Middle	Forest	25.57	1.37	52.35	10.14
1276	Female	Middle	Forest	6.34	0.14	49.75	22.4
1277	Female	Middle	Forest	12.57	0.03	45.97	29.1
1278	Female	Middle	Forest	23.76	0.44	46.76	13.7
1279	Female	Middle	Forest	33.97	20.92	13.67	26.84
1280	Female	Middle	Forest	0	0	0	0
1281	Female	Middle	Forest	67.95	0.01	19.72	3.76
1282	Female	Middle	Forest	10.96	0.19	67.72	13.1
1283	Female	Middle	Forest	15.7	0.32	57.92	7.88
1284	Female	Middle	Forest	43.71	0.17	45.73	2.51
1285	Female	Middle	Forest	47.74	0.24	37.82	6.02
1286	Female	Middle	Forest	6.26	0.05	15.11	56.77
1287	Female	Middle	Forest	2.9	0.18	40.88	2.85
1288	Female	Middle	Forest	5.14	0.08	2.91	3.19
1289	Female	Middle	Forest	49.55	2.61	32.77	7.25
1290	Female	Middle	Forest	27.47	1.56	30.81	12.92
1291	Female	Middle	Forest	19.99	0.24	48.54	10.23
1292	Female	Middle	Forest	24.45	0.16	47.46	18.96
1293	Female	Middle	Forest	7.36	2.52	44.05	12.46
1294	Female	Middle	Forest	17.69	0.41	65.87	11.79
1295	Female	Middle	Forest	6.89	0.01	55.57	26.67
1296	Female	Middle	Forest	8.21	0.86	41.22	12.91
1297	Female	Middle	Forest	18.8	15.67	14.23	17.22
1298	Female	Toddler	Forest	2.27	0.23	68.76	6.03
1299	Female	Toddler	Forest	54.81	0.1	27.54	8.58
1300	Female	Toddler	Forest	12.15	39.2	8.57	20.14
1301	Female	Toddler	Forest	55.82	0.07	31.73	8.45
1302	Female	Toddler	Forest	53.12	0.16	21.27	17.34
1303	Female	Toddler	Forest	41.65	0.01	37.12	13.55
1304	Female	Old	Forest	0	0	0	0
1305	Female	Old	Forest	13.84	0.04	45.01	31.2
1306	Female	Old	Forest	2.04	0.03	22.32	7.18
1307	Female	Old	Forest	19.51	2.45	23.35	8.1
1308	Female	Old	Forest	6.29	0.39	54.76	10.22
1309	Female	Old	Forest	14.34	31.49	25.99	7.17
1310	Female	Old	Forest	47.85	6.29	38.85	2.94

1311	Female	Old	Forest	26.21	3.93	21.87	37.56
1312	Female	Old	Forest	0	0	0	0
1313	Female	Old	Forest	30.64	5.06	57.71	2.43
1314	Female	Old	Forest	18.16	7.3	69.01	1.79
1315	Female	Old	Forest	0	0	0	0
1316	Female	Old	Forest	34.17	0.2	57.79	2.95
1317	Female	Old	Forest	30.95	0.96	34.61	17.78
1318	Female	Old	Forest	19.73	8.56	41.42	14.52
1319	Female	Old	Forest	5.02	0.14	46.1	38.31
1320	Female	Old	Forest	33.34	2.12	59.23	2.71
1321	Female	Old	Forest	9.18	6.67	58.04	2.68
1322	Female	Old	Forest	18.31	0.04	76.84	2.29
1323	Female	Old	Forest	38.77	0.87	25.49	8.19
1324	Female	Old	Forest	40.14	2.75	46.99	5.28
1325	Female	Old	Forest	13.89	0.89	72.57	5.12
1326	Female	Old	Forest	4.22	7.01	71.44	7.39
1327	Female	Old	Forest	36.38	6.25	25.55	17.66
1328	Female	Old	Forest	36.97	47.34	8.03	2.05
1329	Female	Old	Forest	6.89	0.13	35.57	7.29
1330	Female	Old	Forest	57.49	5.68	32.94	1.47
1331	Female	Old	Forest	41.43	0.71	46.05	5.18
1332	Female	Old	Forest	1.38	0.33	94.53	0.8
1333	Female	Old	Forest	0	0	0	0
1334	Female	Old	Forest	30.35	0.06	20.77	38.83
1335	Female	Old	Forest	14.79	5.45	48.93	9.18
1336	Female	Old	Forest	22.79	0.57	68.43	3.1
1337	Female	Old	Forest	4.92	1.56	77.34	1.88
1338	Female	Old	Forest	25.11	0.44	61.29	5.87
1339	Female	Old	Forest	25.06	0.71	23.23	41.16
1340	Female	Old	Forest	30.83	0.95	50.12	11.33
1341	Female	Youth	Forest	45.84	0.02	42.82	4.41
1342	Female	Youth	Forest	13.23	0.64	55.71	3.41
1343	Female	Youth	Forest	27.55	0.25	49.23	8.92
1344	Female	Youth	Forest	17.52	18.28	12.3	28.96
1345	Female	Youth	Forest	17.34	7.57	51.84	8.91
1346	Female	Youth	Forest	54.81	0.14	30.2	5.54
1347	Female	Youth	Forest	36.66	0.06	23.74	25.2
1348	Female	Youth	Forest	65.93	0.24	18.37	6.82
1349	Female	Youth	Forest	58.47	0.04	10.5	21
1350	Female	Youth	Forest	52.04	0.73	19.06	9.48
1351	Female	Youth	Forest	9.64	0.02	23.63	14.28
1352	Female	Youth	Forest	2.99	0.12	65.04	0.92
1353	Female	Youth	Forest	32.99	0.1	16.08	32.48
1354	Female	Youth	Forest	41.79	0.18	31.62	14.16
1355	Female	Youth	Forest	30.63	0.03	50.56	5.02
1356	Male	Middle	Forest	14.35	4.73	47.29	15.29
1357	Male	Middle	Forest	40.39	1.97	31.37	15.37
1358	Male	Middle	Forest	17.67	1.55	60.55	5.17
1359	Male	Middle	Forest	29.5	0.04	47.09	14.23
1360	Male	Middle	Forest	36.45	1.72	35.99	21.24
1361	Male	Middle	Forest	4.41	0.22	21.26	62.62
1362	Male	Middle	Forest	23.03	0.42	59.01	8.97
1363	Male	Middle	Forest	66.75	2.63	6.7	21.02
1364	Male	Middle	Forest	33.81	0.4	33.11	23.18
1365	Male	Middle	Forest	17.38	2.63	65.2	8.77
1366	Male	Middle	Forest	28.62	6.76	42.09	13.55
1367	Male	Middle	Forest	39.17	0.29	46.52	8.81

1368	Male	Middle	Forest	40.67	0.94	17.28	31.31
1369	Male	Middle	Forest	37.63	0.57	41.66	12.87
1370	Male	Middle	Forest	17.55	0.82	54.22	14.33
1371	Male	Middle	Forest	0	0	0	0
1372	Male	Middle	Forest	32.24	19.7	20.21	7.72
1373	Male	Middle	Forest	72.04	0.35	7.19	4.26
1374	Male	Middle	Forest	4.56	0.08	81.56	3.25
1375	Male	Middle	Forest	16.38	0.16	43.94	18.75
1376	Male	Middle	Forest	4.09	0.23	11.92	32.18
1377	Male	Middle	Forest	7.53	1.64	52.52	8.17
1378	Male	Middle	Forest	43.92	2.65	26.7	13.77
1379	Male	Toddler	Forest	35.56	0.02	24.41	28.01
1380	Male	Toddler	Forest	46.59	1.82	13.31	17.94
1381	Male	Old	Forest	4.21	45.18	38.35	6.89
1382	Male	Old	Forest	48.78	1.29	32.03	11.85
1383	Male	Old	Forest	0	0	0	0
1384	Male	Old	Forest	0	0	0	0
1385	Male	Old	Forest	28.75	1.66	15.1	49.3
1386	Male	Old	Forest	27.26	0.82	53.77	8.17
1387	Male	Old	Forest	37.17	47.4	11.76	2.5
1388	Male	Old	Forest	2.17	38.13	9.31	20.79
1389	Male	Old	Forest	0	0	0	0
1390	Male	Old	Forest	45.75	23.14	11.46	12.44
1391	Male	Old	Forest	0	0	0	0
1392	Male	Old	Forest	4.26	76.63	7.52	6.4
1393	Male	Old	Forest	11.57	0.3	22.79	18.54
1394	Male	Old	Forest	10.19	1.33	56.77	12.53
1395	Male	Old	Forest	10.58	0.19	67.06	3.47
1396	Male	Old	Forest	8.67	0	75.44	7.06
1397	Male	Old	Forest	7.77	0.56	68.19	5.66
1398	Male	Old	Forest	0	0	0	0
1399	Male	Old	Forest	8.41	0.38	58.38	14.24
1400	Male	Old	Forest	33.55	2.62	41.4	16.86
1401	Male	Old	Forest	10.46	0.12	60.34	16.19
1402	Male	Old	Forest	32.93	3.25	41.62	11.15
1403	Male	Old	Forest	10.16	0.4	39.63	25.33
1404	Male	Old	Forest	27	8.36	37.98	8.73
1405	Male	Old	Forest	12.42	0.09	34.59	36.79
1406	Male	Old	Forest	0	0	0	0
1407	Male	Old	Forest	52.14	0	29.73	15.8
1408	Male	Old	Forest	6.29	1.43	53.54	26.52
1409	Male	Old	Forest	5.52	1.98	75.62	5.6
1410	Male	Old	Forest	14.51	0.42	54.92	21.94
1411	Male	Old	Forest	0	0	0	0
1412	Male	Old	Forest	27.88	0.22	59.88	3.58
1413	Male	Youth	Forest	6.66	0.01	73.03	5.87
1414	Male	Youth	Forest	89.2	5.1	2.47	0.68
1415	Male	Youth	Forest	8.44	0.03	16.36	44.01
1416	Male	Youth	Forest	13.09	1.01	50.68	16.48
1417	Male	Youth	Forest	22.61	0.69	35.06	21.73
1418	Male	Youth	Forest	35.73	0.18	43.35	7.54
1419	Male	Youth	Forest	36.32	0.08	33.71	18.72
1420	Male	Youth	Forest	31.06	0.22	21.05	12.34
1421	Male	Youth	Forest	5.12	0.05	25.48	20.07
1422	Male	Youth	Forest	0	0	0	0
1423	Male	Youth	Forest	23.1	1.32	49.33	11.72
1424	Female	Middle	Forest	0	0	0	0

1425	Female	Middle	Forest	17.4	3.06	63.17	5.17
1426	Female	Middle	Forest	0	0	0	0
1427	Female	Middle	Forest	0	0	0	0
1428	Female	Middle	Forest	8.64	0.22	53.05	11.84
1429	Female	Middle	Forest	64.89	5.85	22.67	1.62
1430	Female	Toddler	Forest	34.44	5.34	37.42	11.74
1431	Female	Toddler	Forest	50.28	0.21	30.74	8.88
1432	Female	Toddler	Forest	56.66	3.79	22.71	4.93
1433	Female	Toddler	Forest	58.42	0.42	11.08	0.99
1434	Female	Old	Forest	34.06	45.83	11.58	5.78
1435	Female	Youth	Forest	42.41	0.11	15.45	5.3
1436	Female	Youth	Forest	0	0	0	0
1437	Female	Youth	Forest	65.17	1.01	8.22	3.87
1438	Female	Youth	Forest	29.98	0.51	15.21	12.91
1439	Male	Middle	Forest	11.98	2.05	32.83	25.61
1440	Male	Middle	Forest	30.81	1	25.36	19.19
1441	Male	Middle	Forest	77.73	1.34	12.49	5.57
1442	Male	Middle	Forest	4.37	85.36	2.61	0.97
1443	Male	Middle	Forest	35.43	0.05	50.72	8.04
1444	Male	Toddler	Forest	2.27	2.6	9.84	27.35
1445	Male	Toddler	Forest	22.1	6.93	48.68	12.32
1446	Male	Toddler	Forest	46.47	0.02	27.37	4.86
1447	Male	Toddler	Forest	30.55	0.46	17.02	12.06
1448	Male	Toddler	Forest	7	0.16	6.34	27.9
1449	Male	Old	Forest	7.03	1.16	10.02	11.27
1450	Male	Old	Forest	3.82	0.04	89.46	1.56
1451	Male	Old	Forest	10.46	0.92	20.7	10.81
1452	Male	Old	Forest	13.18	0.78	23.23	10.69
1453	Male	Old	Forest	7.97	0.2	47.82	11.26
1454	Male	Old	Forest	26.65	6.19	18.2	39.17
1455	Male	Youth	Forest	39.63	0.44	41.55	7.23
1456	Male	Youth	Forest	18.27	0.01	33.15	12.25
1457	Male	Youth	Forest	2.59	0.76	10.18	14.3
1458	Female	Middle	Forest	22.9	35.42	35.65	1.98
1459	Female	Middle	Forest	11.24	1.36	53.42	11.18
1460	Female	Middle	Forest	18.34	4.08	65.43	4.21
1461	Female	Middle	Forest	17.78	0.03	61.51	8.22
1462	Female	Middle	Forest	88.31	3.1	4.81	0.35
1463	Female	Middle	Forest	9.24	1.24	51.22	13.44
1464	Female	Middle	Forest	0	0	0	0
1465	Female	Middle	Forest	57.55	0.05	31.19	5.03
1466	Female	Middle	Forest	0	0	0	0
1467	Female	Middle	Forest	90.58	0.47	4.56	1.9
1468	Female	Middle	Forest	34.68	0.3	26.28	15.91
1469	Female	Middle	Forest	13.71	0.2	19.78	15.7
1470	Female	Middle	Forest	3.17	0.64	49.93	4.63
1471	Female	Middle	Forest	11	4.98	5.96	12.82
1472	Female	Toddler	Forest	49.48	0.03	41.83	2.96
1473	Female	Toddler	Forest	90.44	1.59	2.83	1.16
1474	Female	Toddler	Forest	43.79	2.27	7.42	23.95
1475	Female	Toddler	Forest	19.47	73.8	1.66	3.2
1476	Female	Toddler	Forest	87.3	0.03	8.35	2.49
1477	Female	Toddler	Forest	83.28	6.03	6.96	2.75
1478	Female	Old	Forest	25.8	4.1	34.6	28.5
1479	Female	Old	Forest	14.34	11.24	68.83	1.29
1480	Female	Old	Forest	17.8	1.88	24.31	12.7
1481	Female	Old	Forest	7.95	2.82	68.35	5.68

1482	Female	Old	Forest	6.73	0.43	51.01	6.79
1483	Female	Old	Forest	7.54	1.05	44.39	11.98
1484	Female	Old	Forest	57.49	23.57	5.59	3.51
1485	Female	Youth	Forest	10.92	0.86	40.7	12.54
1486	Female	Youth	Forest	27.4	0.7	34.66	5.68
1487	Female	Youth	Forest	32.21	0.48	7.24	2.82
1488	Female	Youth	Forest	88.5	0.31	6.81	2.4
1489	Female	Youth	Forest	64.38	0.24	18.72	2.58
1490	Female	Youth	Forest	25.67	14.43	29.7	9.32
1491	Female	Youth	Forest	62.9	0.04	13.33	12.18
1492	Female	Youth	Forest	9.91	0.35	19.65	7.89
1493	Female	Youth	Forest	4.05	60.1	19	4.1
1494	Female	Youth	Forest	45.79	0.21	41.69	6.08
1495	Female	Youth	Forest	65.91	0.03	11.75	4.35
1496	Female	Youth	Forest	40.1	1.46	18.59	8.08
1497	Female	Youth	Forest	19.59	21.65	31.43	10.91
1498	Female	Youth	Forest	24.94	0.45	30.47	1.16
1499	Male	Middle	Forest	16.3	0.41	21.25	19.16
1500	Male	Middle	Forest	8.82	0.44	50.78	20.14
1501	Male	Middle	Forest	39.38	0.05	30.14	14.79
1502	Male	Middle	Forest	8.62	0.12	27.25	33.78
1503	Male	Middle	Forest	25.88	55.13	16.01	0.33
1504	Male	Middle	Forest	74.85	3.97	8.55	6.12
1505	Male	Middle	Forest	10.4	1.62	12.67	21.82
1506	Male	Middle	Forest	7.61	0.03	38.89	38.94
1507	Male	Middle	Forest	19.07	0.04	29.87	13.14
1508	Male	Middle	Forest	8.28	1.67	83.11	3.7
1509	Male	Middle	Forest	13.38	20.17	24.16	5.33
1510	Male	Middle	Forest	7.16	1.99	13.45	29.79
1511	Male	Middle	Forest	38.15	6.12	26.7	13.2
1512	Male	Middle	Forest	12.66	1.56	35.42	4.45
1513	Male	Middle	Forest	23.16	9.61	10.62	9.66
1514	Male	Middle	Forest	0	0	0	0
1515	Male	Middle	Forest	61.52	0.19	22.2	5.68
1516	Male	Toddler	Forest	54.72	1.26	30.12	4.07
1517	Male	Toddler	Forest	49.57	0.07	13.9	27.17
1518	Male	Toddler	Forest	14.07	0.8	28.33	23.69
1519	Male	Toddler	Forest	65.08	0.08	22	7.34
1520	Male	Toddler	Forest	24.9	5.52	21.29	5.11
1521	Male	Toddler	Forest	78.09	2.77	8.38	1.08
1522	Male	Toddler	Forest	15.24	0.5	55.5	8.01
1523	Male	Old	Forest	16.3	0.38	52.65	13.23
1524	Male	Old	Forest	0	0	0	0
1525	Male	Old	Forest	11.35	4.46	28.62	31.57
1526	Male	Old	Forest	34.07	3.14	2.99	6.56
1527	Male	Old	Forest	0	0	0	0
1528	Male	Old	Forest	7.9	10.62	22.54	50.81
1529	Male	Old	Forest	0	0	0	0
1530	Male	Old	Forest	7.69	0.24	55.77	13.9
1531	Male	Old	Forest	17.28	36.14	10.67	9.23
1532	Male	Old	Forest	39.36	23.67	4.76	25.4
1533	Male	Old	Forest	6.96	0.22	71.07	8.9
1534	Male	Old	Forest	0.42	50.29	1.98	38.72
1535	Male	Old	Forest	48.17	15.74	31.12	2.31
1536	Male	Youth	Forest	40.78	4.28	27.49	14.13
1537	Male	Youth	Forest	0	0	0	0
1538	Male	Youth	Forest	6.93	0.14	13.03	13.27

1539	Male	Youth	Forest	84.62	0.56	8.09	1.82
1540	Male	Youth	Forest	40.5	21.55	7.98	9.62
1541	Male	Youth	Forest	1.6	5.09	19.87	35.63
1542	Male	Youth	Forest	0	0	0	0
1543	Male	Youth	Forest	60.74	0.96	5.95	7.17
1544	Male	Youth	Forest	38.28	0.46	36.36	14.39
1545	Male	Youth	Forest	40.17	0.38	17.38	29.18
1546	Male	Youth	Forest	54.23	2.71	6.86	12.75
1547	Male	Youth	Forest	17.87	73.65	2.1	3.55
1548	Male	Youth	Forest	9.65	0.85	26.4	12.37
1549	Male	Youth	Forest	30.68	0.89	12.65	41.54
1550	Male	Youth	Forest	0	0	0	0
1551	Male	Youth	Forest	49.94	7.26	15.2	9.14
1552	Female	Middle	Urban	12.99	0.04	66.86	16.53
1553	Female	Middle	Urban	24.61	1	57.37	10.17
1554	Female	Middle	Urban	34.87	7.51	19.45	21.3
1555	Female	Middle	Urban	0	0	0	0
1556	Female	Middle	Urban	30.78	0.34	42.46	11.61
1557	Female	Middle	Urban	14.9	0.62	24.25	19.3
1558	Female	Middle	Urban	1.74	3.58	2.11	24.37
1559	Female	Middle	Urban	5.55	0.18	72.54	8.03
1560	Female	Middle	Urban	3.77	0.28	83.67	3.56
1561	Female	Middle	Urban	10.79	0.21	47.72	5.39
1562	Female	Middle	Urban	17.39	2.13	56.41	5.55
1563	Female	Middle	Urban	22.37	7.3	18.79	20.47
1564	Female	Middle	Urban	2.51	0.96	67.17	7.96
1565	Female	Middle	Urban	19.97	0.33	26.3	34.81
1566	Female	Middle	Urban	0	0	0	0
1567	Female	Middle	Urban	0	0	0	0
1568	Female	Middle	Urban	79.96	3.16	7.15	2.82
1569	Female	Middle	Urban	27.72	3.61	36.92	16.05
1570	Female	Middle	Urban	65.96	8.2	13.4	2.36
1571	Female	Middle	Urban	9.96	0.92	11.79	13.79
1572	Female	Old	Urban	10.51	0.44	61.11	15.77
1573	Female	Old	Urban	0	0	0	0
1574	Female	Old	Urban	8.41	32.18	34.87	8.13
1575	Female	Old	Urban	0.94	0.74	3.66	16.93
1576	Female	Youth	Urban	13.28	0.05	7.89	13.31
1577	Female	Youth	Urban	13	0.04	33.85	12.85
1578	Female	Youth	Urban	0	0	0	0
1579	Female	Youth	Urban	2.25	0.29	13.22	21.02
1580	Female	Youth	Urban	51.28	0.11	15.18	4.66
1581	Female	Youth	Urban	52.08	0.51	20.25	12.58
1582	Female	Youth	Urban	0.12	99.56	0.04	0.14
1583	Female	Youth	Urban	14.9	0.03	27.21	20.29
1584	Female	Youth	Urban	10.24	0.4	32.52	13.57
1585	Female	Youth	Urban	37.93	0.27	14.97	11.41
1586	Female	Youth	Urban	11.12	9.43	22.2	8.98
1587	Female	Youth	Urban	0	0	0	0
1588	Female	Youth	Urban	2.65	32.36	13.42	29.81
1589	Female	Youth	Urban	17.25	0.54	12.07	7.15
1590	Female	Youth	Urban	4.52	0.2	45.22	2.64
1591	Male	Middle	Urban	44.67	4.37	20.3	19.38
1592	Male	Middle	Urban	6.44	0.48	73.31	3.92
1593	Male	Middle	Urban	0	0	0	0
1594	Male	Middle	Urban	10.25	0.61	20.21	32.81
1595	Male	Middle	Urban	51.51	0.04	12.51	2.18

1596	Male	Middle	Urban	7.45	62.65	17.96	5.1
1597	Male	Middle	Urban	12.64	0.58	55.5	4.35
1598	Male	Middle	Urban	0	0	0	0
1599	Male	Middle	Urban	36.84	0.13	36.84	11.58
1600	Male	Middle	Urban	36.39	0.34	24.21	14.28
1601	Male	Middle	Urban	20.54	0.86	31.39	16.49
1602	Male	Middle	Urban	21.28	39.44	25.81	3.03
1603	Male	Middle	Urban	0	0	0	0
1604	Male	Middle	Urban	1.08	6.33	3.12	37.27
1605	Male	Middle	Urban	12.31	0.22	15.55	35.52
1606	Male	Middle	Urban	0	0	0	0
1607	Male	Middle	Urban	22.34	5.46	11.85	11.4
1608	Male	Middle	Urban	8.61	0.2	9.78	69.2
1609	Male	Middle	Urban	7.84	2.74	76.08	7.59
1610	Male	Middle	Urban	3.65	0.02	42.55	25.44
1611	Male	Middle	Urban	0	0	0	0
1612	Male	Middle	Urban	0	0	0	0
1613	Male	Middle	Urban	12.43	0.04	47.27	6.17
1614	Male	Old	Urban	0	0	0	0
1615	Male	Old	Urban	0	0	0	0
1616	Male	Old	Urban	7.46	3.28	35.88	17.82
1617	Male	Old	Urban	0	0	0	0
1618	Male	Old	Urban	21.93	0.58	54.7	14.92
1619	Male	Old	Urban	29.57	0.6	52.29	8.14
1620	Male	Old	Urban	4.41	0.39	7.32	4.93
1621	Male	Old	Urban	0.67	28.21	5.21	25.98
1622	Male	Old	Urban	0	0	0	0
1623	Male	Old	Urban	0	0	0	0
1624	Male	Old	Urban	23.06	4.98	46.89	7.49
1625	Male	Old	Urban	13.39	0.2	31.6	38.67
1626	Male	Old	Urban	0	0	0	0
1627	Male	Youth	Urban	0.18	27.24	0.35	66.03
1628	Male	Youth	Urban	72.71	0.75	13.22	6.95
1629	Male	Youth	Urban	67.19	1.78	12.39	8.38
1630	Male	Youth	Urban	52.65	0.06	20.09	19.74
1631	Male	Youth	Urban	15.62	0.09	32.88	25.69
1632	Male	Youth	Urban	5.12	0.05	12.94	59.48
1633	Male	Youth	Urban	3.18	1.6	63.77	4.45
1634	Male	Youth	Urban	28.28	0.18	21.16	11.5
1635	Male	Youth	Urban	28.8	0.59	43.84	12.3
1636	Male	Youth	Urban	3.84	15.87	17.76	19.46
1637	Male	Youth	Urban	57.29	3.05	23.03	3.01
1638	Male	Youth	Urban	7.11	0.25	10.54	61.52
1639	Male	Youth	Urban	25.49	0.02	46.68	9.55
1640	Male	Youth	Urban	23.49	0.62	40.89	23.85
1641	Male	Youth	Urban	0	0	0	0
1642	Male	Youth	Urban	1.88	6.87	50.35	28.8
1643	Male	Youth	Urban	0.73	0.33	13.01	66.49
1644	Male	Youth	Urban	3.49	1.82	50.34	31.9
1645	Male	Youth	Urban	0	0	0	0
1646	Male	Youth	Urban	0	0	0	0
1647	Male	Youth	Urban	10.88	24.1	5.53	55.61
1648	Male	Youth	Urban	39.63	0.2	15.03	36.52
1649	Female	Middle	Urban	0.3	4.67	54.62	20.72
1650	Female	Middle	Urban	70.73	0.01	17.81	4.11
1651	Female	Middle	Urban	60.31	20.31	9.85	2.92
1652	Female	Middle	Urban	12.93	0.87	37.35	8.53

1653	Female	Middle	Urban	28.04	14.78	42.02	8.66
1654	Female	Middle	Urban	50.7	2.05	21.38	16.67
1655	Female	Middle	Urban	71.03	0.49	15.2	7.53
1656	Female	Middle	Urban	4.11	2.57	49.84	10.18
1657	Female	Middle	Urban	37.16	0.32	44.93	6.13
1658	Female	Middle	Urban	0.63	0.01	7.27	6.82
1659	Female	Middle	Urban	0	0	0	0
1660	Female	Middle	Urban	12.04	0.02	47.38	5.54
1661	Female	Middle	Urban	5.48	0.22	14.08	16.15
1662	Female	Middle	Urban	0	0	0	0
1663	Female	Middle	Urban	24.88	0.51	70.27	1.2
1664	Female	Middle	Urban	8.2	22.78	58.6	2.62
1665	Female	Middle	Urban	4.98	0.28	61.25	7.95
1666	Female	Middle	Urban	47.56	0.22	23.27	16.21
1667	Female	Middle	Urban	9.92	1.76	44	5.76
1668	Female	Middle	Urban	46.29	0.03	29.25	19.14
1669	Female	Middle	Urban	13.78	0.91	43.47	20.28
1670	Female	Middle	Urban	0	0	0	0
1671	Female	Middle	Urban	68.96	1.28	18.75	5.37
1672	Female	Middle	Urban	26.12	4.58	53.1	2.87
1673	Female	Toddler	Urban	0	0	0	0
1674	Female	Old	Urban	52.66	2.53	5.14	37.83
1675	Female	Old	Urban	0	0	0	0
1676	Female	Old	Urban	22.25	0.54	31.83	17.2
1677	Female	Old	Urban	0.34	0.1	10.84	30.35
1678	Female	Old	Urban	0	0	0	0
1679	Female	Old	Urban	0	0	0	0
1680	Female	Old	Urban	1.68	1.63	32.18	35.78
1681	Female	Old	Urban	0	0	0	0
1682	Female	Old	Urban	8.49	2.86	53.31	13.21
1683	Female	Old	Urban	6.09	0.21	61.66	14.3
1684	Female	Old	Urban	14.81	0.08	68.62	4.81
1685	Female	Youth	Urban	2.52	0.35	4.3	4.2
1686	Female	Youth	Urban	4.84	0.26	35.19	11
1687	Female	Youth	Urban	20.77	10.9	26.39	8.05
1688	Female	Youth	Urban	14.92	1.27	19.43	50.98
1689	Female	Youth	Urban	55.94	0.34	16.66	3.67
1690	Female	Youth	Urban	20.85	7.4	11.67	26.26
1691	Female	Youth	Urban	17.35	8.42	57.37	5.24
1692	Female	Youth	Urban	49.26	0.04	14.12	0.74
1693	Female	Youth	Urban	31.23	8.77	30.7	10.72
1694	Female	Youth	Urban	24.58	19.12	9.17	16.57
1695	Female	Youth	Urban	67.42	0.19	5.87	10.85
1696	Female	Youth	Urban	57.98	0.73	21.78	7.51
1697	Female	Youth	Urban	9.91	1.17	47.44	9.21
1698	Female	Youth	Urban	8.82	31.21	11.72	30.99
1699	Female	Youth	Urban	29.82	0.07	44.78	6.16
1700	Female	Youth	Urban	49.22	0.73	32.39	8.67
1701	Male	Middle	Urban	3.75	5.94	25.79	5.84
1702	Male	Middle	Urban	13.14	1.52	24.05	18.97
1703	Male	Middle	Urban	26.81	5.57	40.33	9.25
1704	Male	Middle	Urban	14.78	0.37	15.17	51.02
1705	Male	Middle	Urban	1.94	7.85	16.97	13.64
1706	Male	Middle	Urban	59.04	3.81	18.02	6.48
1707	Male	Middle	Urban	24.52	13.11	40	8.53
1708	Male	Middle	Urban	22.87	7.78	34.92	7.12
1709	Male	Middle	Urban	0	0	0	0

1710	Male	Middle	Urban	0	0	0	0
1711	Male	Middle	Urban	0	0	0	0
1712	Male	Middle	Urban	0	0	0	0
1713	Male	Middle	Urban	5.64	0.17	32.32	40.9
1714	Male	Middle	Urban	3.66	0.25	21.67	13.79
1715	Male	Middle	Urban	0	0	0	0
1716	Male	Middle	Urban	0	0	0	0
1717	Male	Old	Urban	29.08	2.12	11.53	24.25
1718	Male	Old	Urban	0	0	0	0
1719	Male	Old	Urban	0	0	0	0
1720	Male	Old	Urban	13.6	0.08	18.33	52.64
1721	Male	Old	Urban	39.6	0.71	27.82	10.53
1722	Male	Old	Urban	15.16	3.18	45.95	9.88
1723	Male	Old	Urban	0	0	0	0
1724	Male	Old	Urban	5.1	1.32	25.85	19.43
1725	Male	Old	Urban	13.06	77.56	4.56	1.07
1726	Male	Old	Urban	0.45	0.02	14.66	3.7
1727	Male	Old	Urban	15.75	5.17	43.44	4.3
1728	Male	Old	Urban	13.48	2.59	51.9	8.41
1729	Male	Youth	Urban	34.46	2.6	37.43	6.56
1730	Male	Youth	Urban	39.32	3.19	5.2	13.8
1731	Male	Youth	Urban	58.49	11.44	8.24	11.08
1732	Male	Youth	Urban	22.59	0.03	49.77	10.33
1733	Male	Youth	Urban	16.64	0.22	39.88	11.62
1734	Male	Youth	Urban	0	0	0	0
1735	Male	Youth	Urban	25.69	3.46	10.62	42.28
1736	Male	Youth	Urban	23.39	0.66	33.53	9.11
1737	Male	Youth	Urban	15.83	3.06	13.73	15.79
1738	Male	Youth	Urban	16.41	0.25	47.23	16.48
1739	Male	Youth	Urban	9.45	8.59	40.58	13.35
1740	Male	Youth	Urban	22.87	0.51	14.14	30.02
1741	Male	Youth	Urban	60.77	1.93	6.3	9.04
1742	Male	Youth	Urban	42.63	26.48	19.97	7.96
1743	Male	Youth	Urban	56.45	28.56	2.69	1.81
1744	Male	Youth	Urban	0	0	0	0
1745	Female	Middle	Urban	5.49	0.09	51.61	23.25
1746	Female	Middle	Urban	3	0.62	53.66	10.21
1747	Female	Middle	Urban	1.62	4.44	53.74	8.37
1748	Female	Middle	Urban	27.2	5.49	39.2	6.77
1749	Female	Middle	Urban	79.67	4.34	5.02	3.95
1750	Female	Middle	Urban	11.59	0.16	13.99	25.72
1751	Female	Middle	Urban	0	0	0	0
1752	Female	Middle	Urban	36.07	2.13	22.77	9.97
1753	Female	Middle	Urban	15.57	0.54	56.71	9.23
1754	Female	Middle	Urban	3.63	0.47	25.32	43.41
1755	Female	Middle	Urban	2.9	0.6	13.81	19.25
1756	Female	Middle	Urban	0.93	18.05	6.82	24.27
1757	Female	Middle	Urban	26.47	34.04	32.27	2.34
1758	Female	Middle	Urban	0.41	28.01	2.77	46.83
1759	Female	Middle	Urban	5.21	0.01	5.77	19.81
1760	Female	Middle	Urban	5.5	0.02	36	11.27
1761	Female	Middle	Urban	50.96	0.57	10.64	17.79
1762	Female	Middle	Urban	7.45	0.96	70.76	6.63
1763	Female	Middle	Urban	11.16	16.44	19.48	24.73
1764	Female	Old	Urban	16.03	38.69	13.87	13.62
1765	Female	Old	Urban	10.59	0.98	36.28	39.46
1766	Female	Old	Urban	5.17	0.13	59.27	13.06

1767	Female	Old	Urban	74.32	2.07	16.75	4.13
1768	Female	Old	Urban	9.14	0.03	66.99	11.34
1769	Female	Old	Urban	50.99	22.56	8.68	10.67
1770	Female	Old	Urban	23	11.29	49.72	7.64
1771	Female	Old	Urban	0	0	0	0
1772	Female	Old	Urban	11.23	11.76	56.33	11.95
1773	Female	Old	Urban	12.45	0.12	66.98	5.55
1774	Female	Old	Urban	40.6	1.69	51.96	0.93
1775	Female	Old	Urban	54.84	1.63	5.54	29.28
1776	Female	Old	Urban	62.54	0.23	30.71	2.03
1777	Female	Old	Urban	3.67	4.54	24.8	34.05
1778	Female	Old	Urban	0	0	0	0
1779	Female	Youth	Urban	29.2	5.2	19.69	11.14
1780	Female	Youth	Urban	46.38	1.11	32.64	8.54
1781	Female	Youth	Urban	9.28	0.2	20.96	16.14
1782	Female	Youth	Urban	52.3	2.44	23.49	12.2
1783	Female	Youth	Urban	8.32	0.61	65.95	3.21
1784	Female	Youth	Urban	67.4	0.22	28.36	2
1785	Female	Youth	Urban	16.5	2.47	35.42	19.42
1786	Female	Youth	Urban	41.33	1.87	32.38	3.45
1787	Female	Youth	Urban	3.7	0.25	48.86	18.23
1788	Female	Youth	Urban	29.14	0.06	9.69	3.25
1789	Female	Youth	Urban	27.81	0.39	18.34	19.03
1790	Female	Youth	Urban	15.26	0.08	34.17	11.74
1791	Female	Youth	Urban	19.18	0.08	59.06	3.11
1792	Female	Youth	Urban	9.64	0.56	33.21	50.23
1793	Male	Middle	Urban	0	0	0	0
1794	Male	Middle	Urban	37.06	0.45	39.03	7.75
1795	Male	Middle	Urban	38.42	10.15	22.34	17.84
1796	Male	Middle	Urban	3.62	0.25	24.78	8.71
1797	Male	Middle	Urban	23.25	7.34	13.64	16.27
1798	Male	Middle	Urban	12.59	2.06	13.67	8.28
1799	Male	Middle	Urban	9.46	4.68	20.02	15.37
1800	Male	Middle	Urban	11.79	14.52	45.14	9.82
1801	Male	Middle	Urban	26.14	10.71	22.63	10.44
1802	Male	Middle	Urban	20.98	0.8	23.38	12.04
1803	Male	Middle	Urban	29.04	0.15	40.4	16.98
1804	Male	Middle	Urban	8.72	0.23	27.21	16.29
1805	Male	Middle	Urban	9.17	0.07	53.02	9.13
1806	Male	Middle	Urban	58.19	0.03	20.89	8.49
1807	Male	Middle	Urban	65.77	5.84	16.11	5.97
1808	Male	Middle	Urban	0	0	0	0
1809	Male	Toddler	Urban	32.57	10.9	21.11	26.35
1810	Male	Old	Urban	40.75	0.83	15.28	19.49
1811	Male	Old	Urban	4.36	0.7	11.92	17.3
1812	Male	Old	Urban	0.98	0.19	4.82	27.34
1813	Male	Old	Urban	22.05	3.17	37.53	11.11
1814	Male	Old	Urban	0.19	94.15	0.6	3.41
1815	Male	Old	Urban	2.47	0.51	9.91	24.6
1816	Male	Old	Urban	25.29	3.72	38.8	4.32
1817	Male	Old	Urban	23.86	0.51	51.01	6.78
1818	Male	Old	Urban	0	0	0	0
1819	Male	Old	Urban	0	0	0	0
1820	Male	Old	Urban	25.67	20.99	47.46	3.69
1821	Male	Old	Urban	41.78	3	27.34	18.48
1822	Male	Old	Urban	13.16	0.67	21.24	49.27
1823	Male	Old	Urban	2.35	4.29	6.42	19.1

1824	Male	Old	Urban	20.4	0.55	49.58	20.29
1825	Male	Youth	Urban	12.1	12.97	16.17	15.39
1826	Male	Youth	Urban	36.92	0.75	35.38	12.72
1827	Male	Youth	Urban	3.46	0.05	7.36	21.86
1828	Male	Youth	Urban	57.15	0.35	15.44	6.06
1829	Male	Youth	Urban	1.59	0.18	78.61	6.18
1830	Male	Youth	Urban	18.18	0.22	14.54	10.89
1831	Male	Youth	Urban	27.12	0.66	29.44	27.77
1832	Male	Youth	Urban	43.64	4.9	19.27	8.36
1833	Male	Youth	Urban	4.7	1.95	35.12	21.13
1834	Male	Youth	Urban	36.67	1.97	39.17	7.8
1835	Male	Youth	Urban	0	0	0	0
1836	Male	Youth	Urban	0.4	30.89	8.46	11.2
1837	Male	Youth	Urban	6.65	6.5	10.63	11.6
1838	Male	Youth	Urban	55.72	2.63	13.28	13.58
1839	Male	Youth	Urban	26.58	0.3	22.44	9.37
1840	Male	Youth	Urban	13.21	0.98	67.78	1.72
1841	Male	Youth	Urban	22.61	2.81	6.61	7.53
1842	Female	Middle	Urban	1.27	88.61	1.83	7.26
1843	Female	Middle	Urban	18.39	4.73	53	3.41
1844	Female	Middle	Urban	7.94	87.92	3.27	0.33
1845	Female	Middle	Urban	2.38	29.5	25.7	7.46
1846	Female	Middle	Urban	0	0	0	0
1847	Female	Middle	Urban	0.1	99.17	0.11	0.3
1848	Female	Middle	Urban	0.85	42.33	0.49	1.59
1849	Female	Middle	Urban	0.57	48.23	3.92	23.64
1850	Female	Middle	Urban	0.13	67.12	1.65	25.62
1851	Female	Middle	Urban	1.55	9.71	13.37	21.16
1852	Female	Middle	Urban	1.22	96.58	0.43	0.62
1853	Female	Middle	Urban	14.03	25.37	21.63	12.73
1854	Female	Middle	Urban	71.78	8.74	4.58	1.06
1855	Female	Middle	Urban	6.67	1.65	6.36	10.69
1856	Female	Middle	Urban	8.42	2.21	20.39	13.3
1857	Female	Middle	Urban	0.48	83.78	2.02	3.45
1858	Female	Middle	Urban	42.1	1.86	28.55	9.69
1859	Female	Middle	Urban	7.93	68.83	11.19	2.38
1860	Female	Middle	Urban	43.48	28.06	4.42	5.43
1861	Female	Middle	Urban	10.33	0.15	31.73	22.9
1862	Female	Middle	Urban	0.22	73.54	2.66	9.63
1863	Female	Middle	Urban	29.06	26.91	37.11	1.3
1864	Female	Middle	Urban	1.14	86.82	3.75	3.97
1865	Female	Toddler	Urban	12.8	21.29	18.26	29.7
1866	Female	Toddler	Urban	0	0	0	0
1867	Female	Toddler	Urban	1.3	98.52	0.08	0
1868	Female	Toddler	Urban	60.45	30.87	6.94	0.13
1869	Female	Toddler	Urban	11.03	84.91	2.89	0.41
1870	Female	Toddler	Urban	76.75	3.32	14.12	2.47
1871	Female	Toddler	Urban	0.17	95.77	1.45	1.94
1872	Female	Old	Urban	1.82	0.83	12.73	6.62
1873	Female	Old	Urban	12.94	0.5	73.01	3.68
1874	Female	Old	Urban	41.27	5.58	22.18	8.61
1875	Female	Old	Urban	0.09	99.85	0.04	0.01
1876	Female	Old	Urban	3.3	28.13	6.83	24.5
1877	Female	Old	Urban	2.98	61.19	24.34	1.16
1878	Female	Old	Urban	1.82	59.8	16.1	5.22
1879	Female	Youth	Urban	9.24	7.12	25.6	7.55
1880	Female	Youth	Urban	11.35	26.15	7.86	14.38

1881	Female	Youth	Urban	2.99	86.97	0.78	5.27
1882	Female	Youth	Urban	2.57	37.23	10.46	13.95
1883	Female	Youth	Urban	0	0	0	0
1884	Female	Youth	Urban	41.17	31.59	5.6	4.54
1885	Female	Youth	Urban	52.84	37.92	5.79	2.36
1886	Female	Youth	Urban	2.97	77.6	10.09	3.46
1887	Female	Youth	Urban	46.06	14.01	17.65	14.33
1888	Female	Youth	Urban	1.11	59.32	1.57	6.08
1889	Female	Youth	Urban	1.14	54.93	0.57	3.12
1890	Female	Youth	Urban	0.64	90.9	4.13	0.83
1891	Female	Youth	Urban	0.03	99.91	0.04	0
1892	Female	Youth	Urban	2.16	21.97	11.2	44.38
1893	Female	Youth	Urban	1.25	65.26	9.1	10.97
1894	Female	Youth	Urban	0.25	78.49	2.91	9.57
1895	Female	Youth	Urban	0	0	0	0
1896	Female	Youth	Urban	0.5	97.86	0.53	0.35
1897	Female	Youth	Urban	11.32	69.38	4.99	1.27
1898	Female	Youth	Urban	19.39	76.09	3.69	0.25
1899	Female	Youth	Urban	14.92	3.97	11.62	13.94
1900	Female	Youth	Urban	48.94	17.1	13.56	5.08
1901	Female	Youth	Urban	4.92	25.21	15.78	10.94
1902	Female	Youth	Urban	14.92	0.31	49.74	4.88
1903	Female	Youth	Urban	0.17	97.05	0.18	0.67
1904	Female	Youth	Urban	0.59	81.86	2.84	9.57
1905	Female	Youth	Urban	4.83	82.56	6.1	2.39
1906	Female	Youth	Urban	0.03	99.65	0.05	0.04
1907	Female	Youth	Urban	2.12	89.39	2.21	0.91
1908	Female	Youth	Urban	34.4	20.81	9.48	5.29
1909	Female	Youth	Urban	1.25	1.81	3.06	19.41
1910	Female	Youth	Urban	2.61	61.44	6.75	4.74
1911	Female	Youth	Urban	0.04	99.48	0.07	0.09
1912	Female	Youth	Urban	0.03	96.49	0.39	1.75
1913	Female	Youth	Urban	0.08	91.64	0.87	1.08
1914	Female	Youth	Urban	5.86	93.12	0.39	0.11
1915	Female	Youth	Urban	0.13	98.55	0.18	0.76
1916	Female	Youth	Urban	0	99.42	0.01	0.39
1917	Female	Youth	Urban	1.19	42.65	4.17	16.48
1918	Female	Youth	Urban	25.19	14.44	45.05	6.34
1919	Female	Youth	Urban	11.76	28.98	32.15	8.69
1920	Female	Youth	Urban	7.46	19.98	42.68	5.13
1921	Female	Youth	Urban	0.22	95.73	1.72	1.57
1922	Female	Youth	Urban	0.38	1.44	23.94	36.42
1923	Female	Youth	Urban	0	0	0	0
1924	Female	Youth	Urban	5.69	72.14	11.95	0.77
1925	Female	Youth	Urban	3.45	93.86	1.56	0.34
1926	Female	Youth	Urban	3.09	73.81	5.09	3.31
1927	Female	Youth	Urban	8.35	80.37	1.17	4.44
1928	Female	Youth	Urban	1.6	0.99	7.91	0.88
1929	Female	Youth	Urban	22.59	2.21	12	24.39
1930	Female	Youth	Urban	11.27	51.46	8.29	3.22
1931	Female	Youth	Urban	29.21	2.48	40.45	9.81
1932	Female	Youth	Urban	40.67	14.03	20.87	8.64
1933	Female	Youth	Urban	7.07	11.74	61.08	3.62
1934	Female	Youth	Urban	1.62	0.34	59.43	10.66
1935	Female	Youth	Urban	0.53	38.76	37.51	3.95
1936	Female	Youth	Urban	0	0	0	0
1937	Female	Youth	Urban	83.14	4.96	3.87	1.73

1938	Female	Youth	Urban	0.88	74.9	10.06	4.29
1939	Female	Youth	Urban	12.85	1.61	53.98	8.27
1940	Female	Youth	Urban	11.86	1.48	44.4	5.62
1941	Female	Youth	Urban	2.2	0.97	14.47	12.76
1942	Female	Youth	Urban	0.65	56.17	4.04	21.69
1943	Female	Youth	Urban	0.18	98.49	0.05	0.94
1944	Female	Youth	Urban	10.56	12.1	6.63	15.34
1945	Female	Youth	Urban	0	0	0	0
1946	Female	Youth	Urban	1.92	68.86	1.61	4.24
1947	Female	Youth	Urban	0.73	50.36	2.16	19.8
1948	Female	Youth	Urban	10.34	54.75	10.15	10.49
1949	Female	Youth	Urban	16.77	70.32	9.84	1.14
1950	Male	Middle	Urban	8.95	70.23	18.98	0.26
1951	Male	Middle	Urban	17.58	17.94	24.78	30.31
1952	Male	Middle	Urban	0	0	0	0
1953	Male	Middle	Urban	0	99.99	0	0
1954	Male	Middle	Urban	1.77	78.21	15.34	1.22
1955	Male	Middle	Urban	0	0	0	0
1956	Male	Middle	Urban	0.36	88.65	0.8	4.45
1957	Male	Middle	Urban	11.21	57.95	13.16	7.63
1958	Male	Middle	Urban	12.07	18.63	26.92	8.04
1959	Male	Middle	Urban	26.58	4.31	23.03	22.83
1960	Male	Middle	Urban	19.94	2.56	26.42	15.75
1961	Male	Middle	Urban	0.98	96.91	0.27	1.11
1962	Male	Middle	Urban	4.83	12.36	21.14	14.37
1963	Male	Middle	Urban	0.18	54.78	2.46	28.79
1964	Male	Middle	Urban	0.52	16.29	68.91	0.7
1965	Male	Middle	Urban	2.94	79.48	13.83	1.42
1966	Male	Middle	Urban	3.46	40.03	3.79	18.88
1967	Male	Middle	Urban	9.85	86.09	2.63	0.22
1968	Male	Middle	Urban	4.64	1.47	51.87	10.21
1969	Male	Middle	Urban	74.25	5.74	14.85	2.2
1970	Male	Middle	Urban	1.92	32.07	19.95	5.6
1971	Male	Middle	Urban	8.85	4.97	28.99	32.61
1972	Male	Middle	Urban	25.41	6.37	36.99	20.66
1973	Male	Middle	Urban	29.79	2.46	43.58	10.19
1974	Male	Middle	Urban	0.13	95.79	1.73	0.65
1975	Male	Middle	Urban	0	0	0	0
1976	Male	Middle	Urban	0	99.86	0.01	0.07
1977	Male	Middle	Urban	0	0	0	0
1978	Male	Toddler	Urban	20.02	16.97	53.18	1.76
1979	Male	Toddler	Urban	0	0	0	0
1980	Male	Toddler	Urban	2.7	9.86	52.37	18.03
1981	Male	Toddler	Urban	10.29	28.67	38.75	2.89
1982	Male	Toddler	Urban	0.61	68.32	3.87	17.4
1983	Male	Old	Urban	0.32	97.76	1.31	0.3
1984	Male	Old	Urban	34.11	37.19	16.45	4.4
1985	Male	Old	Urban	26.71	32.89	18.94	10.87
1986	Male	Old	Urban	22.07	38.99	16.05	5.26
1987	Male	Old	Urban	16.04	25.18	33.02	9.38
1988	Male	Old	Urban	2.33	85.7	4.07	2.62
1989	Male	Old	Urban	0.18	99.05	0.39	0.02
1990	Male	Old	Urban	1.83	94.79	0.91	0.81
1991	Male	Old	Urban	0	0	0	0
1992	Male	Old	Urban	0	0	0	0
1993	Male	Old	Urban	0.29	91.46	3.46	1.13
1994	Male	Old	Urban	0	0	0	0

1995	Male	Old	Urban	0	0	0	0
1996	Male	Old	Urban	3.03	82.35	5.68	6.4
1997	Male	Old	Urban	0.09	97.98	0.15	1.13
1998	Male	Old	Urban	0.06	99.57	0.13	0.06
1999	Male	Old	Urban	2.24	62.3	8.35	6.22
2000	Male	Old	Urban	4.8	2.83	20.91	4.18
2001	Male	Old	Urban	14.01	1.43	58.26	19.63
2002	Male	Old	Urban	0	0	0	0
2003	Male	Old	Urban	13.35	27.32	25.94	15.09
2004	Male	Old	Urban	0.13	99.22	0.14	0.36
2005	Male	Old	Urban	35.53	1.51	53.72	6.67
2006	Male	Old	Urban	2.01	66.09	21.71	2.27
2007	Male	Youth	Urban	0.07	99.13	0.14	0.38
2008	Male	Youth	Urban	0.2	92.18	0.61	2.01
2009	Male	Youth	Urban	0	0	0	0
2010	Male	Youth	Urban	25.37	42.75	6.43	1.9
2011	Male	Youth	Urban	5.1	79.42	2.2	9.06
2012	Male	Youth	Urban	49.11	13.81	21.35	8.74
2013	Male	Youth	Urban	9.78	17.54	20.02	12.69
2014	Male	Youth	Urban	0.03	99.92	0.01	0.02
2015	Male	Youth	Urban	0.2	94.18	0.27	3.28
2016	Male	Youth	Urban	5.04	46.59	19.5	13.47
2017	Male	Youth	Urban	17.86	8.43	31.86	9.36
2018	Male	Youth	Urban	0.13	99.86	0	0
2019	Male	Youth	Urban	25.4	35.15	15.14	4.67
2020	Male	Youth	Urban	2.84	38.75	16.48	7.31
2021	Male	Youth	Urban	0	0	0	0
2022	Male	Youth	Urban	5.25	0.16	49.64	4.49
2023	Male	Youth	Urban	4.59	3.59	43.9	1.21
2024	Male	Youth	Urban	91.56	1.21	5	1.42
2025	Male	Youth	Urban	0	0	0	0
2026	Male	Youth	Urban	0	0	0	0
2027	Male	Youth	Urban	5.98	20.97	3.54	8.08
2028	Male	Youth	Urban	22.34	71.86	3.18	0.94
2029	Male	Youth	Urban	0.77	77	6.67	5.77
2030	Male	Youth	Urban	3.73	91.31	2.68	1.05
2031	Male	Youth	Urban	3.82	61.29	6.62	21.94
2032	Male	Youth	Urban	3.52	19.94	14.1	11.51
2033	Male	Youth	Urban	11.8	70.18	3.85	5.11
2034	Male	Youth	Urban	10.77	50.98	21.21	6.32
2035	Male	Youth	Urban	28.56	2.02	19.26	12.43
2036	Male	Youth	Urban	3.88	37.99	5.7	5.01
2037	Male	Youth	Urban	0.98	43.51	2.84	7.8
2038	Male	Youth	Urban	0.24	96.14	1.03	0.81
2039	Male	Youth	Urban	0	0	0	0
2040	Male	Youth	Urban	3.53	28.31	11.71	26.04
2041	Male	Youth	Urban	19.29	6.12	10.53	28.74
2042	Male	Youth	Urban	1.91	73.28	9.97	1.98
2043	Male	Youth	Urban	1.39	44.05	0.62	34.99
2044	Male	Youth	Urban	0.91	91.28	0.49	3.32
2045	Male	Youth	Urban	26.6	67.81	4.04	0.49
2046	Male	Youth	Urban	7.64	15.81	16.3	7.67
2047	Male	Youth	Urban	1.09	79.8	3.12	2.08
2048	Male	Youth	Urban	0.32	0.18	3.67	7.84
2049	Male	Youth	Urban	0	0	0	0
2050	Male	Youth	Urban	0.01	96.1	0.33	2.06
2051	Male	Youth	Urban	0.2	40.57	0.29	18.85

2052	Male	Youth	Urban	1.44	94.68	2.1	0.43
2053	Male	Youth	Urban	1.16	95.38	3.01	0.1
2054	Male	Youth	Urban	2.07	5.01	51.64	5.71
2055	Male	Youth	Urban	1.08	0.03	2.43	10.71
2056	Male	Youth	Urban	77.33	3.72	6.45	5.64
2057	Male	Youth	Urban	45.66	13.66	11.02	11.85
2058	Male	Youth	Urban	0.49	70.32	4.51	17.87
2059	Male	Youth	Urban	3.08	0.86	8.67	18.66
2060	Male	Youth	Urban	37.18	24.6	14.15	11.58
2061	Male	Youth	Urban	0.44	95.27	1.05	1.6
2062	Male	Youth	Urban	0.07	99.75	0.09	0.02
2063	Male	Youth	Urban	27.14	30.32	1.79	20.23
2064	Male	Youth	Urban	0.08	98.81	0.07	0.84
2065	Male	Youth	Urban	0	0	0	0
2066	Male	Youth	Urban	4.47	41.71	2.63	2.4
2067	Male	Youth	Urban	11.28	34.81	18.88	10.88
2068	Male	Youth	Urban	0	0	0	0
2069	Male	Youth	Urban	1.88	46.19	2.21	5.52
2070	Male	Youth	Urban	0.48	55.3	2.85	18.31
2071	Male	Youth	Urban	0.14	98.52	0.33	0.67
2072	Male	Youth	Urban	0	0	0	0
2073	Male	Youth	Urban	4.3	76.04	9.31	4.42
2074	Male	Youth	Urban	7.31	14.73	33.22	21.81
2075	Male	Youth	Urban	23.89	41.59	11	3.66
2076	Female	Middle	Urban	0	0	0	0
2077	Female	Middle	Urban	0	0	0	0
2078	Female	Middle	Urban	7.87	0.01	20.64	60.59
2079	Female	Middle	Urban	67.71	9.02	9.57	2.33
2080	Female	Middle	Urban	28.47	0.03	48.44	15.06
2081	Female	Middle	Urban	0	0	0	0
2082	Female	Middle	Urban	55.71	11.34	21.64	8.17
2083	Female	Middle	Urban	66.39	10.62	16.85	3.27
2084	Female	Middle	Urban	38.88	0.12	40.15	10.51
2085	Female	Middle	Urban	22.78	0.04	66.44	2.42
2086	Female	Middle	Urban	3.85	0.03	83.57	2.07
2087	Female	Middle	Urban	23.9	0.36	26.9	27.69
2088	Female	Middle	Urban	12.98	0.32	45.1	9.44
2089	Female	Middle	Urban	10.07	0.06	26.01	21.7
2090	Female	Middle	Urban	14.73	1.56	48.52	6.61
2091	Female	Middle	Urban	41.71	0.12	43.5	6.68
2092	Female	Middle	Urban	49.81	6.75	39.15	2.39
2093	Female	Middle	Urban	56.64	5.01	12.4	12.96
2094	Female	Middle	Urban	19.3	63.83	8.65	2.83
2095	Female	Middle	Urban	11.09	0.15	36.04	38.14
2096	Female	Middle	Urban	7.48	2.07	13.68	21.48
2097	Female	Middle	Urban	9.09	3.57	17.05	19.2
2098	Female	Middle	Urban	20.85	0.7	28.31	26.87
2099	Female	Middle	Urban	17.81	6.1	11.48	32.15
2100	Female	Middle	Urban	21.94	1.78	48.62	3.17
2101	Female	Middle	Urban	41.45	0.32	39.21	8.91
2102	Female	Middle	Urban	13.13	11.91	5.93	22.79
2103	Female	Middle	Urban	2.75	1.31	23.57	5.86
2104	Female	Middle	Urban	7.2	0.57	8.42	71.7
2105	Female	Middle	Urban	15.22	0.62	45.02	2.95
2106	Female	Middle	Urban	22.76	0.47	52.96	15.62
2107	Female	Middle	Urban	33.75	1.81	2.43	1.72
2108	Female	Middle	Urban	12.95	0.28	45.31	19.08

2109	Female	Middle	Urban	35.26	34.22	4.41	2.8
2110	Female	Middle	Urban	8.64	0.36	22.2	9.28
2111	Female	Middle	Urban	7.57	8.94	21.81	17.56
2112	Female	Middle	Urban	28.84	0.04	60.78	7.97
2113	Female	Middle	Urban	28.59	1.12	21.02	32.56
2114	Female	Middle	Urban	56.08	7.16	18.38	9.02
2115	Female	Middle	Urban	26.48	0.18	42.47	10.03
2116	Female	Middle	Urban	0	0	0	0
2117	Female	Middle	Urban	49.04	0.13	27.4	12.51
2118	Female	Middle	Urban	44.51	1.48	36.41	9.05
2119	Female	Middle	Urban	40.46	8.54	19.4	5.44
2120	Female	Middle	Urban	18.9	1.94	66.05	3.46
2121	Female	Middle	Urban	88.07	0.15	5.02	5.48
2122	Female	Middle	Urban	30.19	0.14	12.58	14.39
2123	Female	Middle	Urban	0	0	0	0
2124	Female	Middle	Urban	10.35	0.45	43.38	25.42
2125	Female	Middle	Urban	65.6	3.96	15.16	9.16
2126	Female	Middle	Urban	18.41	0.25	50.6	11.13
2127	Female	Middle	Urban	3.62	0.09	52.87	16.13
2128	Female	Middle	Urban	0	0	0	0
2129	Female	Middle	Urban	3.99	0.45	8.32	5.29
2130	Female	Middle	Urban	12.52	0.26	5.65	10.42
2131	Female	Middle	Urban	0	0	0	0
2132	Female	Middle	Urban	34.03	0.94	21.71	17.61
2133	Female	Middle	Urban	14.73	2.07	56.6	2.52
2134	Female	Middle	Urban	3.16	0.09	43.8	20.37
2135	Female	Middle	Urban	29.61	0.07	60.13	1.15
2136	Female	Middle	Urban	52.82	0.53	34.47	3.64
2137	Female	Middle	Urban	68.18	1.76	15.68	6.01
2138	Female	Middle	Urban	51.3	0.69	20.09	9.79
2139	Female	Middle	Urban	53.89	0.07	25.19	7.76
2140	Female	Middle	Urban	9.4	0.05	27.97	18.96
2141	Female	Middle	Urban	30.25	1.58	35.46	18.06
2142	Female	Middle	Urban	31.4	0.34	38.99	3.97
2143	Female	Middle	Urban	25.21	0.5	31	21.76
2144	Female	Middle	Urban	44.18	24.9	8.92	4.57
2145	Female	Middle	Urban	12.01	3.88	19	28.86
2146	Female	Middle	Urban	4.82	0.06	31.74	35.13
2147	Female	Middle	Urban	19	0.16	16.36	17.56
2148	Female	Middle	Urban	16.39	0.72	17.93	34.46
2149	Female	Middle	Urban	56.27	1.7	31.02	6.14
2150	Female	Middle	Urban	41.35	2.39	42.78	4.59
2151	Female	Middle	Urban	45.43	1.36	41.22	5.76
2152	Female	Middle	Urban	0.96	0.58	13.55	1.95
2153	Female	Middle	Urban	1.41	0	71.81	1.19
2154	Female	Middle	Urban	12.87	3.06	25.97	14.66
2155	Female	Middle	Urban	48.67	8.34	9	13.6
2156	Female	Middle	Urban	0	0	0	0
2157	Female	Middle	Urban	56.14	1.46	20.27	4.46
2158	Female	Middle	Urban	28.76	17.49	28.94	7.69
2159	Female	Middle	Urban	6.49	0.11	61.14	2.03
2160	Female	Middle	Urban	38.71	0.5	31.81	9.17
2161	Female	Middle	Urban	5	0.25	16.62	5.59
2162	Female	Middle	Urban	4.29	0.63	20.03	30.32
2163	Female	Middle	Urban	42.69	9.57	24.23	5.38
2164	Female	Middle	Urban	13.77	2.06	9.57	56.06
2165	Female	Middle	Urban	76.55	3.2	5.15	5.46

2166	Female	Middle	Urban	0	0	0	0
2167	Female	Middle	Urban	11.68	0.6	19.69	22.87
2168	Female	Middle	Urban	34.79	0.08	31.64	4.04
2169	Female	Middle	Urban	12.89	25.18	47.1	5.11
2170	Female	Middle	Urban	22.89	0.15	65.48	3.67
2171	Female	Middle	Urban	43.24	1.31	35.62	9.99
2172	Female	Middle	Urban	0	0	0	0
2173	Female	Toddler	Urban	80.08	8.88	8.26	0.76
2174	Female	Toddler	Urban	82.2	0.56	7.67	8.16
2175	Female	Toddler	Urban	38.85	2.78	18.53	3.95
2176	Female	Toddler	Urban	88.71	0.12	8.45	1.68
2177	Female	Toddler	Urban	92.04	0.29	2.95	3.99
2178	Female	Toddler	Urban	78.68	1.26	10.81	6.19
2179	Female	Toddler	Urban	39.71	0.66	21.92	10.55
2180	Female	Old	Urban	44.66	2.31	36.03	8.82
2181	Female	Old	Urban	54.77	12.29	9.01	4.99
2182	Female	Old	Urban	38.56	0.94	19.34	37.18
2183	Female	Old	Urban	22.91	0.29	54.17	4.87
2184	Female	Old	Urban	0.07	85.51	0.67	12.18
2185	Female	Old	Urban	14.65	0.61	35.35	28.77
2186	Female	Old	Urban	35.63	4.19	20.33	30.52
2187	Female	Old	Urban	0	0	0	0
2188	Female	Old	Urban	0	0	0	0
2189	Female	Old	Urban	11.37	22.06	51.07	1.41
2190	Female	Old	Urban	14.46	7.55	26.41	28.91
2191	Female	Old	Urban	0	0	0	0
2192	Female	Old	Urban	7.64	0.06	50.34	5.53
2193	Female	Old	Urban	9.5	2.14	20.52	36.86
2194	Female	Old	Urban	8.11	7.07	40.4	36.93
2195	Female	Old	Urban	20.14	0.81	41.8	3.98
2196	Female	Old	Urban	6.08	59.83	8.67	6.58
2197	Female	Old	Urban	16.64	0.76	29.05	11.08
2198	Female	Old	Urban	37.86	1.89	19.06	5.26
2199	Female	Old	Urban	54.02	9.23	27.78	2.8
2200	Female	Old	Urban	17.69	0.15	20.42	14.72
2201	Female	Old	Urban	47.41	3.88	37.62	6.89
2202	Female	Old	Urban	11.71	0.29	46.68	31.42
2203	Female	Old	Urban	16.7	0.13	42.97	4.81
2204	Female	Old	Urban	12.91	1.33	30.49	8.66
2205	Female	Old	Urban	16.38	0.22	33.27	20.39
2206	Female	Youth	Urban	12.46	29.2	2.28	5.2
2207	Female	Youth	Urban	13.58	0.23	33.7	16.29
2208	Female	Youth	Urban	35.85	0.07	47.62	9.07
2209	Female	Youth	Urban	93.03	0.19	4.46	0.7
2210	Female	Youth	Urban	25.09	0.32	33.74	24.05
2211	Female	Youth	Urban	27.17	2.93	38.99	17.2
2212	Female	Youth	Urban	0	0	0	0
2213	Female	Youth	Urban	25.25	7.79	20.12	29.44
2214	Female	Youth	Urban	59.26	1.98	12.3	9.16
2215	Female	Youth	Urban	4.21	0.15	53.2	1.64
2216	Female	Youth	Urban	13.27	23.96	34.15	3.03
2217	Female	Youth	Urban	8.17	0.08	66.11	1.91
2218	Female	Youth	Urban	43.27	0.06	46.11	1.37
2219	Female	Youth	Urban	8.51	0.36	78.53	1.6
2220	Female	Youth	Urban	0	0	0	0
2221	Female	Youth	Urban	5.21	0.05	74.4	5.2
2222	Female	Youth	Urban	0.76	0.01	51.03	6.46

2223	Female	Youth	Urban	50.03	2.64	10.67	7.49
2224	Female	Youth	Urban	0	0	0	0
2225	Female	Youth	Urban	2.84	27.14	14.28	37.24
2226	Female	Youth	Urban	27.32	6.45	31.78	17.03
2227	Female	Youth	Urban	27.6	3.65	19.71	22.1
2228	Female	Youth	Urban	51.39	0.1	27.16	8.72
2229	Female	Youth	Urban	24	3.13	47.85	6.39
2230	Female	Youth	Urban	13.43	0.05	18.92	24.4
2231	Female	Youth	Urban	45.12	1.87	37.96	5.68
2232	Female	Youth	Urban	10.82	0.26	16.51	16.23
2233	Female	Youth	Urban	1.33	0.02	78.18	2.07
2234	Female	Youth	Urban	62.19	0.26	23.2	4.24
2235	Female	Youth	Urban	4.81	3.41	33.57	35.91
2236	Female	Youth	Urban	13.42	0.06	58.89	7.66
2237	Female	Youth	Urban	37.62	0.07	48.71	4.77
2238	Female	Youth	Urban	60.76	0.81	21.84	5.26
2239	Female	Youth	Urban	11.43	2.05	9.32	36.31
2240	Female	Youth	Urban	7.91	0.83	41.47	24.97
2241	Female	Youth	Urban	18.88	0.41	25.47	20.05
2242	Female	Youth	Urban	15.25	0.21	9.13	53.98
2243	Female	Youth	Urban	5.46	0.95	24.59	9.78
2244	Female	Youth	Urban	44.85	0.6	30.57	3.17
2245	Female	Youth	Urban	0	0	0	0
2246	Female	Youth	Urban	2.9	0.28	4.93	15.83
2247	Female	Youth	Urban	29.72	13.15	16.35	4.93
2248	Female	Youth	Urban	7.92	0.14	44.68	5.83
2249	Female	Youth	Urban	21.43	6.31	32.63	4.02
2250	Female	Youth	Urban	3.34	0.24	4.77	13.04
2251	Female	Youth	Urban	84.96	0.49	8.15	2.29
2252	Female	Youth	Urban	49.38	0.46	16.83	14.16
2253	Female	Youth	Urban	0	0	0	0
2254	Female	Youth	Urban	50.94	0.91	32.33	5.15
2255	Female	Youth	Urban	24.27	0.07	19.93	13.94
2256	Female	Youth	Urban	5.45	0.6	18.9	3.22
2257	Female	Youth	Urban	29.2	5.2	19.69	11.14
2258	Female	Youth	Urban	17.84	0.76	49.54	2.7
2259	Female	Youth	Urban	32.91	0.37	20.27	18.11
2260	Female	Youth	Urban	5.36	0.01	24.75	42.02
2261	Female	Youth	Urban	81.16	2.57	11.07	1.43
2262	Female	Youth	Urban	26.04	0.18	20.34	33.74
2263	Female	Youth	Urban	56.8	1.65	28.14	5.44
2264	Female	Youth	Urban	1.37	0.02	46.78	5.82
2265	Female	Youth	Urban	64.23	1.04	17.55	9.04
2266	Female	Youth	Urban	39.76	0.01	33.84	23.61
2267	Female	Youth	Urban	25.55	0.7	44.01	10.35
2268	Female	Youth	Urban	53.89	1.41	25.7	5.43
2269	Female	Youth	Urban	44.22	9.94	11.71	7.14
2270	Female	Youth	Urban	52.87	1.15	21.53	10.67
2271	Female	Youth	Urban	8.95	0.46	52.66	2.32
2272	Female	Youth	Urban	85.99	0.31	8.32	1.47
2273	Female	Youth	Urban	49.04	2.38	16.23	9.51
2274	Female	Youth	Urban	5.58	0.62	57.35	5.51
2275	Female	Youth	Urban	41.22	1.3	43.39	1.91
2276	Female	Youth	Urban	1.63	14.07	7.76	17.73
2277	Female	Youth	Urban	0	0	0	0
2278	Female	Youth	Urban	32.63	0.8	19.21	4.68
2279	Female	Youth	Urban	17.61	0.03	58.04	1.34

2280	Female	Youth	Urban	23.65	10.5	26.8	7.66
2281	Female	Youth	Urban	0.96	34.2	2.97	38.4
2282	Female	Youth	Urban	22.31	0.27	27.7	17.03
2283	Female	Youth	Urban	25.15	0.34	31.66	9.86
2284	Female	Youth	Urban	7.1	0.68	33.7	22.29
2285	Female	Youth	Urban	17.37	0.24	67.71	4.84
2286	Female	Youth	Urban	11.38	0.2	68.24	6.87
2287	Female	Youth	Urban	0	0	0	0
2288	Female	Youth	Urban	21.93	9.5	12.48	22.81
2289	Female	Youth	Urban	9.89	1.77	15.04	20.26
2290	Female	Youth	Urban	48.23	9.43	30.14	7.25
2291	Female	Youth	Urban	51.81	2.25	14.5	23.06
2292	Female	Youth	Urban	23.87	0.31	28.55	25.05
2293	Female	Youth	Urban	9.5	69.84	6.18	9.42
2294	Female	Youth	Urban	20.17	2.53	25.45	7.29
2295	Female	Youth	Urban	59.73	0.44	20.92	7
2296	Female	Youth	Urban	15.38	2.37	24.91	8.72
2297	Female	Youth	Urban	21.34	0.56	19.23	17.6
2298	Female	Youth	Urban	0.21	1.11	12.42	28.32
2299	Female	Youth	Urban	0	0	0	0
2300	Female	Youth	Urban	5.7	0.19	35.33	10.85
2301	Female	Youth	Urban	49.05	0.09	22.87	1.88
2302	Female	Youth	Urban	22.73	0.1	55.15	4.1
2303	Female	Youth	Urban	3.42	14.74	9.47	51.39
2304	Female	Youth	Urban	43.22	0.55	33.57	10.68
2305	Female	Youth	Urban	40.87	0.58	34.01	6.4
2306	Female	Youth	Urban	37.01	5.01	5.83	9.74
2307	Female	Youth	Urban	33.6	0.98	34.67	15.4
2308	Female	Youth	Urban	58.01	2.74	28.23	1.35
2309	Female	Youth	Urban	17.23	0.56	28.47	27.56
2310	Female	Youth	Urban	28.95	0.66	37.36	14.05
2311	Female	Youth	Urban	0.57	1.03	15.88	45.12
2312	Female	Youth	Urban	73.66	3.14	5.07	7.75
2313	Female	Youth	Urban	30.86	0.05	53.38	3.8
2314	Female	Youth	Urban	72.75	0.27	8.74	12.56
2315	Male	Middle	Urban	49.64	0.24	18.35	15.17
2316	Male	Middle	Urban	38.46	4.13	24.84	5.96
2317	Male	Middle	Urban	30.32	0.22	45.7	7.77
2318	Male	Middle	Urban	36.25	9.38	14.46	18.75
2319	Male	Middle	Urban	14.99	1.16	10.68	2.74
2320	Male	Middle	Urban	79.16	5.98	3.86	3.78
2321	Male	Middle	Urban	77.51	0.28	15.22	3.59
2322	Male	Middle	Urban	7.2	0.06	35.34	17.74
2323	Male	Middle	Urban	54.48	1.36	27.75	6.93
2324	Male	Middle	Urban	11.59	2.32	45.85	6.9
2325	Male	Middle	Urban	21.29	0.24	14.37	24.52
2326	Male	Middle	Urban	14.78	0.37	15.17	51.02
2327	Male	Middle	Urban	14.15	0.14	50.03	22.57
2328	Male	Middle	Urban	10.79	1.45	21.04	24.05
2329	Male	Middle	Urban	48.72	0.57	26.74	18.16
2330	Male	Middle	Urban	0	0	0	0
2331	Male	Middle	Urban	0	0	0	0
2332	Male	Middle	Urban	4.75	0.07	59.65	16.47
2333	Male	Middle	Urban	6.71	62.95	12.71	3.78
2334	Male	Middle	Urban	21.77	2.66	52.9	2.25
2335	Male	Middle	Urban	31.54	0.68	24.96	15.71
2336	Male	Middle	Urban	36.29	0.59	30.8	10.7

2337	Male	Middle	Urban	2.18	3.22	53.79	7.65
2338	Male	Middle	Urban	19.6	3.06	47.5	6.08
2339	Male	Middle	Urban	38.94	0.6	42.14	10.07
2340	Male	Middle	Urban	26.29	17.6	10.28	36.28
2341	Male	Middle	Urban	11.59	0.24	26.65	36.74
2342	Male	Middle	Urban	27.24	0.19	24.21	30.99
2343	Male	Middle	Urban	0	0	0	0
2344	Male	Middle	Urban	0	0	0	0
2345	Male	Middle	Urban	36.03	12.9	28.37	12.01
2346	Male	Middle	Urban	0	0	0	0
2347	Male	Middle	Urban	0	0	0	0
2348	Male	Middle	Urban	57.9	0.16	18.96	12.81
2349	Male	Middle	Urban	70.66	2.52	4.34	16.38
2350	Male	Middle	Urban	29.56	1.07	23.95	8.33
2351	Male	Middle	Urban	48.56	3.06	15.34	23.14
2352	Male	Middle	Urban	39.81	0.69	26.28	13.36
2353	Male	Middle	Urban	0	0	0	0
2354	Male	Middle	Urban	22.59	1.56	14.05	27.61
2355	Male	Middle	Urban	17.72	12.25	44.24	13.53
2356	Male	Middle	Urban	16.25	0.04	70.34	6.23
2357	Male	Middle	Urban	13.84	0.03	66.5	9.39
2358	Male	Middle	Urban	0	0	0	0
2359	Male	Middle	Urban	8.88	0.57	4.92	8.22
2360	Male	Middle	Urban	35.36	0.13	49.77	2.74
2361	Male	Middle	Urban	37.3	27.25	21.03	7.45
2362	Male	Middle	Urban	7.81	0.91	30.76	10.79
2363	Male	Middle	Urban	34.84	1.76	50.97	5.2
2364	Male	Middle	Urban	20.29	1.1	17.1	38.81
2365	Male	Middle	Urban	35.62	0.73	44.74	10.05
2366	Male	Middle	Urban	4.27	15.48	14.77	18.8
2367	Male	Middle	Urban	29.35	1.1	24.41	7.93
2368	Male	Middle	Urban	0	0	0	0
2369	Male	Middle	Urban	38.62	0.37	28.01	17.64
2370	Male	Middle	Urban	5.62	0.48	52.85	22.15
2371	Male	Middle	Urban	74.22	0.28	9.88	9.89
2372	Male	Middle	Urban	29.75	1.11	18.77	18.37
2373	Male	Middle	Urban	10.48	0.36	18.82	5.33
2374	Male	Middle	Urban	27.49	0.01	41.25	21.09
2375	Male	Middle	Urban	0	0	0	0
2376	Male	Middle	Urban	23.62	0.13	46.56	19.93
2377	Male	Middle	Urban	20.98	2.9	20.78	34.23
2378	Male	Middle	Urban	8.92	1.33	20.97	21.56
2379	Male	Middle	Urban	32.97	4.99	51.63	3.3
2380	Male	Middle	Urban	6.41	0.39	45.29	25.13
2381	Male	Middle	Urban	27.8	3.25	44.48	7.77
2382	Male	Middle	Urban	0	0	0	0
2383	Male	Middle	Urban	24.47	0.08	45.28	13.54
2384	Male	Middle	Urban	15.69	2.1	3.19	7.43
2385	Male	Middle	Urban	19.06	0.18	17.89	16.77
2386	Male	Middle	Urban	46.74	0.44	20.62	15.85
2387	Male	Middle	Urban	58.97	13.1	7.46	15.61
2388	Male	Middle	Urban	15.15	3.63	68.28	4.96
2389	Male	Toddler	Urban	0	0	0	0
2390	Male	Toddler	Urban	0	0	0	0
2391	Male	Toddler	Urban	0	0	0	0
2392	Male	Toddler	Urban	20.24	2.64	52.59	11.41
2393	Male	Toddler	Urban	36.29	31.65	3.3	19.51

2394	Male	Toddler	Urban	31.04	3.18	36.91	9.17
2395	Male	Toddler	Urban	68.23	1.98	18.67	1.54
2396	Male	Toddler	Urban	0	0	0	0
2397	Male	Toddler	Urban	22.9	0.9	32.31	15.32
2398	Male	Toddler	Urban	68.47	2.64	23.18	3.51
2399	Male	Toddler	Urban	14.53	1.75	18.76	22.97
2400	Male	Toddler	Urban	20.85	2.62	29.45	12.66
2401	Male	Toddler	Urban	20.36	6.63	21.79	23.9
2402	Male	Toddler	Urban	0	0	0	0
2403	Male	Toddler	Urban	65.82	0.57	14.19	6.13
2404	Male	Toddler	Urban	48.73	11.15	16.38	14.01
2405	Male	Toddler	Urban	0	0	0	0
2406	Male	Toddler	Urban	24.5	0.17	60.52	1.18
2407	Male	Old	Urban	18.49	11.14	14.83	24.96
2408	Male	Old	Urban	30.11	8.33	25.14	16.59
2409	Male	Old	Urban	12.47	3	16.65	41.3
2410	Male	Old	Urban	24.04	16.96	20.74	9.88
2411	Male	Old	Urban	34.79	7.92	21.73	18.31
2412	Male	Old	Urban	19.13	7.7	37.91	19.21
2413	Male	Old	Urban	13.13	38.24	6.85	38.46
2414	Male	Old	Urban	34.09	1.1	23.01	17.04
2415	Male	Old	Urban	23.89	0.47	23.66	28.75
2416	Male	Old	Urban	0	0	0	0
2417	Male	Old	Urban	30.93	2.24	41.86	3.7
2418	Male	Old	Urban	13.81	0.53	45.67	7.54
2419	Male	Old	Urban	0	0	0	0
2420	Male	Old	Urban	0	0	0	0
2421	Male	Old	Urban	5.64	0.03	60.73	15.59
2422	Male	Old	Urban	6.18	1.65	72.88	7.75
2423	Male	Old	Urban	0	0	0	0
2424	Male	Old	Urban	13.82	0.93	62.72	11.43
2425	Male	Old	Urban	0	0	0	0
2426	Male	Old	Urban	7.59	19.14	60.29	3.22
2427	Male	Old	Urban	0	0	0	0
2428	Male	Old	Urban	0	0	0	0
2429	Male	Old	Urban	26.38	0.39	49.06	3.67
2430	Male	Old	Urban	0	0	0	0
2431	Male	Old	Urban	7.96	0.21	48.09	8.98
2432	Male	Old	Urban	0	0	0	0
2433	Male	Old	Urban	0	0	0	0
2434	Male	Old	Urban	0	0	0	0
2435	Male	Old	Urban	18.34	4.12	53.83	11.11
2436	Male	Old	Urban	48.25	0.39	21.49	13.88
2437	Male	Old	Urban	0	0	0	0
2438	Male	Old	Urban	14.93	1.39	34.27	41.84
2439	Male	Old	Urban	17.13	0.9	28.51	24.67
2440	Male	Old	Urban	2.27	17.59	4.39	35.96
2441	Male	Old	Urban	0	0	0	0
2442	Male	Old	Urban	0	0	0	0
2443	Male	Old	Urban	0	0	0	0
2444	Male	Old	Urban	0	0	0	0
2445	Male	Old	Urban	14.03	13.57	10.62	34.5
2446	Male	Old	Urban	5.69	0.82	43.61	22.41
2447	Male	Old	Urban	12	0.43	12.87	9.61
2448	Male	Old	Urban	0	0	0	0
2449	Male	Old	Urban	9.63	0.17	75.51	4.02
2450	Male	Old	Urban	45.48	0.29	45.93	3.93

2451	Male	Old	Urban	9	5.47	54.48	14.94
2452	Male	Old	Urban	15.97	0.57	45.9	12.23
2453	Male	Old	Urban	0	0	0	0
2454	Male	Old	Urban	0	0	0	0
2455	Male	Old	Urban	0	0	0	0
2456	Male	Old	Urban	14.66	0.09	48.17	7.89
2457	Male	Old	Urban	0	0	0	0
2458	Male	Old	Urban	0	0	0	0
2459	Male	Old	Urban	22.68	0.02	19.05	39.91
2460	Male	Old	Urban	44.32	3.47	19.34	17.07
2461	Male	Old	Urban	3.73	0.48	31.35	27.6
2462	Male	Old	Urban	16.74	1.57	67.82	7.75
2463	Male	Old	Urban	9.9	0.55	33.61	13.43
2464	Male	Old	Urban	47.04	4.24	23.73	11.04
2465	Male	Old	Urban	5.59	0.33	32.46	23.81
2466	Male	Youth	Urban	8.32	7.8	8.12	23.74
2467	Male	Youth	Urban	5.13	0.06	66.93	6.81
2468	Male	Youth	Urban	8.03	0.28	27.81	11.93
2469	Male	Youth	Urban	31.82	1.65	53.93	4.51
2470	Male	Youth	Urban	14.45	4.33	25.76	19.41
2471	Male	Youth	Urban	24.04	1.1	15.07	15.38
2472	Male	Youth	Urban	35.33	1.01	28.24	6.98
2473	Male	Youth	Urban	16.57	1.43	22.03	13.48
2474	Male	Youth	Urban	0	0	0	0
2475	Male	Youth	Urban	29.05	0.06	36.2	6.92
2476	Male	Youth	Urban	2.06	0.15	9.8	27.65
2477	Male	Youth	Urban	0	0	0	0
2478	Male	Youth	Urban	0	0	0	0
2479	Male	Youth	Urban	50.51	0.38	29.7	6.3
2480	Male	Youth	Urban	12.72	19.34	4.71	24.91
2481	Male	Youth	Urban	0	0	0	0
2482	Male	Youth	Urban	30.98	0.46	38.76	10.29
2483	Male	Youth	Urban	36.12	1.4	23.97	19.72
2484	Male	Youth	Urban	20.93	0.51	17.48	22.57
2485	Male	Youth	Urban	83.75	0.51	8.11	5.47
2486	Male	Youth	Urban	0	0	0	0
2487	Male	Youth	Urban	0	0	0	0
2488	Male	Youth	Urban	5.44	23.77	4.1	54.36
2489	Male	Youth	Urban	0	0	0	0
2490	Male	Youth	Urban	89.33	0.07	2.61	1.58
2491	Male	Youth	Urban	0	0	0	0
2492	Male	Youth	Urban	55.34	0.6	9.62	10.2
2493	Male	Youth	Urban	0	0	0	0
2494	Male	Youth	Urban	14.28	2.64	5.38	29.26
2495	Male	Youth	Urban	1.98	2.84	5.55	59.93
2496	Male	Youth	Urban	11.3	0.26	6.33	8.02
2497	Male	Youth	Urban	31.2	0.17	27.67	32.14
2498	Male	Youth	Urban	77.54	0.17	3.26	1.71
2499	Male	Youth	Urban	15.63	0.12	42.26	12.02
2500	Male	Youth	Urban	34.26	0.01	32.29	23.43
2501	Male	Youth	Urban	7.99	0.39	22.83	16.94
2502	Male	Youth	Urban	24.63	0.01	25.15	38.46
2503	Male	Youth	Urban	32.43	2.13	15.65	9.55
2504	Male	Youth	Urban	79.5	0.89	11.26	3.6
2505	Male	Youth	Urban	43.23	2.06	12.33	21.93
2506	Male	Youth	Urban	0	0	0	0
2507	Male	Youth	Urban	24.26	0.14	11.18	26.79

2508	Male	Youth	Urban	22.59	0.59	19.58	16.99
2509	Male	Youth	Urban	64.03	5.75	15.83	5.22
2510	Male	Youth	Urban	53.33	0.01	14.84	5.72
2511	Male	Youth	Urban	0.14	23.47	0.44	32.16
2512	Male	Youth	Urban	0	0	0	0
2513	Male	Youth	Urban	12.98	2.49	29.6	4.55
2514	Male	Youth	Urban	11.99	14.29	27.47	32.96
2515	Male	Youth	Urban	48.79	0.91	11.95	7.06
2516	Male	Youth	Urban	82.22	0.99	7.12	2.37
2517	Male	Youth	Urban	0.86	0.05	5.33	14.06
2518	Male	Youth	Urban	0	0	0	0
2519	Male	Youth	Urban	12.19	0.21	6	4.05
2520	Male	Youth	Urban	64.56	1.69	20.31	2.94
2521	Male	Youth	Urban	49.89	5.44	20.12	3.44
2522	Male	Youth	Urban	4.19	1.9	26.7	11.22
2523	Male	Youth	Urban	42.59	2.81	13.21	11.51
2524	Male	Youth	Urban	0	0	0	0
2525	Male	Youth	Urban	2.37	4.8	11.27	16.18
2526	Male	Youth	Urban	53.14	2.64	25.75	8.35
2527	Male	Youth	Urban	51.44	1.53	22.73	12.18
2528	Male	Youth	Urban	6.06	0.02	24.63	9.08
2529	Male	Youth	Urban	60.44	3.67	19.32	5.01
2530	Male	Youth	Urban	0	0	0	0
2531	Male	Youth	Urban	0	0	0	0
2532	Male	Youth	Urban	66.28	0.51	20.47	3.04
2533	Male	Youth	Urban	32.01	1.53	20.14	7.06
2534	Male	Youth	Urban	55.54	0.82	29.82	3.21
2535	Male	Youth	Urban	29.76	0.17	39.59	10.85
2536	Male	Youth	Urban	0	0	0	0
2537	Male	Youth	Urban	21.57	1.42	12.21	27.68
2538	Male	Youth	Urban	12.34	1.57	9.35	10.64
2539	Male	Youth	Urban	17.49	15.41	15.42	19.07
2540	Male	Youth	Urban	19.8	3.67	53.72	5.37
2541	Male	Youth	Urban	12.05	3.02	24.5	16.06
2542	Male	Youth	Urban	29.83	1.11	30.62	5.85
2543	Male	Youth	Urban	0	0	0	0
2544	Male	Youth	Urban	46.36	0.08	18.5	15.6
2545	Male	Youth	Urban	24.26	9.4	4.87	2.43
2546	Male	Youth	Urban	36.29	2.39	18.07	5.28
2547	Male	Youth	Urban	18.11	1.13	7.69	15.72
2548	Male	Youth	Urban	4.9	0.01	62.52	7.19
2549	Male	Youth	Urban	53.27	30.1	10.18	3.27
2550	Male	Youth	Urban	64.37	0.34	16.63	4.16
2551	Male	Youth	Urban	41.94	0.3	6.81	5.13
2552	Male	Youth	Urban	5.65	0.05	28.64	18.24
2553	Male	Youth	Urban	11.94	0.11	5.05	19.26
2554	Male	Youth	Urban	34.76	0.13	4.19	3.45
2555	Male	Youth	Urban	11.33	0.39	26.35	30.08
2556	Male	Youth	Urban	40.64	3.08	31.04	8.95
2557	Male	Youth	Urban	51.37	0.13	42.44	2.22
2558	Male	Youth	Urban	77.71	1.12	16.68	1.21
2559	Male	Youth	Urban	40.41	0	36.62	12.68
2560	Male	Youth	Urban	55.46	0.3	8.3	8.56
2561	Male	Youth	Urban	6.98	0.2	68.57	8.33
2562	Male	Youth	Urban	14.13	0.77	20.25	29.52
2563	Male	Youth	Urban	55.01	0.21	6.43	16.18
2564	Male	Youth	Urban	69.01	0.46	8.6	10.14

2565	Male	Youth	Urban	22.12	0.68	37.05	15.69
2566	Male	Youth	Urban	36.2	3.25	16.63	30.57
2567	Male	Youth	Urban	63.04	0.09	25.26	5.57
2568	Male	Youth	Urban	9.46	1.41	35.89	22.74
2569	Male	Youth	Urban	46.78	0.96	5.63	12.4
2570	Male	Youth	Urban	70.68	3.13	12.8	3.37
2571	Male	Youth	Urban	0	0	0	0
2572	Male	Youth	Urban	34.02	0.17	4.76	17.3
2573	Male	Youth	Urban	3.81	0.3	78.06	8.96
2574	Male	Youth	Urban	14.35	11.22	43.06	14.68
2575	Male	Youth	Urban	0	0	0	0
2576	Male	Youth	Urban	36.36	1.09	51.54	1.48
2577	Male	Youth	Urban	8.98	12.6	42.97	18.15
2578	Male	Youth	Urban	37.5	1.13	19.94	23.68
2579	Male	Youth	Urban	50.27	0.33	11.5	17.85
2580	Female	Middle	Urban	17.82	0.41	39.57	22.64
2581	Female	Middle	Urban	5.8	0.1	34.45	23.17
2582	Female	Middle	Urban	62.25	0.22	25.6	2.41
2583	Female	Middle	Urban	43.23	0.65	31.97	11.27
2584	Female	Middle	Urban	0	0	0	0
2585	Female	Middle	Urban	3.03	1.94	41.41	23.67
2586	Female	Middle	Urban	29.59	1.77	36.17	10.05
2587	Female	Middle	Urban	5.63	3.59	26.52	14.59
2588	Female	Middle	Urban	3.69	0.84	37.46	23.74
2589	Female	Middle	Urban	0	0	0	0
2590	Female	Middle	Urban	10.96	0.69	49.73	12.87
2591	Female	Middle	Urban	28.42	0.13	67.99	1.16
2592	Female	Middle	Urban	59.1	0.64	24.66	10.13
2593	Female	Middle	Urban	7.13	0.01	42.01	26.53
2594	Female	Middle	Urban	35.87	0.26	27.71	15.92
2595	Female	Middle	Urban	16.02	0.17	25.22	38.75
2596	Female	Middle	Urban	23.64	1.88	37.74	6.08
2597	Female	Middle	Urban	0	0	0	0
2598	Female	Middle	Urban	10.13	0.27	28.91	6.12
2599	Female	Middle	Urban	15.95	0.65	39.53	9.64
2600	Female	Middle	Urban	4.39	0.18	44.79	33.07
2601	Female	Middle	Urban	6.61	0.29	60.92	2.67
2602	Female	Middle	Urban	50.63	3.74	34.02	1.96
2603	Female	Middle	Urban	18.31	0.07	59.22	12.04
2604	Female	Middle	Urban	3.07	0.17	26.81	3.03
2605	Female	Middle	Urban	32.55	0.42	42.48	9.06
2606	Female	Middle	Urban	13.56	0.1	37.22	6.19
2607	Female	Middle	Urban	0	0	0	0
2608	Female	Toddler	Urban	0.06	3.23	51.83	14.08
2609	Female	Toddler	Urban	44.88	2.02	24.74	10.64
2610	Female	Old	Urban	27.82	0.42	47.73	4.2
2611	Female	Old	Urban	11.84	26.64	28.16	23.5
2612	Female	Old	Urban	0	0	0	0
2613	Female	Old	Urban	23.71	0.27	32.57	13.71
2614	Female	Old	Urban	6.72	0.04	69.5	6.88
2615	Female	Old	Urban	0	0	0	0
2616	Female	Old	Urban	0	0	0	0
2617	Female	Old	Urban	9.19	19.91	58.02	8.58
2618	Female	Old	Urban	22.96	2.41	41.36	12.07
2619	Female	Old	Urban	7.83	0.22	51.33	25.31
2620	Female	Old	Urban	31.51	0.39	29.07	8.47
2621	Female	Old	Urban	18.82	19.86	11.64	22.24

2622	Female	Old	Urban	7.29	0.05	24.28	34.32
2623	Female	Old	Urban	0	0	0	0
2624	Female	Youth	Urban	13.06	0.05	34.22	38.86
2625	Female	Youth	Urban	44.97	0.11	32.5	2.04
2626	Female	Youth	Urban	56.23	4.22	26.17	6.89
2627	Female	Youth	Urban	20.52	0.37	27.74	13.06
2628	Female	Youth	Urban	3.05	0.29	43.61	10.43
2629	Female	Youth	Urban	31.45	0.01	52.92	4.4
2630	Female	Youth	Urban	5.2	0.26	13.71	4.88
2631	Female	Youth	Urban	7.18	0.02	73.57	5.45
2632	Female	Youth	Urban	13.59	0.02	39.63	16.79
2633	Female	Youth	Urban	29.55	1.46	30.74	12.15
2634	Male	Middle	Urban	8.76	0.15	35.67	17.08
2635	Male	Middle	Urban	9.79	0.77	60.35	17.61
2636	Male	Middle	Urban	68.76	1.04	16.45	2.72
2637	Male	Middle	Urban	31.39	4.75	16.68	15.93
2638	Male	Middle	Urban	1.36	0.13	35.05	9.84
2639	Male	Middle	Urban	29.97	12.88	18.22	16.45
2640	Male	Middle	Urban	13.48	1.77	53.47	4.98
2641	Male	Middle	Urban	5.54	2.78	17.67	21.31
2642	Male	Middle	Urban	17.42	0.21	37.13	11
2643	Male	Middle	Urban	2.26	1.13	68.83	16.35
2644	Male	Middle	Urban	3.32	1.08	42.5	3.08
2645	Male	Middle	Urban	56.62	0.8	14.6	20.13
2646	Male	Middle	Urban	69.77	0.07	15.49	9.06
2647	Male	Middle	Urban	55.52	0.01	23.14	5.9
2648	Male	Middle	Urban	41.8	0.37	21.09	23.23
2649	Male	Middle	Urban	0	0	0	0
2650	Male	Middle	Urban	43.8	0.11	28.01	16.55
2651	Male	Middle	Urban	30.83	3.49	14.71	16.87
2652	Male	Middle	Urban	3.17	0.08	48.65	3.6
2653	Male	Middle	Urban	32.18	0.07	34.39	19.54
2654	Male	Middle	Urban	0	0	0	0
2655	Male	Middle	Urban	14.87	0.66	77.34	1.73
2656	Male	Middle	Urban	4.64	0.46	30.81	22.93
2657	Male	Middle	Urban	9.42	5.15	49	18.69
2658	Male	Middle	Urban	35.93	3.78	48.05	10.02
2659	Male	Middle	Urban	1.65	0	69.79	11.53
2660	Male	Middle	Urban	0.38	0.04	22.29	4.08
2661	Male	Middle	Urban	8.2	0.45	70.67	12.38
2662	Male	Middle	Urban	0	0	0	0
2663	Male	Toddler	Urban	74.99	0.01	22.42	1.48
2664	Male	Old	Urban	13.17	10.77	63.66	3.25
2665	Male	Old	Urban	16.85	8.11	64.53	2.5
2666	Male	Old	Urban	0	0	0	0
2667	Male	Old	Urban	4.25	3.79	45.02	3.06
2668	Male	Old	Urban	51.39	4.34	33.82	6.83
2669	Male	Old	Urban	4.23	1	57.03	9.14
2670	Male	Old	Urban	0	0	0	0
2671	Male	Old	Urban	0	0	0	0
2672	Male	Old	Urban	24.44	0.12	55.9	10.76
2673	Male	Old	Urban	9.93	0.08	44.21	4.89
2674	Male	Old	Urban	18.05	4.29	29.02	25.2
2675	Male	Old	Urban	12.55	1.09	21.04	21.39
2676	Male	Old	Urban	32.65	2.39	46.93	7.47
2677	Male	Old	Urban	11.07	68.23	11.28	2.81
2678	Male	Old	Urban	25.46	16.85	35.94	13.12

2679	Male	Old	Urban	10.08	1.09	70.62	9.38
2680	Male	Old	Urban	10.06	85.07	2.53	1.41
2681	Male	Old	Urban	6.05	0.02	76.57	9.92
2682	Male	Old	Urban	0	0	0	0
2683	Male	Old	Urban	0	0	0	0
2684	Male	Old	Urban	5.73	1.01	32.51	13.89
2685	Male	Old	Urban	0	0	0	0
2686	Male	Old	Urban	0	0	0	0
2687	Male	Old	Urban	20.04	0.12	47.31	10.83
2688	Male	Old	Urban	0	0	0	0
2689	Male	Old	Urban	0	0	0	0
2690	Male	Old	Urban	18.75	67.7	11.33	1.41
2691	Male	Old	Urban	25.01	0.27	33.69	17.18
2692	Male	Old	Urban	56.66	0.13	26.91	6.62
2693	Male	Old	Urban	8.52	42.57	31.03	7.92
2694	Male	Old	Urban	11.86	0.13	8.57	14.96
2695	Male	Old	Urban	22.99	1.44	39.37	13.46
2696	Male	Old	Urban	31.81	15.28	12.74	28.38
2697	Male	Old	Urban	15.58	0.08	35.78	38.4
2698	Male	Old	Urban	0	0	0	0
2699	Male	Old	Urban	7.94	1.73	12.52	33.14
2700	Male	Youth	Urban	41.67	0.01	29.95	22.91
2701	Male	Youth	Urban	34.65	0.09	39.33	14.74
2702	Male	Youth	Urban	73.52	0.1	9.4	2.33
2703	Male	Youth	Urban	4.25	0	40.02	22.08
2704	Male	Youth	Urban	41.13	13.5	17.77	17.94
2705	Male	Youth	Urban	6.06	0.02	24.63	9.08
2706	Male	Youth	Urban	21.59	1	66.77	3.94
2707	Male	Youth	Urban	0	0	0	0
2708	Male	Youth	Urban	0	0	0	0
2709	Male	Youth	Urban	19.47	0.13	21.6	17.86
2710	Male	Youth	Urban	14.13	1.24	11.89	7.36
2711	Male	Youth	Urban	0	0	0	0
2712	Male	Youth	Urban	0	0	0	0
2713	Male	Youth	Urban	35.73	0.56	47.5	6.26
2714	Male	Youth	Urban	8.06	0.16	6.21	43.78
2715	Male	Youth	Urban	7.61	0.6	67.67	11.73
2716	Male	Youth	Urban	48.88	0.93	25.32	13.34
2717	Male	Youth	Urban	45.63	0.54	18.32	15.29
2718	Female	Middle	Urban	19.79	0.32	45.34	8.55
2719	Female	Middle	Urban	7.98	0.03	33.73	2.6
2720	Female	Middle	Urban	11.92	1.55	36.34	7.37
2721	Female	Middle	Urban	0	0	0	0
2722	Female	Middle	Urban	1.43	0.17	58.28	7.12
2723	Female	Middle	Urban	0	0	0	0
2724	Female	Middle	Urban	7.39	12.19	41.08	28.97
2725	Female	Middle	Urban	0	0	0	0
2726	Female	Toddler	Urban	14.12	0.43	29.43	9.25
2727	Female	Toddler	Urban	22.73	0.09	8	2.02
2728	Female	Old	Urban	9.22	1.75	31.27	9.87
2729	Female	Old	Urban	4.48	67.56	10.69	0.63
2730	Female	Old	Urban	4.68	0.55	11.9	11.33
2731	Female	Youth	Urban	48.65	1.2	13.54	12.51
2732	Female	Youth	Urban	38.6	0.26	42.29	2.41
2733	Female	Youth	Urban	11.8	0.06	14.95	10.06
2734	Female	Youth	Urban	0	0	0	0
2735	Male	Middle	Urban	2.31	73.21	5.61	13.28

2736	Male	Middle	Urban	19.84	0.49	29.53	7.46
2737	Male	Middle	Urban	2.09	6.64	4.87	4.19
2738	Male	Toddler	Urban	41.77	8.1	3.27	1.9
2739	Male	Old	Urban	0	0	0	0
2740	Male	Old	Urban	6.96	28.53	12.01	16.75
2741	Male	Old	Urban	8.57	0.04	6.21	6.46
2742	Male	Old	Urban	0	0	0	0
2743	Male	Old	Urban	0	0	0	0
2744	Male	Old	Urban	0	0	0	0
2745	Male	Old	Urban	0	0	0	0
2746	Male	Youth	Urban	36.79	4.08	32.13	10
2747	Male	Youth	Urban	12.5	0.44	19.37	21.92
2748	Female	Middle	Urban	10.69	6.69	21.06	46.09
2749	Female	Middle	Urban	11.1	44.04	14.21	3
2750	Female	Middle	Urban	28.48	0.6	34.25	12.49
2751	Female	Middle	Urban	2.59	28.42	19.88	5.92
2752	Female	Middle	Urban	28.89	3.69	12.52	11.44
2753	Female	Middle	Urban	0	0	0	0
2754	Female	Middle	Urban	18.1	5.23	4.32	3.33
2755	Female	Middle	Urban	2.19	0.08	1.61	36.38
2756	Female	Middle	Urban	29.41	5.66	15.6	2.34
2757	Female	Middle	Urban	42.68	0.6	27.75	21.24
2758	Female	Middle	Urban	14.19	4.64	40.47	9.88
2759	Female	Middle	Urban	45.43	1.51	49.06	0.76
2760	Female	Middle	Urban	20.96	1.78	66.01	1.77
2761	Female	Middle	Urban	43.92	0.06	44.35	3.95
2762	Female	Middle	Urban	5.88	2.31	9.94	23.63
2763	Female	Middle	Urban	8.23	5.63	4.1	4.08
2764	Female	Middle	Urban	47.3	4.31	33.56	2.67
2765	Female	Middle	Urban	4.81	0.02	3.67	2.41
2766	Female	Middle	Urban	47.36	1.24	12.46	22.77
2767	Female	Middle	Urban	8.89	81.94	4.43	1.26
2768	Female	Middle	Urban	13.38	0.77	12.99	7.47
2769	Female	Middle	Urban	16.7	0.13	42.97	4.81
2770	Female	Middle	Urban	14.3	2.74	17.66	9.92
2771	Female	Toddler	Urban	66.87	0.15	13.03	6.9
2772	Female	Toddler	Urban	62.54	0.19	16.37	4.08
2773	Female	Toddler	Urban	68.93	0.77	13.37	2.73
2774	Female	Toddler	Urban	14.06	2.02	18.75	13.4
2775	Female	Old	Urban	4.59	0.37	39.42	21.6
2776	Female	Old	Urban	7.71	1.09	64.94	3.76
2777	Female	Old	Urban	0	0	0	0
2778	Female	Old	Urban	16.29	0.16	19.93	15.26
2779	Female	Old	Urban	29.66	4.9	17.11	22.17
2780	Female	Old	Urban	45.39	0.41	28.63	7.16
2781	Female	Youth	Urban	49.2	0.56	21.8	11.51
2782	Female	Youth	Urban	8.9	0.32	3.47	19.77
2783	Female	Youth	Urban	19.12	0.68	6.17	3.71
2784	Female	Youth	Urban	0	0	0	0
2785	Female	Youth	Urban	2.72	0.35	2.07	2.36
2786	Female	Youth	Urban	5.1	18.29	10.12	13.12
2787	Female	Youth	Urban	0	0	0	0
2788	Female	Youth	Urban	0.9	3.51	1.18	10.51
2789	Female	Youth	Urban	5.9	2.56	16.96	15.01
2790	Female	Youth	Urban	13.27	23.96	34.15	3.03
2791	Female	Youth	Urban	30.7	17.71	7.82	4.54
2792	Female	Youth	Urban	53.28	8.36	6.37	14.46

2793	Female	Youth	Urban	7.27	9.11	21.7	20.91
2794	Female	Youth	Urban	36.07	4.35	25.82	8.43
2795	Female	Youth	Urban	48.71	0.91	20.08	13.47
2796	Female	Youth	Urban	23.75	22.63	13	1.69
2797	Female	Youth	Urban	0	0	0	0
2798	Female	Youth	Urban	0	0	0	0
2799	Female	Youth	Urban	2.6	2.82	1.48	25.19
2800	Female	Youth	Urban	45.81	0.59	22.17	5.11
2801	Female	Youth	Urban	12.54	0.15	25.18	2.52
2802	Female	Youth	Urban	25.68	0.69	28.3	8.33
2803	Female	Youth	Urban	0	0	0	0
2804	Female	Youth	Urban	45.89	0.19	28.22	6.66
2805	Male	Middle	Urban	19.47	1.56	30.34	23.39
2806	Male	Middle	Urban	0	0	0	0
2807	Male	Middle	Urban	0.67	31.17	4.83	22.3
2808	Male	Middle	Urban	15.17	0.09	50.09	10.11
2809	Male	Middle	Urban	39.24	3.83	20.67	8.82
2810	Male	Middle	Urban	50.26	0.18	9.8	8.81
2811	Male	Middle	Urban	60.52	1.24	9.1	8.56
2812	Male	Middle	Urban	4.72	21.7	3.12	18.34
2813	Male	Middle	Urban	12.88	4.72	43.1	3.98
2814	Male	Middle	Urban	0	0	0	0
2815	Male	Middle	Urban	20.44	1.34	25.21	6.94
2816	Male	Middle	Urban	3.59	0.12	3.25	5.16
2817	Male	Middle	Urban	19.51	3.88	45.59	7.98
2818	Male	Middle	Urban	0	0	0	0
2819	Male	Middle	Urban	0	0	0	0
2820	Male	Middle	Urban	40.23	3.65	12.85	19.2
2821	Male	Middle	Urban	23.67	0.81	14.52	16.59
2822	Male	Middle	Urban	11.98	0.4	30.49	20.93
2823	Male	Middle	Urban	25.4	3.2	38.48	4.5
2824	Male	Middle	Urban	0	0	0	0
2825	Male	Middle	Urban	4.48	12.48	15.19	23.16
2826	Male	Middle	Urban	0	0	0	0
2827	Male	Middle	Urban	0	0	0	0
2828	Male	Middle	Urban	47.94	0.21	17.95	6.15
2829	Male	Middle	Urban	4.33	1.28	10.02	13.06
2830	Male	Middle	Urban	39.32	2.74	32.84	4.24
2831	Male	Middle	Urban	15	48.16	14.67	6.93
2832	Male	Middle	Urban	62.99	2.03	15.51	6.76
2833	Male	Middle	Urban	22.73	2.6	51.54	10.88
2834	Male	Middle	Urban	16.45	1.2	12.14	11.93
2835	Male	Middle	Urban	23.23	6.12	23.59	20.54
2836	Male	Middle	Urban	35.57	1.01	27.89	11.47
2837	Male	Middle	Urban	2.81	0.09	29.09	19.04
2838	Male	Middle	Urban	44.52	0.22	13.16	6.68
2839	Male	Middle	Urban	11.19	0.96	15.52	16.8
2840	Male	Middle	Urban	40.69	1.27	27.75	16.14
2841	Male	Middle	Urban	20.52	1.55	44.37	10.62
2842	Male	Middle	Urban	58.02	0.22	17.13	10.14
2843	Male	Toddler	Urban	2.61	7.44	20.85	38.49
2844	Male	Toddler	Urban	29.19	0.04	3.76	1.39
2845	Male	Toddler	Urban	0	0	0	0
2846	Male	Toddler	Urban	79.55	0.62	9.08	1.1
2847	Male	Toddler	Urban	50.52	4.18	25.92	4.16
2848	Male	Toddler	Urban	11.15	6.85	38.52	24.07
2849	Male	Old	Urban	35.44	0.54	23.64	18.34

2850	Male	Old	Urban	27.23	0.05	10.88	13.79
2851	Male	Old	Urban	19.24	2.12	41.91	10.68
2852	Male	Old	Urban	0	0	0	0
2853	Male	Old	Urban	17.18	0.82	39.06	24.38
2854	Male	Old	Urban	0	0	0	0
2855	Male	Old	Urban	4.57	0.05	2.33	3.7
2856	Male	Old	Urban	7.47	0.56	28.35	28.49
2857	Male	Old	Urban	7.15	34.97	18.4	18.35
2858	Male	Old	Urban	0.57	78.73	1.41	12.79
2859	Male	Old	Urban	9.49	0.15	38.07	8.41
2860	Male	Old	Urban	0	0	0	0
2861	Male	Old	Urban	9.41	0.04	7.41	17.65
2862	Male	Old	Urban	36.79	1	13.35	26.44
2863	Male	Youth	Urban	7.44	0.71	9.05	7.14
2864	Male	Youth	Urban	26.14	1.05	20.93	4.01
2865	Male	Youth	Urban	63.27	7.4	3.79	8.23
2866	Male	Youth	Urban	13.3	0.1	12.29	8.47
2867	Male	Youth	Urban	80.2	0.18	10.63	3.76
2868	Male	Youth	Urban	0	0	0	0
2869	Male	Youth	Urban	0	0	0	0
2870	Male	Youth	Urban	54.64	0.57	7.7	7.05
2871	Male	Youth	Urban	0.27	6.45	0.25	9.4
2872	Male	Youth	Urban	0	0	0	0
2873	Male	Youth	Urban	0	0	0	0
2874	Male	Youth	Urban	0	0	0	0
2875	Male	Youth	Urban	11.49	0.94	19.19	15.8
2876	Male	Youth	Urban	0	0	0	0
2877	Male	Youth	Urban	14.85	2.64	27.07	8.85
2878	Male	Youth	Urban	62.78	0.62	10.06	4.83
2879	Male	Youth	Urban	2.06	0.73	2.24	41.19
2880	Male	Youth	Urban	21.8	0.07	56.3	14.61
2881	Male	Youth	Urban	0.91	7.91	9.75	4.77
2882	Male	Youth	Urban	0	0	0	0
2883	Male	Youth	Urban	10.01	0.35	7.03	16.46
2884	Male	Youth	Urban	18.52	0.4	20.44	22.65
2885	Male	Youth	Urban	3.58	0.24	3.87	67.76
2886	Male	Youth	Urban	12.67	6.58	31.47	29.98

Number	Gender	Age	Location	Surprised	Disgusted
1	Female	Middle	Forest	0.54	9.65
2	Female	Middle	Forest	0.08	0.19
3	Female	Middle	Forest	1.29	0.11
4	Female	Middle	Forest	0.31	1.2
5	Female	Middle	Forest	1.07	0.93
6	Female	Middle	Forest	3.21	2.34
7	Female	Middle	Forest	1.56	2.04
8	Female	Middle	Forest	0.44	0.87
9	Female	Middle	Forest	0.1	6.44
10	Female	Middle	Forest	0.85	21.12
11	Female	Middle	Forest	0.74	4.82
12	Female	Middle	Forest	0.2	5.08
13	Female	Middle	Forest	0.92	0.53
14	Female	Middle	Forest	0.04	15.88
15	Female	Middle	Forest	1.47	1.22
16	Female	Middle	Forest	6.45	1.41
17	Female	Middle	Forest	0.16	0.07
18	Female	Middle	Forest	5.59	6.21
19	Female	Middle	Forest	0.47	1.76
20	Female	Middle	Forest	0	0
21	Female	Middle	Forest	5.66	0.1
22	Female	Middle	Forest	10.03	0.37
23	Female	Middle	Forest	1.53	1.96
24	Female	Middle	Forest	0.04	0
25	Female	Middle	Forest	0.23	4.59
26	Female	Middle	Forest	0.7	6.36
27	Female	Middle	Forest	9.38	1.04
28	Female	Middle	Forest	0.12	8.67
29	Female	Middle	Forest	0.58	20.61
30	Female	Middle	Forest	0.47	35.34
31	Female	Middle	Forest	1.03	5.28
32	Female	Middle	Forest	0.5	7.09
33	Female	Middle	Forest	1.35	3.43
34	Female	Middle	Forest	0.34	2.57
35	Female	Middle	Forest	0.02	10.96
36	Female	Middle	Forest	0.2	0.07
37	Female	Middle	Forest	8.03	1.28
38	Female	Middle	Forest	2.04	3.71
39	Female	Middle	Forest	0.17	69.91
40	Female	Middle	Forest	2.56	0.28
41	Female	Toddler	Forest	77.9	0.09
42	Female	Toddler	Forest	8.32	4.86
43	Female	Toddler	Forest	2.51	2.31
44	Female	Old	Forest	0	0
45	Female	Old	Forest	7.96	0.02
46	Female	Old	Forest	0.62	0.59
47	Female	Old	Forest	1.92	15.79
48	Female	Old	Forest	1.35	0.45
49	Female	Old	Forest	0.78	4.79
50	Female	Old	Forest	0.54	1.22
51	Female	Old	Forest	9.9	0.02
52	Female	Old	Forest	1.39	0.7
53	Female	Old	Forest	0.22	1.66
54	Female	Old	Forest	1.67	0.18
55	Female	Old	Forest	0.49	0.48
56	Female	Old	Forest	0	0

57	Female	Old	Forest	0.62	2.34
58	Female	Old	Forest	0.86	0.47
59	Female	Old	Forest	0.05	7.63
60	Female	Old	Forest	0.11	0.12
61	Female	Old	Forest	0.46	11.31
62	Female	Youth	Forest	5.91	2.38
63	Female	Youth	Forest	29.61	1.11
64	Female	Youth	Forest	0.72	7.85
65	Female	Youth	Forest	0.15	0.22
66	Female	Youth	Forest	0.39	39.08
67	Female	Youth	Forest	0.39	0.02
68	Female	Youth	Forest	0.02	13.51
69	Female	Youth	Forest	7.58	7.35
70	Female	Youth	Forest	0	0
71	Male	Middle	Forest	0.18	15.13
72	Male	Middle	Forest	0.19	0.88
73	Male	Middle	Forest	5.48	15.89
74	Male	Middle	Forest	1.43	0.04
75	Male	Middle	Forest	0.11	0.46
76	Male	Middle	Forest	0.19	0.82
77	Male	Middle	Forest	0.83	4.58
78	Male	Middle	Forest	21.61	2.13
79	Male	Middle	Forest	1.81	0.9
80	Male	Middle	Forest	3.04	0.68
81	Male	Middle	Forest	0.6	1.49
82	Male	Middle	Forest	2.48	4.32
83	Male	Middle	Forest	0.29	8.46
84	Male	Middle	Forest	1.97	1.62
85	Male	Middle	Forest	0.33	0.64
86	Male	Middle	Forest	0.27	0.87
87	Male	Middle	Forest	0	0
88	Male	Middle	Forest	0	0
89	Male	Middle	Forest	1.05	5.29
90	Male	Middle	Forest	2.3	1.83
91	Male	Middle	Forest	1.84	0.16
92	Male	Middle	Forest	1.23	4.97
93	Male	Middle	Forest	8.51	5.65
94	Male	Middle	Forest	0.17	3.15
95	Male	Middle	Forest	0.1	1.03
96	Male	Middle	Forest	0.21	4.27
97	Male	Middle	Forest	2.64	0.37
98	Male	Middle	Forest	0.5	1.64
99	Male	Middle	Forest	0.87	31.69
100	Male	Middle	Forest	0	0
101	Male	Middle	Forest	1.75	0.61
102	Male	Middle	Forest	0.26	1.99
103	Male	Middle	Forest	1.34	1.47
104	Male	Toddler	Forest	0.74	0.68
105	Male	Old	Forest	0	0
106	Male	Old	Forest	0.72	0.25
107	Male	Old	Forest	0.44	32.75
108	Male	Old	Forest	1.43	0.74
109	Male	Old	Forest	2.93	32.53
110	Male	Old	Forest	0	0
111	Male	Old	Forest	1.43	4.18
112	Male	Old	Forest	2.65	8.21
113	Male	Old	Forest	0.27	1.96

114	Male	Old	Forest	0.63	0.35
115	Male	Old	Forest	0.47	0.28
116	Male	Old	Forest	1.63	2.05
117	Male	Old	Forest	0	0
118	Male	Old	Forest	2.5	3.88
119	Male	Old	Forest	2.82	2.22
120	Male	Old	Forest	0.31	0.19
121	Male	Youth	Forest	9.92	3.67
122	Male	Youth	Forest	1.52	0.54
123	Male	Youth	Forest	9.26	0.14
124	Male	Youth	Forest	1.37	0.57
125	Male	Youth	Forest	1.84	0.41
126	Male	Youth	Forest	3.39	0.61
127	Male	Youth	Forest	0.44	0.35
128	Male	Youth	Forest	1.1	8.69
129	Male	Youth	Forest	0	0
130	Male	Youth	Forest	0	0
131	Male	Youth	Forest	0.22	8.3
132	Male	Youth	Forest	0.38	1.41
133	Male	Youth	Forest	0.09	4.6
134	Male	Youth	Forest	0.07	0.64
135	Male	Youth	Forest	0.69	1.64
136	Male	Youth	Forest	1.15	0.17
137	Male	Youth	Forest	0.97	15.07
138	Male	Youth	Forest	1.88	12.92
139	Male	Youth	Forest	0.37	2.16
140	Male	Youth	Forest	0.03	43.44
141	Male	Youth	Forest	11.25	4.94
142	Male	Youth	Forest	3.05	3.66
143	Female	Middle	Forest	0.1	0.83
144	Female	Middle	Forest	0.43	3.34
145	Female	Middle	Forest	1.89	31.06
146	Female	Middle	Forest	1.39	0.88
147	Female	Middle	Forest	10.41	1.83
148	Female	Middle	Forest	0.53	10.95
149	Female	Middle	Forest	0.33	0.25
150	Female	Middle	Forest	0.11	0.26
151	Female	Middle	Forest	9.78	0.17
152	Female	Middle	Forest	0.39	0.26
153	Female	Middle	Forest	7.65	6.51
154	Female	Middle	Forest	0.04	21.44
155	Female	Middle	Forest	0.22	3.42
156	Female	Middle	Forest	0.64	1.17
157	Female	Middle	Forest	0.49	0.33
158	Female	Middle	Forest	0	0
159	Female	Middle	Forest	0.95	0.1
160	Female	Middle	Forest	8.79	0.15
161	Female	Middle	Forest	0.04	59.25
162	Female	Middle	Forest	0.4	2.12
163	Female	Middle	Forest	0.05	2.24
164	Female	Middle	Forest	7.2	3.12
165	Female	Middle	Forest	1.91	1.95
166	Female	Middle	Forest	0.09	23.98
167	Female	Middle	Forest	6.33	3.38
168	Female	Middle	Forest	0.48	9.13
169	Female	Middle	Forest	11.17	0.21
170	Female	Middle	Forest	0.58	8.13

171	Female	Toddler	Forest	0	0
172	Female	Old	Forest	0.11	0.76
173	Female	Old	Forest	0.07	8.92
174	Female	Old	Forest	0	0
175	Female	Old	Forest	0.03	0.3
176	Female	Old	Forest	0	0
177	Female	Old	Forest	1.39	5.64
178	Female	Old	Forest	23.76	12.3
179	Female	Old	Forest	1.35	0.99
180	Female	Old	Forest	0.55	5.35
181	Female	Old	Forest	0.85	1.73
182	Female	Old	Forest	1.25	5.3
183	Female	Old	Forest	0	0
184	Female	Old	Forest	0.4	0.68
185	Female	Youth	Forest	9.51	2.32
186	Female	Youth	Forest	0.6	3.28
187	Female	Youth	Forest	0.77	0.02
188	Female	Youth	Forest	2.76	0.89
189	Female	Youth	Forest	1.77	7.74
190	Female	Youth	Forest	2.19	0.93
191	Female	Youth	Forest	1.22	0.74
192	Female	Youth	Forest	0.12	1.04
193	Female	Youth	Forest	3.08	0.54
194	Female	Youth	Forest	51.48	0.12
195	Female	Youth	Forest	0.13	0.03
196	Female	Youth	Forest	2.01	2.18
197	Female	Youth	Forest	0.17	0.59
198	Male	Middle	Forest	2.16	4.14
199	Male	Middle	Forest	0.56	14.84
200	Male	Middle	Forest	11.68	1.25
201	Male	Middle	Forest	3.49	7.95
202	Male	Middle	Forest	0.56	11.81
203	Male	Middle	Forest	0.52	0.7
204	Male	Middle	Forest	2.56	0.09
205	Male	Middle	Forest	2.89	0.06
206	Male	Middle	Forest	0.11	0.11
207	Male	Middle	Forest	0.16	0.56
208	Male	Middle	Forest	0.18	0.69
209	Male	Middle	Forest	0.54	4.58
210	Male	Middle	Forest	1.7	2.13
211	Male	Middle	Forest	1.04	0.59
212	Male	Middle	Forest	0.13	6.01
213	Male	Middle	Forest	0.08	6.64
214	Male	Middle	Forest	0.49	6.85
215	Male	Middle	Forest	0.73	1.42
216	Male	Toddler	Forest	1.17	0.4
217	Male	Toddler	Forest	0.42	0.14
218	Male	Toddler	Forest	1.49	7.7
219	Male	Old	Forest	0.21	4.08
220	Male	Old	Forest	0.28	0.07
221	Male	Old	Forest	1.94	0.08
222	Male	Old	Forest	0.2	1.15
223	Male	Old	Forest	1.8	0.09
224	Male	Old	Forest	0.3	0.21
225	Male	Old	Forest	0	0
226	Male	Old	Forest	2.78	2.84
227	Male	Old	Forest	0.14	0.65

228	Male	Old	Forest	0.43	3.52
229	Male	Old	Forest	5.43	0.08
230	Male	Old	Forest	3.39	0.11
231	Male	Old	Forest	0.94	0.22
232	Male	Old	Forest	0.14	31.49
233	Male	Old	Forest	0	0
234	Male	Old	Forest	0.43	5.78
235	Male	Old	Forest	0	0
236	Male	Old	Forest	0.47	7.49
237	Male	Youth	Forest	0	0
238	Male	Youth	Forest	2.1	0.19
239	Male	Youth	Forest	0.37	0.07
240	Male	Youth	Forest	1.16	0.26
241	Male	Youth	Forest	0.1	0.27
242	Male	Youth	Forest	0	0
243	Male	Youth	Forest	1.7	1.18
244	Male	Youth	Forest	7.49	2.22
245	Male	Youth	Forest	4.39	0.48
246	Male	Youth	Forest	2.55	0.68
247	Male	Youth	Forest	0.82	6.21
248	Male	Youth	Forest	3.63	1.58
249	Female	Middle	Forest	6.26	5.93
250	Female	Middle	Forest	1.33	2.14
251	Female	Middle	Forest	12.02	2.03
252	Female	Middle	Forest	0.45	1.91
253	Female	Middle	Forest	0.1	24.23
254	Female	Middle	Forest	6.69	6.36
255	Female	Middle	Forest	0.58	1.99
256	Female	Middle	Forest	0.47	2.77
257	Female	Middle	Forest	1.4	0.23
258	Female	Middle	Forest	1.92	0.42
259	Female	Middle	Forest	0.51	1.17
260	Female	Middle	Forest	0.05	9.02
261	Female	Middle	Forest	0.02	2.1
262	Female	Middle	Forest	6.39	1.32
263	Female	Middle	Forest	0.07	1.02
264	Female	Middle	Forest	0.54	20.51
265	Female	Middle	Forest	2.07	0.51
266	Female	Middle	Forest	0.06	2.43
267	Female	Middle	Forest	4.69	0.46
268	Female	Middle	Forest	0.59	0.86
269	Female	Middle	Forest	0.34	0.73
270	Female	Middle	Forest	0.89	0.15
271	Female	Middle	Forest	0.03	2.38
272	Female	Middle	Forest	0.09	9.7
273	Female	Middle	Forest	0.04	2.55
274	Female	Middle	Forest	0.11	1.24
275	Female	Middle	Forest	0.1	3.7
276	Female	Middle	Forest	1.23	0.79
277	Female	Middle	Forest	0	0
278	Female	Middle	Forest	0.5	0.06
279	Female	Middle	Forest	0.94	1.61
280	Female	Middle	Forest	0.06	2.3
281	Female	Middle	Forest	1.47	0.49
282	Female	Middle	Forest	0.39	0.5
283	Female	Middle	Forest	1.92	14.12
284	Female	Middle	Forest	11.69	1.24

285	Female	Middle	Forest	2.28	1.52
286	Female	Middle	Forest	7.81	7.11
287	Female	Middle	Forest	1.4	7.31
288	Female	Middle	Forest	1.01	0.7
289	Female	Middle	Forest	0.21	0.49
290	Female	Middle	Forest	1.33	51.68
291	Female	Middle	Forest	0.02	26.18
292	Female	Middle	Forest	0.08	3.21
293	Female	Middle	Forest	0.21	0.07
294	Female	Middle	Forest	0.09	8.94
295	Female	Middle	Forest	1.31	60.74
296	Female	Middle	Forest	0	0
297	Female	Middle	Forest	2.95	3.56
298	Female	Middle	Forest	0.37	8.99
299	Female	Middle	Forest	0.15	0.05
300	Female	Middle	Forest	0.05	0.01
301	Female	Middle	Forest	0.06	35.63
302	Female	Middle	Forest	2.14	12.57
303	Female	Middle	Forest	0.36	1.72
304	Female	Toddler	Forest	0.14	0
305	Female	Toddler	Forest	0.08	4.44
306	Female	Toddler	Forest	0.08	0.1
307	Female	Old	Forest	0	0
308	Female	Old	Forest	0.01	3.53
309	Female	Old	Forest	0.58	4.59
310	Female	Old	Forest	0.2	7.49
311	Female	Old	Forest	0.12	2.01
312	Female	Old	Forest	0.36	6.3
313	Female	Old	Forest	0.32	0.03
314	Female	Old	Forest	0.36	1.96
315	Female	Old	Forest	0.79	2.63
316	Female	Old	Forest	0.66	2.29
317	Female	Old	Forest	0.02	1.23
318	Female	Youth	Forest	0.09	0.01
319	Female	Youth	Forest	3.23	0.76
320	Female	Youth	Forest	0.29	23.98
321	Female	Youth	Forest	0.03	0.03
322	Female	Youth	Forest	0.07	0.51
323	Female	Youth	Forest	0.22	70.86
324	Female	Youth	Forest	0.02	15.83
325	Female	Youth	Forest	1.23	5.41
326	Female	Youth	Forest	0.03	9.45
327	Female	Youth	Forest	0.05	0.48
328	Female	Youth	Forest	0.24	2.81
329	Female	Youth	Forest	0.14	92.77
330	Female	Youth	Forest	2.37	1.79
331	Female	Youth	Forest	0.1	1.59
332	Female	Youth	Forest	0.02	74.86
333	Female	Youth	Forest	1.26	0.63
334	Female	Youth	Forest	3.02	9.03
335	Female	Youth	Forest	0.99	5.44
336	Female	Youth	Forest	0.12	0.18
337	Female	Youth	Forest	0.13	28.72
338	Female	Youth	Forest	1.9	2.43
339	Female	Youth	Forest	0.44	0.9
340	Female	Youth	Forest	0	0
341	Male	Middle	Forest	3.16	11.16

342	Male	Middle	Forest	0.08	3.18
343	Male	Middle	Forest	0.06	3.57
344	Male	Middle	Forest	0	0
345	Male	Middle	Forest	1.06	0.96
346	Male	Middle	Forest	1.91	0.72
347	Male	Middle	Forest	0.24	0.6
348	Male	Middle	Forest	4.05	3.17
349	Male	Middle	Forest	17.5	6.47
350	Male	Middle	Forest	0.02	3.1
351	Male	Middle	Forest	0.19	16.99
352	Male	Middle	Forest	0.07	0.07
353	Male	Middle	Forest	0.69	2.21
354	Male	Middle	Forest	0.67	35.16
355	Male	Middle	Forest	0.01	0.36
356	Male	Middle	Forest	0.47	4.62
357	Male	Middle	Forest	0.02	0.05
358	Male	Middle	Forest	2.37	6.89
359	Male	Middle	Forest	0.09	6.21
360	Male	Middle	Forest	0.18	1.5
361	Male	Middle	Forest	0.48	1.05
362	Male	Middle	Forest	0.5	32.09
363	Male	Middle	Forest	0.58	0.52
364	Male	Middle	Forest	0	0
365	Male	Middle	Forest	0.76	20.62
366	Male	Middle	Forest	0.88	17.46
367	Male	Middle	Forest	0.28	5.7
368	Male	Toddler	Forest	0.11	0.13
369	Male	Toddler	Forest	0.83	5.73
370	Male	Toddler	Forest	0.05	0.03
371	Male	Toddler	Forest	0.13	1.51
372	Male	Old	Forest	3.39	0.11
373	Male	Old	Forest	1.01	0.08
374	Male	Old	Forest	1.28	10.46
375	Male	Old	Forest	1.5	0.26
376	Male	Old	Forest	0.15	0.59
377	Male	Old	Forest	0.22	0.59
378	Male	Old	Forest	0	0
379	Male	Old	Forest	0.65	0.44
380	Male	Old	Forest	1.05	5
381	Male	Old	Forest	0	0
382	Male	Old	Forest	0.92	0.26
383	Male	Old	Forest	1.76	1.35
384	Male	Old	Forest	0.07	0.12
385	Male	Old	Forest	0.23	0.2
386	Male	Old	Forest	0.22	5.55
387	Male	Old	Forest	0.16	0.33
388	Male	Old	Forest	0.42	0.81
389	Male	Old	Forest	0.12	3.02
390	Male	Old	Forest	0.61	1.45
391	Male	Old	Forest	1.46	4.22
392	Male	Old	Forest	0.61	26.23
393	Male	Old	Forest	10.2	0.29
394	Male	Old	Forest	0.27	20.97
395	Male	Old	Forest	0.76	1.85
396	Male	Old	Forest	0.49	0.28
397	Male	Old	Forest	0.24	7.26
398	Male	Old	Forest	0.05	7.55

399	Male	Old	Forest	0.63	0.64
400	Male	Old	Forest	0.2	3.22
401	Male	Old	Forest	0.64	2.8
402	Male	Old	Forest	0.49	22.79
403	Male	Old	Forest	0.2	4.07
404	Male	Old	Forest	0.71	5.88
405	Male	Old	Forest	0.26	0.2
406	Male	Youth	Forest	0.08	1.95
407	Male	Youth	Forest	0.46	1.55
408	Male	Youth	Forest	2.27	0.05
409	Male	Youth	Forest	0.35	2.42
410	Male	Youth	Forest	1.72	17.53
411	Male	Youth	Forest	0.42	1.08
412	Male	Youth	Forest	1.4	1.48
413	Male	Youth	Forest	0.67	2.9
414	Male	Youth	Forest	0.09	0.33
415	Male	Youth	Forest	1.98	0.25
416	Male	Youth	Forest	0.12	0
417	Male	Youth	Forest	3.51	12.86
418	Male	Youth	Forest	0.5	1.97
419	Male	Youth	Forest	1.43	1.87
420	Male	Youth	Forest	1.51	41.44
421	Male	Youth	Forest	0.15	7.8
422	Male	Youth	Forest	6.21	3.93
423	Female	Middle	Forest	0.35	0.23
424	Female	Middle	Forest	0.09	0.11
425	Female	Middle	Forest	0.3	0.23
426	Female	Middle	Forest	0.52	0.11
427	Female	Middle	Forest	0	0
428	Female	Middle	Forest	0.65	0.03
429	Female	Middle	Forest	0.11	0.01
430	Female	Middle	Forest	0.35	0.04
431	Female	Middle	Forest	3.68	1.83
432	Female	Middle	Forest	0.13	40.78
433	Female	Middle	Forest	0.2	26.74
434	Female	Middle	Forest	0.32	0.23
435	Female	Middle	Forest	1.23	0.35
436	Female	Middle	Forest	0.1	0.13
437	Female	Middle	Forest	0.19	6.12
438	Female	Middle	Forest	0.35	0.02
439	Female	Middle	Forest	0.12	3.18
440	Female	Middle	Forest	0.13	3.61
441	Female	Middle	Forest	2.49	1.84
442	Female	Middle	Forest	2.47	0.26
443	Female	Middle	Forest	0.37	0.69
444	Female	Middle	Forest	0.12	0.55
445	Female	Middle	Forest	8.83	16.67
446	Female	Middle	Forest	1.56	0.35
447	Female	Middle	Forest	0.18	0.18
448	Female	Middle	Forest	0.48	1.72
449	Female	Middle	Forest	0.82	13.03
450	Female	Middle	Forest	0.92	0.36
451	Female	Middle	Forest	0.36	0.38
452	Female	Middle	Forest	0.06	0.01
453	Female	Middle	Forest	0.16	4.11
454	Female	Middle	Forest	3.89	0.28
455	Female	Middle	Forest	1.98	1.92

456	Female	Middle	Forest	0	0
457	Female	Middle	Forest	0.06	0.09
458	Female	Middle	Forest	1.07	1.07
459	Female	Middle	Forest	0.08	1.23
460	Female	Middle	Forest	0.18	0
461	Female	Middle	Forest	7.27	6.48
462	Female	Middle	Forest	0.12	0.02
463	Female	Middle	Forest	0.05	0.04
464	Female	Middle	Forest	0	0
465	Female	Middle	Forest	0.04	1.1
466	Female	Middle	Forest	4.81	5.1
467	Female	Middle	Forest	0.11	0.02
468	Female	Middle	Forest	0.04	0.78
469	Female	Middle	Forest	0.61	0.41
470	Female	Middle	Forest	0	0.01
471	Female	Middle	Forest	0.43	0.14
472	Female	Middle	Forest	0.26	0.16
473	Female	Middle	Forest	0.09	0.01
474	Female	Middle	Forest	0.36	0.21
475	Female	Toddler	Forest	0.03	7.01
476	Female	Toddler	Forest	0	0
477	Female	Toddler	Forest	0.15	0.19
478	Female	Toddler	Forest	0.02	0
479	Female	Toddler	Forest	0.22	0.03
480	Female	Toddler	Forest	0.1	1.02
481	Female	Toddler	Forest	1.12	1.03
482	Female	Toddler	Forest	0.12	0.17
483	Female	Toddler	Forest	1.31	0.24
484	Female	Toddler	Forest	1.18	5.09
485	Female	Toddler	Forest	0.13	35.5
486	Female	Toddler	Forest	0.77	0.32
487	Female	Old	Forest	0.03	1.03
488	Female	Old	Forest	0.44	2.63
489	Female	Old	Forest	0.07	0.03
490	Female	Old	Forest	0.19	2.93
491	Female	Old	Forest	0.32	2.53
492	Female	Old	Forest	0	0
493	Female	Old	Forest	0.04	0.41
494	Female	Old	Forest	0	0
495	Female	Old	Forest	0.05	0.08
496	Female	Old	Forest	0.52	4.36
497	Female	Old	Forest	1.07	0.07
498	Female	Old	Forest	0	0
499	Female	Old	Forest	0.02	0.22
500	Female	Old	Forest	0	0
501	Female	Old	Forest	0.05	0.78
502	Female	Old	Forest	0.16	1.04
503	Female	Old	Forest	0.16	16.13
504	Female	Old	Forest	1.22	0.3
505	Female	Old	Forest	0.04	0.01
506	Female	Old	Forest	2.16	0.03
507	Female	Old	Forest	0.34	0.8
508	Female	Old	Forest	0.06	0.55
509	Female	Old	Forest	0.01	0
510	Female	Old	Forest	0.73	6.31
511	Female	Old	Forest	0.25	1.91
512	Female	Old	Forest	0.31	0.01

513	Female	Old	Forest	2.24	1.07
514	Female	Old	Forest	2.4	0.01
515	Female	Old	Forest	0.06	0.63
516	Female	Old	Forest	0.04	0.32
517	Female	Old	Forest	0.16	0.07
518	Female	Old	Forest	0.13	0.01
519	Female	Old	Forest	0.58	0.41
520	Female	Old	Forest	1.77	0.09
521	Female	Old	Forest	0.43	1.52
522	Female	Old	Forest	0.07	0.01
523	Female	Old	Forest	0.54	1.88
524	Female	Old	Forest	0.48	0
525	Female	Old	Forest	0.26	0.89
526	Female	Old	Forest	0.15	20.71
527	Female	Old	Forest	2.99	1.26
528	Female	Youth	Forest	4.46	0.2
529	Female	Youth	Forest	0.3	0.51
530	Female	Youth	Forest	0.82	0.02
531	Female	Youth	Forest	0.71	1.66
532	Female	Youth	Forest	0.21	0.63
533	Female	Youth	Forest	0.05	0
534	Female	Youth	Forest	0	0
535	Female	Youth	Forest	0.06	0
536	Female	Youth	Forest	0.81	0.22
537	Female	Youth	Forest	0.1	0.01
538	Female	Youth	Forest	0.09	0.76
539	Female	Youth	Forest	0.04	3.41
540	Female	Youth	Forest	0.82	0.05
541	Female	Youth	Forest	0.79	0.18
542	Female	Youth	Forest	5.98	0.24
543	Female	Youth	Forest	0.22	1.67
544	Female	Youth	Forest	0	0
545	Female	Youth	Forest	0.04	2.71
546	Female	Youth	Forest	8.07	0.74
547	Female	Youth	Forest	0.05	0.09
548	Female	Youth	Forest	0.11	0.01
549	Female	Youth	Forest	2.4	25.17
550	Female	Youth	Forest	1.79	1.9
551	Female	Youth	Forest	13.53	0.8
552	Female	Youth	Forest	0.07	0
553	Female	Youth	Forest	0.5	3.37
554	Female	Youth	Forest	0.11	0.02
555	Female	Youth	Forest	0.2	3.44
556	Female	Youth	Forest	1.73	0.02
557	Female	Youth	Forest	0.26	4.94
558	Female	Youth	Forest	0.72	1.24
559	Female	Youth	Forest	14.81	2.6
560	Female	Youth	Forest	0.02	0
561	Female	Youth	Forest	2.49	0.13
562	Female	Youth	Forest	1.41	0.58
563	Female	Youth	Forest	0.89	4.52
564	Female	Youth	Forest	0.06	0.63
565	Female	Youth	Forest	0.06	0.07
566	Female	Youth	Forest	0.18	0.03
567	Female	Youth	Forest	11.22	0.29
568	Female	Youth	Forest	0.45	6.03
569	Female	Youth	Forest	0.68	4.1

570	Female	Youth	Forest	0.03	6.14
571	Female	Youth	Forest	1.13	26.19
572	Female	Youth	Forest	0.05	0.02
573	Female	Youth	Forest	1.53	0.75
574	Female	Youth	Forest	0.07	0.11
575	Female	Youth	Forest	0.69	5.2
576	Female	Youth	Forest	0.99	0.09
577	Female	Youth	Forest	1.78	5.29
578	Female	Youth	Forest	0.23	5.29
579	Female	Youth	Forest	0.63	2.35
580	Female	Youth	Forest	0.05	0.32
581	Female	Youth	Forest	0.69	0.13
582	Female	Youth	Forest	2	0.41
583	Female	Youth	Forest	0	0
584	Female	Youth	Forest	0.08	0.25
585	Female	Youth	Forest	2.42	4.93
586	Female	Youth	Forest	1.78	0.45
587	Female	Youth	Forest	0.32	0.01
588	Female	Youth	Forest	4.26	0.25
589	Female	Youth	Forest	1.84	14.58
590	Female	Youth	Forest	0.05	0
591	Female	Youth	Forest	0.03	0.02
592	Female	Youth	Forest	26.4	0.51
593	Female	Youth	Forest	0.24	3.19
594	Female	Youth	Forest	4.81	4.53
595	Female	Youth	Forest	0	0
596	Female	Youth	Forest	0.02	0
597	Female	Youth	Forest	0.99	0.18
598	Female	Youth	Forest	0.03	0
599	Female	Youth	Forest	0.42	1.18
600	Female	Youth	Forest	0.22	1.49
601	Female	Youth	Forest	0.06	7.11
602	Female	Youth	Forest	0.53	0.27
603	Female	Youth	Forest	0.02	0
604	Female	Youth	Forest	1.51	6.46
605	Female	Youth	Forest	1.79	0.13
606	Female	Youth	Forest	1.48	2.97
607	Female	Youth	Forest	8.95	0.69
608	Female	Youth	Forest	0.37	0.74
609	Female	Youth	Forest	0.67	1.56
610	Female	Youth	Forest	0.52	2.38
611	Male	Middle	Forest	0	0
612	Male	Middle	Forest	3.09	2.31
613	Male	Middle	Forest	0.74	1.49
614	Male	Middle	Forest	0.01	0.72
615	Male	Middle	Forest	0.24	0.38
616	Male	Middle	Forest	0.01	0.02
617	Male	Middle	Forest	3.9	1.68
618	Male	Middle	Forest	0.12	0.14
619	Male	Middle	Forest	0.65	1.12
620	Male	Middle	Forest	0.42	0.12
621	Male	Middle	Forest	0.43	1.05
622	Male	Middle	Forest	0.19	0.02
623	Male	Middle	Forest	0.12	10.09
624	Male	Middle	Forest	0.13	0
625	Male	Middle	Forest	1.27	2.67
626	Male	Middle	Forest	1.51	1.31

627	Male	Middle	Forest	0.16	0.12
628	Male	Middle	Forest	0.27	0.01
629	Male	Middle	Forest	0.24	0.11
630	Male	Middle	Forest	0.26	0.28
631	Male	Middle	Forest	0.41	0.23
632	Male	Middle	Forest	1.36	5.8
633	Male	Middle	Forest	4.25	0.13
634	Male	Middle	Forest	1	2.25
635	Male	Middle	Forest	0.7	5.15
636	Male	Middle	Forest	0.04	0
637	Male	Middle	Forest	0.34	5.9
638	Male	Middle	Forest	0.35	20.65
639	Male	Middle	Forest	1.95	1.18
640	Male	Middle	Forest	2.72	0.33
641	Male	Middle	Forest	2.34	1.35
642	Male	Middle	Forest	0.44	2.38
643	Male	Middle	Forest	2.43	1.68
644	Male	Middle	Forest	0.04	0.05
645	Male	Middle	Forest	2.81	1.03
646	Male	Middle	Forest	0.52	0.98
647	Male	Middle	Forest	0.02	0.04
648	Male	Middle	Forest	0.23	3.94
649	Male	Toddler	Forest	9.12	0.16
650	Male	Toddler	Forest	0.24	0.3
651	Male	Toddler	Forest	0.22	0.25
652	Male	Toddler	Forest	0.5	0.26
653	Male	Toddler	Forest	0	0
654	Male	Toddler	Forest	0.6	1.14
655	Male	Toddler	Forest	7.07	4.76
656	Male	Toddler	Forest	0.14	0
657	Male	Toddler	Forest	0.12	0
658	Male	Old	Forest	0.23	0.65
659	Male	Old	Forest	0.35	1.18
660	Male	Old	Forest	0.39	0.12
661	Male	Old	Forest	9.08	0.12
662	Male	Old	Forest	0.1	0.51
663	Male	Old	Forest	0.26	4.62
664	Male	Old	Forest	2.25	0.46
665	Male	Old	Forest	0	0
666	Male	Old	Forest	2.16	0.2
667	Male	Old	Forest	0.2	0.18
668	Male	Old	Forest	0.04	0
669	Male	Old	Forest	3.64	4.13
670	Male	Old	Forest	2.78	0.99
671	Male	Old	Forest	0	0
672	Male	Old	Forest	0.14	0.27
673	Male	Old	Forest	0.1	1.43
674	Male	Old	Forest	0.98	0.13
675	Male	Old	Forest	0	0
676	Male	Old	Forest	0.46	6.74
677	Male	Old	Forest	0	0
678	Male	Youth	Forest	7.76	2.42
679	Male	Youth	Forest	0.6	1.79
680	Male	Youth	Forest	0.95	0
681	Male	Youth	Forest	0.76	0.06
682	Male	Youth	Forest	3.26	0.13
683	Male	Youth	Forest	0.02	0.16

684	Male	Youth	Forest	2.6	1.53
685	Male	Youth	Forest	0.03	0.18
686	Male	Youth	Forest	0.18	5.82
687	Male	Youth	Forest	0.29	3.51
688	Male	Youth	Forest	15.84	0.03
689	Male	Youth	Forest	0.02	0
690	Male	Youth	Forest	10.58	0.63
691	Male	Youth	Forest	1.73	2.46
692	Male	Youth	Forest	0.24	0.54
693	Male	Youth	Forest	4.27	0.11
694	Male	Youth	Forest	0.04	0.2
695	Male	Youth	Forest	0	0
696	Male	Youth	Forest	0.23	0.3
697	Male	Youth	Forest	0.67	1.99
698	Male	Youth	Forest	3.02	0.01
699	Male	Youth	Forest	0.04	0.02
700	Male	Youth	Forest	0.17	0.63
701	Male	Youth	Forest	1.22	15.11
702	Male	Youth	Forest	0	0
703	Male	Youth	Forest	0.2	0
704	Male	Youth	Forest	0	0
705	Male	Youth	Forest	0	0
706	Male	Youth	Forest	0.47	0.05
707	Male	Youth	Forest	0.49	0.22
708	Male	Youth	Forest	0.05	0.02
709	Male	Youth	Forest	3.49	10.72
710	Male	Youth	Forest	0.45	1.77
711	Male	Youth	Forest	3.49	17.44
712	Male	Youth	Forest	0.68	0.11
713	Male	Youth	Forest	1.88	0.71
714	Male	Youth	Forest	0.16	0.09
715	Male	Youth	Forest	0	0
716	Male	Youth	Forest	9.83	0.25
717	Male	Youth	Forest	0.09	0.01
718	Male	Youth	Forest	0.17	1.04
719	Male	Youth	Forest	2.7	5.69
720	Male	Youth	Forest	2.72	0.33
721	Male	Youth	Forest	7.43	0.02
722	Male	Youth	Forest	3.83	3.25
723	Male	Youth	Forest	0.22	0.17
724	Male	Youth	Forest	6.31	1.38
725	Male	Youth	Forest	2.02	50.44
726	Male	Youth	Forest	6.27	1.31
727	Male	Youth	Forest	10.02	23.92
728	Male	Youth	Forest	0.16	0.07
729	Male	Youth	Forest	0.07	0.05
730	Male	Youth	Forest	0.71	0.47
731	Male	Youth	Forest	0.82	3.59
732	Male	Youth	Forest	1.04	1.05
733	Male	Youth	Forest	0.05	0.08
734	Male	Youth	Forest	5.72	7.35
735	Male	Youth	Forest	0.08	0.1
736	Male	Youth	Forest	0	0
737	Male	Youth	Forest	0.3	1.55
738	Male	Youth	Forest	3.31	10.7
739	Male	Youth	Forest	1.14	0.29
740	Male	Youth	Forest	2.08	13.25

741	Male	Youth	Forest	0.13	0.02
742	Male	Youth	Forest	0.1	0
743	Male	Youth	Forest	1.75	27.95
744	Male	Youth	Forest	0.12	12.9
745	Male	Youth	Forest	0.14	0.16
746	Male	Youth	Forest	0.21	0.14
747	Male	Youth	Forest	0.32	0.63
748	Male	Youth	Forest	0.05	0.01
749	Male	Youth	Forest	0.16	0.06
750	Male	Youth	Forest	4.27	0.77
751	Male	Youth	Forest	8.61	3.77
752	Male	Youth	Forest	0.04	0
753	Male	Youth	Forest	0.02	0.05
754	Male	Youth	Forest	0.18	0
755	Male	Youth	Forest	0.01	0.01
756	Male	Youth	Forest	4.32	3.58
757	Male	Youth	Forest	1.98	2.8
758	Male	Youth	Forest	0.83	0.56
759	Male	Youth	Forest	0.26	1.1
760	Male	Youth	Forest	2.35	0.31
761	Male	Youth	Forest	0.04	1.41
762	Male	Youth	Forest	1.52	14.55
763	Male	Youth	Forest	1.25	0.07
764	Male	Youth	Forest	0.43	3.02
765	Female	Middle	Forest	0.34	12.8
766	Female	Middle	Forest	0.42	0.47
767	Female	Middle	Forest	0.17	0.36
768	Female	Middle	Forest	0.06	4.15
769	Female	Middle	Forest	3.87	5.93
770	Female	Middle	Forest	0.46	22.73
771	Female	Middle	Forest	1.08	12.85
772	Female	Middle	Forest	0.12	2.25
773	Female	Middle	Forest	0.41	0.72
774	Female	Middle	Forest	0.35	15.74
775	Female	Middle	Forest	10.23	0.02
776	Female	Middle	Forest	0.43	0.72
777	Female	Middle	Forest	0	0
778	Female	Middle	Forest	1.06	0.46
779	Female	Middle	Forest	1.84	2.8
780	Female	Middle	Forest	0.03	0.43
781	Female	Middle	Forest	0.12	6.56
782	Female	Middle	Forest	0.06	0.15
783	Female	Middle	Forest	1.82	6.7
784	Female	Middle	Forest	0.74	0.03
785	Female	Middle	Forest	1.33	6.49
786	Female	Middle	Forest	7.5	10.57
787	Female	Middle	Forest	0.32	1.79
788	Female	Middle	Forest	2.4	11.04
789	Female	Middle	Forest	0.44	0.07
790	Female	Middle	Forest	0.18	0.56
791	Female	Middle	Forest	2.59	0.12
792	Female	Middle	Forest	0.17	0.32
793	Female	Middle	Forest	3.73	2.84
794	Female	Middle	Forest	0.01	0.01
795	Female	Middle	Forest	2.45	0.17
796	Female	Middle	Forest	0.8	0.56
797	Female	Middle	Forest	2.91	1.4

798	Female	Middle	Forest	2.31	1.71
799	Female	Middle	Forest	0.14	0.71
800	Female	Middle	Forest	0.18	0.16
801	Female	Middle	Forest	0.43	0.04
802	Female	Middle	Forest	1.48	0.1
803	Female	Middle	Forest	36.69	1.13
804	Female	Middle	Forest	0.19	0.22
805	Female	Middle	Forest	3.94	0.09
806	Female	Middle	Forest	0.23	0.25
807	Female	Middle	Forest	0	0
808	Female	Middle	Forest	5.89	8.56
809	Female	Middle	Forest	1.7	0.39
810	Female	Middle	Forest	0.39	2.39
811	Female	Middle	Forest	0.31	1.03
812	Female	Middle	Forest	0.05	4.66
813	Female	Middle	Forest	1.14	1.95
814	Female	Middle	Forest	1.64	14.47
815	Female	Middle	Forest	0.46	2.28
816	Female	Middle	Forest	0.17	0.75
817	Female	Middle	Forest	0.01	0.01
818	Female	Middle	Forest	22.99	7.41
819	Female	Middle	Forest	3.55	0.01
820	Female	Middle	Forest	4.38	7.64
821	Female	Middle	Forest	1.45	0.06
822	Female	Middle	Forest	0.03	9.23
823	Female	Middle	Forest	13.44	1.3
824	Female	Middle	Forest	0.03	0.11
825	Female	Middle	Forest	1.4	3.24
826	Female	Middle	Forest	0.28	0.93
827	Female	Middle	Forest	1.39	0.12
828	Female	Middle	Forest	2.16	1.74
829	Female	Middle	Forest	0.06	0.72
830	Female	Middle	Forest	0.41	1.23
831	Female	Middle	Forest	9.81	19.49
832	Female	Middle	Forest	0.95	0.24
833	Female	Middle	Forest	1.18	6.13
834	Female	Middle	Forest	0.01	0.34
835	Female	Middle	Forest	0.13	2.64
836	Female	Middle	Forest	0.34	0.92
837	Female	Middle	Forest	0.15	12.13
838	Female	Middle	Forest	1.5	0.02
839	Female	Middle	Forest	0	0
840	Female	Middle	Forest	0.23	0.72
841	Female	Middle	Forest	0.24	0.19
842	Female	Middle	Forest	0.43	8.19
843	Female	Middle	Forest	0	0
844	Female	Middle	Forest	0.06	2.02
845	Female	Middle	Forest	0.05	5.39
846	Female	Middle	Forest	0.18	1.27
847	Female	Middle	Forest	11.81	0.57
848	Female	Middle	Forest	0.29	2.9
849	Female	Middle	Forest	12.39	0.62
850	Female	Middle	Forest	0	0
851	Female	Middle	Forest	10.72	2.23
852	Female	Middle	Forest	9.01	8.82
853	Female	Middle	Forest	3.14	2.39
854	Female	Middle	Forest	0.05	4.75

855	Female	Middle	Forest	0.27	0.19
856	Female	Middle	Forest	0.21	1.5
857	Female	Middle	Forest	0.27	4.36
858	Female	Middle	Forest	0.54	5.69
859	Female	Middle	Forest	0.13	0.78
860	Female	Middle	Forest	2.93	19.32
861	Female	Middle	Forest	2.19	0.3
862	Female	Middle	Forest	9.21	10.36
863	Female	Toddler	Forest	0.43	0.81
864	Female	Toddler	Forest	0.42	1.19
865	Female	Toddler	Forest	3.44	4.86
866	Female	Toddler	Forest	2.36	0.39
867	Female	Toddler	Forest	0.14	0.51
868	Female	Toddler	Forest	0.04	0.56
869	Female	Toddler	Forest	0.03	0.78
870	Female	Toddler	Forest	0.26	1.08
871	Female	Toddler	Forest	20.25	1
872	Female	Toddler	Forest	0.96	0.57
873	Female	Toddler	Forest	0.43	0.66
874	Female	Toddler	Forest	4.98	0
875	Female	Toddler	Forest	0.44	0.92
876	Female	Toddler	Forest	2.14	1.61
877	Female	Toddler	Forest	0.02	0.03
878	Female	Toddler	Forest	0	0
879	Female	Toddler	Forest	0.49	0.13
880	Female	Toddler	Forest	0.32	0.22
881	Female	Toddler	Forest	0	0
882	Female	Toddler	Forest	9.63	0.08
883	Female	Toddler	Forest	0.62	0.1
884	Female	Toddler	Forest	1.95	0.2
885	Female	Toddler	Forest	0.86	11.12
886	Female	Toddler	Forest	0.05	1.22
887	Female	Toddler	Forest	0.44	0.91
888	Female	Toddler	Forest	0.04	0
889	Female	Toddler	Forest	1.37	0.5
890	Female	Toddler	Forest	0	0
891	Female	Old	Forest	0.42	0.01
892	Female	Old	Forest	0.03	0.01
893	Female	Old	Forest	0.17	0.2
894	Female	Old	Forest	0.03	0.58
895	Female	Old	Forest	0	0
896	Female	Old	Forest	0.81	0.1
897	Female	Old	Forest	0.39	0.61
898	Female	Old	Forest	2.54	0.21
899	Female	Old	Forest	1.64	1.38
900	Female	Old	Forest	0.04	4.37
901	Female	Old	Forest	0.05	0.08
902	Female	Old	Forest	0.31	0.31
903	Female	Old	Forest	3.27	0.42
904	Female	Old	Forest	0.47	0.24
905	Female	Old	Forest	5.29	3.67
906	Female	Old	Forest	0	0
907	Female	Old	Forest	0.4	0.1
908	Female	Old	Forest	1.18	0.37
909	Female	Old	Forest	0.16	0.62
910	Female	Old	Forest	0.09	0.27
911	Female	Old	Forest	1.48	0.41

912	Female	Old	Forest	0.43	0.4
913	Female	Old	Forest	0.26	0.82
914	Female	Old	Forest	3.26	1.5
915	Female	Old	Forest	0	0
916	Female	Old	Forest	0.03	0
917	Female	Old	Forest	0.31	0.38
918	Female	Old	Forest	0.94	2.53
919	Female	Old	Forest	0	0
920	Female	Old	Forest	3.07	2.43
921	Female	Old	Forest	0.09	0.05
922	Female	Old	Forest	0.25	0
923	Female	Old	Forest	1.02	0.04
924	Female	Old	Forest	2.84	0.21
925	Female	Old	Forest	0.32	1.14
926	Female	Old	Forest	1.66	0.88
927	Female	Old	Forest	1.02	0.18
928	Female	Old	Forest	1.17	4.76
929	Female	Old	Forest	2.19	3.56
930	Female	Old	Forest	0.56	2.8
931	Female	Old	Forest	0.38	0.19
932	Female	Old	Forest	0.18	1.62
933	Female	Old	Forest	1.11	1.95
934	Female	Old	Forest	0.21	10.57
935	Female	Old	Forest	0.28	2.92
936	Female	Old	Forest	0.2	0.32
937	Female	Old	Forest	4.39	0.52
938	Female	Old	Forest	0	0
939	Female	Old	Forest	5.09	6.11
940	Female	Youth	Forest	4.78	0.82
941	Female	Youth	Forest	0	0
942	Female	Youth	Forest	0.67	0.91
943	Female	Youth	Forest	0.53	0.2
944	Female	Youth	Forest	0.5	6.62
945	Female	Youth	Forest	0.92	1.11
946	Female	Youth	Forest	0.07	11.22
947	Female	Youth	Forest	2.63	2.14
948	Female	Youth	Forest	13.53	5.62
949	Female	Youth	Forest	0.3	0.11
950	Female	Youth	Forest	0.15	0.08
951	Female	Youth	Forest	0.25	0.1
952	Female	Youth	Forest	0.37	1.55
953	Female	Youth	Forest	0.09	0.34
954	Female	Youth	Forest	0.03	0.12
955	Female	Youth	Forest	1.33	8.78
956	Female	Youth	Forest	0.04	0
957	Female	Youth	Forest	3.63	0.34
958	Female	Youth	Forest	10.81	0.26
959	Female	Youth	Forest	0.46	9.73
960	Female	Youth	Forest	2.79	0.73
961	Female	Youth	Forest	0	0
962	Female	Youth	Forest	0.03	0.51
963	Female	Youth	Forest	0.08	0.31
964	Female	Youth	Forest	0.93	1.32
965	Female	Youth	Forest	1.19	0.09
966	Female	Youth	Forest	0.07	1.62
967	Female	Youth	Forest	0.08	0.07
968	Female	Youth	Forest	0.55	0.78

969	Female	Youth	Forest	0.17	1.03
970	Female	Youth	Forest	4.08	4.96
971	Female	Youth	Forest	0.17	0.07
972	Female	Youth	Forest	9.8	0.1
973	Female	Youth	Forest	0.41	0.19
974	Female	Youth	Forest	0.12	3.23
975	Female	Youth	Forest	9.82	0.52
976	Female	Youth	Forest	0.75	0.71
977	Female	Youth	Forest	2.93	33.59
978	Female	Youth	Forest	5.82	0.51
979	Female	Youth	Forest	0.53	37.54
980	Female	Youth	Forest	2.47	6.65
981	Female	Youth	Forest	0.05	1.98
982	Female	Youth	Forest	0.1	0.54
983	Female	Youth	Forest	0.06	0.53
984	Female	Youth	Forest	22.53	0.03
985	Female	Youth	Forest	1.5	1.35
986	Female	Youth	Forest	0.23	0.03
987	Female	Youth	Forest	1.01	1.01
988	Female	Youth	Forest	1.23	0.19
989	Female	Youth	Forest	3.21	0.55
990	Female	Youth	Forest	8.29	0.43
991	Female	Youth	Forest	0.28	1.85
992	Female	Youth	Forest	0.03	1.05
993	Female	Youth	Forest	0.49	1.51
994	Female	Youth	Forest	0.7	4.03
995	Female	Youth	Forest	5.16	12.7
996	Female	Youth	Forest	0.17	0.97
997	Female	Youth	Forest	0.04	0.14
998	Female	Youth	Forest	0.24	0.16
999	Female	Youth	Forest	0.69	1.57
1000	Female	Youth	Forest	0	0
1001	Female	Youth	Forest	5.53	0.99
1002	Female	Youth	Forest	0.78	0.58
1003	Female	Youth	Forest	0.26	0.86
1004	Female	Youth	Forest	0.51	0.14
1005	Female	Youth	Forest	30.3	0.31
1006	Female	Youth	Forest	5.68	4.33
1007	Female	Youth	Forest	54.92	0.2
1008	Female	Youth	Forest	0.02	0.35
1009	Female	Youth	Forest	0.62	13.8
1010	Female	Youth	Forest	1.44	0.08
1011	Female	Youth	Forest	0	0
1012	Female	Youth	Forest	0.43	2.64
1013	Female	Youth	Forest	0.53	0.06
1014	Female	Youth	Forest	0.05	0.68
1015	Female	Youth	Forest	1.08	1.21
1016	Female	Youth	Forest	0.37	0.18
1017	Female	Youth	Forest	5.85	6.44
1018	Female	Youth	Forest	20.87	0.42
1019	Female	Youth	Forest	2.33	2.18
1020	Female	Youth	Forest	0.2	0.26
1021	Female	Youth	Forest	4.24	0.01
1022	Female	Youth	Forest	1.09	8.68
1023	Female	Youth	Forest	0.61	2.48
1024	Female	Youth	Forest	12.88	0.72
1025	Female	Youth	Forest	0.88	6.61

1026	Female	Youth	Forest	5.49	0.63
1027	Female	Youth	Forest	0.66	0.05
1028	Female	Youth	Forest	0.67	0.37
1029	Female	Youth	Forest	0.29	3.42
1030	Female	Youth	Forest	23.06	4.3
1031	Male	Middle	Forest	0.46	0.03
1032	Male	Middle	Forest	0.17	1.22
1033	Male	Middle	Forest	0.92	0.04
1034	Male	Middle	Forest	10.24	0.19
1035	Male	Middle	Forest	0	0
1036	Male	Middle	Forest	0.41	0.21
1037	Male	Middle	Forest	0.84	0.3
1038	Male	Middle	Forest	0.27	1.53
1039	Male	Middle	Forest	0.66	0.65
1040	Male	Middle	Forest	0.38	3.32
1041	Male	Middle	Forest	0	0
1042	Male	Middle	Forest	0.13	0.23
1043	Male	Middle	Forest	1	0.62
1044	Male	Middle	Forest	0	0
1045	Male	Middle	Forest	8.66	1.33
1046	Male	Middle	Forest	4.08	0.26
1047	Male	Middle	Forest	0.03	0.02
1048	Male	Middle	Forest	1.52	0.23
1049	Male	Middle	Forest	0.15	3.13
1050	Male	Middle	Forest	10.34	2.94
1051	Male	Middle	Forest	0.16	0.5
1052	Male	Middle	Forest	0.13	0.17
1053	Male	Middle	Forest	0.14	0.06
1054	Male	Middle	Forest	0.17	0.33
1055	Male	Middle	Forest	0.12	0.43
1056	Male	Middle	Forest	0.06	9.25
1057	Male	Middle	Forest	0.15	3.45
1058	Male	Middle	Forest	0.59	6
1059	Male	Middle	Forest	0.35	6.08
1060	Male	Middle	Forest	0.19	0.04
1061	Male	Middle	Forest	2.09	2.77
1062	Male	Middle	Forest	3.54	0.56
1063	Male	Middle	Forest	0.43	0.27
1064	Male	Middle	Forest	0.07	1.4
1065	Male	Middle	Forest	1.74	0.64
1066	Male	Middle	Forest	0.51	1.64
1067	Male	Middle	Forest	32.66	0.29
1068	Male	Middle	Forest	0.27	17.25
1069	Male	Middle	Forest	0.48	0.6
1070	Male	Middle	Forest	0.25	0.85
1071	Male	Middle	Forest	6.4	2.25
1072	Male	Middle	Forest	1.21	1
1073	Male	Middle	Forest	0	0
1074	Male	Middle	Forest	0.83	0.38
1075	Male	Middle	Forest	5.2	0.08
1076	Male	Middle	Forest	4.68	0.77
1077	Male	Middle	Forest	1.57	0.21
1078	Male	Middle	Forest	17.11	0.07
1079	Male	Middle	Forest	4.06	10.81
1080	Male	Middle	Forest	0.57	2.89
1081	Male	Middle	Forest	0.36	1.03
1082	Male	Middle	Forest	4.18	13.63

1083	Male	Toddler	Forest	0	0
1084	Male	Toddler	Forest	0.21	0.05
1085	Male	Toddler	Forest	0.86	0.23
1086	Male	Toddler	Forest	0.61	0.03
1087	Male	Toddler	Forest	0.56	0.72
1088	Male	Toddler	Forest	0	0
1089	Male	Toddler	Forest	0.36	0.44
1090	Male	Toddler	Forest	0	0
1091	Male	Toddler	Forest	0.03	0.2
1092	Male	Toddler	Forest	1.28	1.32
1093	Male	Toddler	Forest	0.46	0.09
1094	Male	Toddler	Forest	1.93	2.02
1095	Male	Toddler	Forest	0	0
1096	Male	Toddler	Forest	0	0
1097	Male	Toddler	Forest	0	0
1098	Male	Toddler	Forest	2.86	0.08
1099	Male	Toddler	Forest	0	0
1100	Male	Toddler	Forest	18.33	0.16
1101	Male	Toddler	Forest	0.05	0.32
1102	Male	Toddler	Forest	0.16	0.16
1103	Male	Toddler	Forest	0.09	0.03
1104	Male	Toddler	Forest	0.34	0.64
1105	Male	Toddler	Forest	3.15	3.12
1106	Male	Toddler	Forest	0.26	0
1107	Male	Toddler	Forest	0	0
1108	Male	Toddler	Forest	0.06	0.05
1109	Male	Toddler	Forest	2.12	1.79
1110	Male	Toddler	Forest	0.21	0.03
1111	Male	Toddler	Forest	0.8	0.48
1112	Male	Old	Forest	8.41	0.16
1113	Male	Old	Forest	0.28	1.56
1114	Male	Old	Forest	0.8	0.42
1115	Male	Old	Forest	10.9	4.86
1116	Male	Old	Forest	0.1	1.22
1117	Male	Old	Forest	0.03	0.02
1118	Male	Old	Forest	0.3	0.36
1119	Male	Old	Forest	0	0
1120	Male	Old	Forest	0.67	0.06
1121	Male	Old	Forest	0.4	0.08
1122	Male	Old	Forest	1.21	1.39
1123	Male	Old	Forest	0.12	0.11
1124	Male	Old	Forest	1.43	0.02
1125	Male	Old	Forest	0.16	0.38
1126	Male	Old	Forest	0.56	0.45
1127	Male	Old	Forest	0.9	19.73
1128	Male	Old	Forest	3.56	0.88
1129	Male	Old	Forest	0.55	0.72
1130	Male	Old	Forest	0.08	0.02
1131	Male	Old	Forest	2.8	1.56
1132	Male	Old	Forest	0	0
1133	Male	Old	Forest	0.58	1.07
1134	Male	Old	Forest	0	0
1135	Male	Old	Forest	0.03	0.18
1136	Male	Old	Forest	0	0
1137	Male	Old	Forest	0	0
1138	Male	Old	Forest	2.65	0.38
1139	Male	Old	Forest	0	0

1140	Male	Old	Forest	0.22	2.31
1141	Male	Old	Forest	0.19	3.7
1142	Male	Old	Forest	7.75	0.45
1143	Male	Old	Forest	0.3	0.11
1144	Male	Old	Forest	0	0
1145	Male	Old	Forest	0.66	0.54
1146	Male	Old	Forest	0	0
1147	Male	Old	Forest	0.36	1.52
1148	Male	Old	Forest	0.35	30.01
1149	Male	Old	Forest	1.57	0.39
1150	Male	Old	Forest	4.01	3.83
1151	Male	Old	Forest	0	0
1152	Male	Old	Forest	0.25	0.54
1153	Male	Old	Forest	0.95	0.05
1154	Male	Old	Forest	0	0
1155	Male	Old	Forest	1.35	0.23
1156	Male	Old	Forest	1.59	0.17
1157	Male	Old	Forest	2.96	2.67
1158	Male	Old	Forest	0	0
1159	Male	Old	Forest	0.08	3.08
1160	Male	Old	Forest	0.09	2.26
1161	Male	Youth	Forest	22.85	5.71
1162	Male	Youth	Forest	0.64	0.04
1163	Male	Youth	Forest	1.24	0.06
1164	Male	Youth	Forest	0.69	1.68
1165	Male	Youth	Forest	0.41	8.38
1166	Male	Youth	Forest	1.79	0.38
1167	Male	Youth	Forest	0.09	0.02
1168	Male	Youth	Forest	3.71	0.21
1169	Male	Youth	Forest	0.87	0.55
1170	Male	Youth	Forest	0.65	1.91
1171	Male	Youth	Forest	4.87	5.8
1172	Male	Youth	Forest	0.15	0.43
1173	Male	Youth	Forest	1.93	2.64
1174	Male	Youth	Forest	3.65	0.18
1175	Male	Youth	Forest	0	0
1176	Male	Youth	Forest	0.22	0.04
1177	Male	Youth	Forest	0.19	1.76
1178	Male	Youth	Forest	0.25	0.14
1179	Male	Youth	Forest	0.36	0.04
1180	Male	Youth	Forest	0.68	29.98
1181	Male	Youth	Forest	0	0
1182	Male	Youth	Forest	1.73	1.91
1183	Male	Youth	Forest	10.91	3.9
1184	Male	Youth	Forest	0.13	8.22
1185	Male	Youth	Forest	0	0
1186	Male	Youth	Forest	0.37	3.08
1187	Male	Youth	Forest	0.17	0.02
1188	Male	Youth	Forest	0.09	0.03
1189	Male	Youth	Forest	27.95	0.15
1190	Male	Youth	Forest	0.45	1.96
1191	Male	Youth	Forest	0.78	0.76
1192	Male	Youth	Forest	0	0
1193	Male	Youth	Forest	0.96	0.25
1194	Male	Youth	Forest	0.24	0.02
1195	Male	Youth	Forest	8.84	1.59
1196	Male	Youth	Forest	31.26	2.22

1197	Male	Youth	Forest	0.64	0.35
1198	Male	Youth	Forest	1.07	0.11
1199	Male	Youth	Forest	1.36	1.51
1200	Male	Youth	Forest	1.87	16.2
1201	Male	Youth	Forest	0.12	0.74
1202	Male	Youth	Forest	0.56	0.91
1203	Male	Youth	Forest	0.13	8.66
1204	Male	Youth	Forest	1.05	0.81
1205	Male	Youth	Forest	7.11	3.36
1206	Male	Youth	Forest	0	0
1207	Male	Youth	Forest	0	0
1208	Male	Youth	Forest	0	0
1209	Male	Youth	Forest	1.92	2.03
1210	Male	Youth	Forest	1.99	0.61
1211	Male	Youth	Forest	0.1	3.29
1212	Male	Youth	Forest	0.65	0.03
1213	Male	Youth	Forest	0.29	0.24
1214	Male	Youth	Forest	0.3	2.44
1215	Male	Youth	Forest	2.02	2.23
1216	Male	Youth	Forest	0.19	0.19
1217	Male	Youth	Forest	3.93	0.25
1218	Male	Youth	Forest	8.04	0.71
1219	Male	Youth	Forest	1.81	0.03
1220	Male	Youth	Forest	1.36	0.05
1221	Male	Youth	Forest	12.96	1.06
1222	Male	Youth	Forest	0.69	1.13
1223	Male	Youth	Forest	7.69	0.5
1224	Male	Youth	Forest	0.49	2.8
1225	Male	Youth	Forest	0.22	0.67
1226	Male	Youth	Forest	0.65	4.75
1227	Male	Youth	Forest	1.22	0.39
1228	Male	Youth	Forest	0.75	1.02
1229	Male	Youth	Forest	0.17	0.67
1230	Male	Youth	Forest	2.96	4.84
1231	Male	Youth	Forest	1.82	0.33
1232	Male	Youth	Forest	22.03	10.63
1233	Male	Youth	Forest	8.38	11.27
1234	Male	Youth	Forest	8.21	0.82
1235	Male	Youth	Forest	0.87	0.22
1236	Male	Youth	Forest	16.26	2.84
1237	Male	Youth	Forest	2.67	2.95
1238	Male	Youth	Forest	0.2	0.23
1239	Male	Youth	Forest	0.81	0.65
1240	Male	Youth	Forest	6.66	1.53
1241	Male	Youth	Forest	1.53	1.8
1242	Male	Youth	Forest	1.64	0.92
1243	Male	Youth	Forest	0	0
1244	Male	Youth	Forest	1.21	6.4
1245	Male	Youth	Forest	4.25	9.03
1246	Male	Youth	Forest	0.09	0.01
1247	Male	Youth	Forest	11.95	0.92
1248	Male	Youth	Forest	0	0
1249	Male	Youth	Forest	0.94	0.59
1250	Male	Youth	Forest	2.24	7.1
1251	Male	Youth	Forest	0.85	0.52
1252	Male	Youth	Forest	0.17	0.79
1253	Male	Youth	Forest	2.16	1.72

1254	Male	Youth	Forest	0.12	0.01
1255	Male	Youth	Forest	0.74	1.22
1256	Male	Youth	Forest	3.41	1.36
1257	Male	Youth	Forest	7.21	2.76
1258	Male	Youth	Forest	0.18	62.03
1259	Male	Youth	Forest	8.97	4.31
1260	Male	Youth	Forest	8.38	4.68
1261	Male	Youth	Forest	7.83	27.38
1262	Male	Youth	Forest	0.52	0.82
1263	Male	Youth	Forest	0.05	0.16
1264	Male	Youth	Forest	6.3	0.09
1265	Male	Youth	Forest	0.47	0.09
1266	Male	Youth	Forest	4.39	21.28
1267	Male	Youth	Forest	1.75	39.39
1268	Male	Youth	Forest	0	0
1269	Male	Youth	Forest	5.38	0.15
1270	Male	Youth	Forest	0.74	7.82
1271	Female	Middle	Forest	0.63	1.79
1272	Female	Middle	Forest	27.02	0.67
1273	Female	Middle	Forest	0.14	3.29
1274	Female	Middle	Forest	2.98	0.85
1275	Female	Middle	Forest	0.12	6.56
1276	Female	Middle	Forest	0.83	0.36
1277	Female	Middle	Forest	0.87	1.88
1278	Female	Middle	Forest	0.59	0.9
1279	Female	Middle	Forest	0.01	0.49
1280	Female	Middle	Forest	0	0
1281	Female	Middle	Forest	0.34	0.12
1282	Female	Middle	Forest	0.45	3.04
1283	Female	Middle	Forest	0.36	3.35
1284	Female	Middle	Forest	0.37	0.11
1285	Female	Middle	Forest	0.18	0.21
1286	Female	Middle	Forest	7.33	0.5
1287	Female	Middle	Forest	0.12	0.15
1288	Female	Middle	Forest	24.18	0.8
1289	Female	Middle	Forest	0.49	0.89
1290	Female	Middle	Forest	1.62	15.38
1291	Female	Middle	Forest	3.72	0.38
1292	Female	Middle	Forest	0.04	2.04
1293	Female	Middle	Forest	0.31	23.57
1294	Female	Middle	Forest	0.04	1.87
1295	Female	Middle	Forest	0.09	3.93
1296	Female	Middle	Forest	2.62	2.27
1297	Female	Middle	Forest	9.58	10.14
1298	Female	Toddler	Forest	0.3	2.32
1299	Female	Toddler	Forest	0.12	2.92
1300	Female	Toddler	Forest	0.99	0.26
1301	Female	Toddler	Forest	0.08	0.08
1302	Female	Toddler	Forest	0.15	0.13
1303	Female	Toddler	Forest	0.11	2.08
1304	Female	Old	Forest	0	0
1305	Female	Old	Forest	0.16	0.53
1306	Female	Old	Forest	0.01	64.2
1307	Female	Old	Forest	5.43	0.84
1308	Female	Old	Forest	0.3	16.92
1309	Female	Old	Forest	0.12	8.02
1310	Female	Old	Forest	0.38	0.09

1311	Female	Old	Forest	1.31	2.12
1312	Female	Old	Forest	0	0
1313	Female	Old	Forest	0.42	0.08
1314	Female	Old	Forest	0.76	0.02
1315	Female	Old	Forest	0	0
1316	Female	Old	Forest	0.06	0.11
1317	Female	Old	Forest	2.24	1.62
1318	Female	Old	Forest	1.13	4.36
1319	Female	Old	Forest	0.07	3.56
1320	Female	Old	Forest	0.05	0.03
1321	Female	Old	Forest	0.18	0.83
1322	Female	Old	Forest	0.01	0.01
1323	Female	Old	Forest	4.75	0.03
1324	Female	Old	Forest	0.05	2.01
1325	Female	Old	Forest	0.09	4.4
1326	Female	Old	Forest	1.16	2.97
1327	Female	Old	Forest	1.2	4.7
1328	Female	Old	Forest	0.41	0.14
1329	Female	Old	Forest	0.88	10.63
1330	Female	Old	Forest	0.06	0.2
1331	Female	Old	Forest	0.1	0.04
1332	Female	Old	Forest	0.1	0.28
1333	Female	Old	Forest	0	0
1334	Female	Old	Forest	0.26	0.99
1335	Female	Old	Forest	1.71	1.72
1336	Female	Old	Forest	0.03	0.8
1337	Female	Old	Forest	1.1	0.16
1338	Female	Old	Forest	0.01	5.37
1339	Female	Old	Forest	0.16	4.61
1340	Female	Old	Forest	0.34	3.44
1341	Female	Youth	Forest	0.28	2.26
1342	Female	Youth	Forest	0.18	3.83
1343	Female	Youth	Forest	2.43	3.2
1344	Female	Youth	Forest	6.53	1.41
1345	Female	Youth	Forest	0.13	1.16
1346	Female	Youth	Forest	0.93	0.05
1347	Female	Youth	Forest	0.74	2.28
1348	Female	Youth	Forest	0.04	3.25
1349	Female	Youth	Forest	0.07	8.01
1350	Female	Youth	Forest	0.93	6
1351	Female	Youth	Forest	0.23	45.45
1352	Female	Youth	Forest	0.73	1
1353	Female	Youth	Forest	5.03	2.22
1354	Female	Youth	Forest	1.41	4.89
1355	Female	Youth	Forest	0.05	0.52
1356	Male	Middle	Forest	3.17	8.5
1357	Male	Middle	Forest	0.34	1.38
1358	Male	Middle	Forest	0.21	2.87
1359	Male	Middle	Forest	0.1	2.85
1360	Male	Middle	Forest	0.21	3.33
1361	Male	Middle	Forest	0.62	4.72
1362	Male	Middle	Forest	0.7	3.34
1363	Male	Middle	Forest	0.05	1.38
1364	Male	Middle	Forest	0.61	1.08
1365	Male	Middle	Forest	0.48	0.27
1366	Male	Middle	Forest	0.33	0.81
1367	Male	Middle	Forest	0.07	0.18

1368	Male	Middle	Forest	2.97	2.14
1369	Male	Middle	Forest	0.05	0.09
1370	Male	Middle	Forest	0.57	4.83
1371	Male	Middle	Forest	0	0
1372	Male	Middle	Forest	0.43	7.47
1373	Male	Middle	Forest	1.32	0.23
1374	Male	Middle	Forest	0.06	0.13
1375	Male	Middle	Forest	0.2	5.91
1376	Male	Middle	Forest	1.51	20.23
1377	Male	Middle	Forest	0.05	13.04
1378	Male	Middle	Forest	1.5	0.22
1379	Male	Toddler	Forest	1.43	0.86
1380	Male	Toddler	Forest	1.23	6
1381	Male	Old	Forest	0.68	0.37
1382	Male	Old	Forest	1.14	2.32
1383	Male	Old	Forest	0	0
1384	Male	Old	Forest	0	0
1385	Male	Old	Forest	0.22	3.59
1386	Male	Old	Forest	0.87	2.72
1387	Male	Old	Forest	0.03	0.02
1388	Male	Old	Forest	1.77	16.95
1389	Male	Old	Forest	0	0
1390	Male	Old	Forest	0.86	2.06
1391	Male	Old	Forest	0	0
1392	Male	Old	Forest	2.79	0.08
1393	Male	Old	Forest	0.56	7.9
1394	Male	Old	Forest	0.21	2.43
1395	Male	Old	Forest	0.28	0.02
1396	Male	Old	Forest	0.08	0.34
1397	Male	Old	Forest	0.74	0.14
1398	Male	Old	Forest	0	0
1399	Male	Old	Forest	0.12	3.6
1400	Male	Old	Forest	0.31	0.57
1401	Male	Old	Forest	0.05	2.44
1402	Male	Old	Forest	0.33	2.63
1403	Male	Old	Forest	0.28	5.03
1404	Male	Old	Forest	1.31	3.15
1405	Male	Old	Forest	0.38	0.62
1406	Male	Old	Forest	0	0
1407	Male	Old	Forest	0.05	0.16
1408	Male	Old	Forest	0.11	0.07
1409	Male	Old	Forest	0.14	2.2
1410	Male	Old	Forest	0.07	3.2
1411	Male	Old	Forest	0	0
1412	Male	Old	Forest	0.02	6.25
1413	Male	Youth	Forest	0.3	0.12
1414	Male	Youth	Forest	1	0.04
1415	Male	Youth	Forest	0.49	1.11
1416	Male	Youth	Forest	4.01	0.53
1417	Male	Youth	Forest	1.92	0.33
1418	Male	Youth	Forest	0.23	1.66
1419	Male	Youth	Forest	1.41	3.64
1420	Male	Youth	Forest	0.49	9.12
1421	Male	Youth	Forest	0.7	38.68
1422	Male	Youth	Forest	0	0
1423	Male	Youth	Forest	0.32	3.17
1424	Female	Middle	Forest	0	0

1425	Female	Middle	Forest	2.07	3.36
1426	Female	Middle	Forest	0	0
1427	Female	Middle	Forest	0	0
1428	Female	Middle	Forest	4.37	5.58
1429	Female	Middle	Forest	0.31	0.07
1430	Female	Toddler	Forest	4.24	4.09
1431	Female	Toddler	Forest	1.11	2.43
1432	Female	Toddler	Forest	0.98	2.33
1433	Female	Toddler	Forest	7.67	0.05
1434	Female	Old	Forest	0.54	0.17
1435	Female	Youth	Forest	14.4	0.56
1436	Female	Youth	Forest	0	0
1437	Female	Youth	Forest	11.42	0.35
1438	Female	Youth	Forest	3.83	0.4
1439	Male	Middle	Forest	7.06	3.01
1440	Male	Middle	Forest	0.45	7.84
1441	Male	Middle	Forest	0.71	0.07
1442	Male	Middle	Forest	3.06	0.28
1443	Male	Middle	Forest	0.54	0.38
1444	Male	Toddler	Forest	24.53	4.59
1445	Male	Toddler	Forest	2.11	0.12
1446	Male	Toddler	Forest	6.05	0.06
1447	Male	Toddler	Forest	10.73	0.15
1448	Male	Toddler	Forest	51.5	0.05
1449	Male	Old	Forest	41.61	0.91
1450	Male	Old	Forest	0.05	0.07
1451	Male	Old	Forest	4.46	0.45
1452	Male	Old	Forest	2.62	0.83
1453	Male	Old	Forest	1.08	10.22
1454	Male	Old	Forest	0.95	2.68
1455	Male	Youth	Forest	2.39	0.44
1456	Male	Youth	Forest	7.49	0.35
1457	Male	Youth	Forest	47.6	2.23
1458	Female	Middle	Forest	0.26	0.22
1459	Female	Middle	Forest	3.82	1.98
1460	Female	Middle	Forest	0.16	0.6
1461	Female	Middle	Forest	1.7	1.42
1462	Female	Middle	Forest	1.71	0
1463	Female	Middle	Forest	1.67	1.34
1464	Female	Middle	Forest	0	0
1465	Female	Middle	Forest	0.23	0.57
1466	Female	Middle	Forest	0	0
1467	Female	Middle	Forest	0.77	0.02
1468	Female	Middle	Forest	1.41	0.97
1469	Female	Middle	Forest	6.4	0.26
1470	Female	Middle	Forest	10.93	0.94
1471	Female	Middle	Forest	44.45	0.09
1472	Female	Toddler	Forest	0.14	0.08
1473	Female	Toddler	Forest	1.9	0.01
1474	Female	Toddler	Forest	13.25	5.29
1475	Female	Toddler	Forest	0.91	0.12
1476	Female	Toddler	Forest	0.48	0
1477	Female	Toddler	Forest	0.22	0.06
1478	Female	Old	Forest	1.24	0.29
1479	Female	Old	Forest	1.27	0.02
1480	Female	Old	Forest	1.98	0.34
1481	Female	Old	Forest	5.92	0.81

1482	Female	Old	Forest	1.68	2.51
1483	Female	Old	Forest	7.44	7.73
1484	Female	Old	Forest	4.34	2.32
1485	Female	Youth	Forest	2.55	0.34
1486	Female	Youth	Forest	10.44	0.23
1487	Female	Youth	Forest	37.67	0.21
1488	Female	Youth	Forest	0.15	0.08
1489	Female	Youth	Forest	4.51	0.18
1490	Female	Youth	Forest	4.43	0.38
1491	Female	Youth	Forest	2.27	0.2
1492	Female	Youth	Forest	27.14	3.08
1493	Female	Youth	Forest	9.6	0.34
1494	Female	Youth	Forest	1.15	0.68
1495	Female	Youth	Forest	3.02	0.2
1496	Female	Youth	Forest	8.29	0.43
1497	Female	Youth	Forest	2.25	11.8
1498	Female	Youth	Forest	4.35	1.3
1499	Male	Middle	Forest	4.96	2.06
1500	Male	Middle	Forest	1.17	0.36
1501	Male	Middle	Forest	1.13	0.12
1502	Male	Middle	Forest	5.18	0.11
1503	Male	Middle	Forest	1.89	0.01
1504	Male	Middle	Forest	0.54	0.24
1505	Male	Middle	Forest	6.58	1.13
1506	Male	Middle	Forest	0.39	2.51
1507	Male	Middle	Forest	15.51	1.02
1508	Male	Middle	Forest	0.04	0.05
1509	Male	Middle	Forest	19.9	0.06
1510	Male	Middle	Forest	5.6	11.69
1511	Male	Middle	Forest	4.3	2.55
1512	Male	Middle	Forest	4.59	0.44
1513	Male	Middle	Forest	23.93	4.34
1514	Male	Middle	Forest	0	0
1515	Male	Middle	Forest	2.55	0.07
1516	Male	Toddler	Forest	1.91	0.34
1517	Male	Toddler	Forest	0.86	0.07
1518	Male	Toddler	Forest	2.68	0.75
1519	Male	Toddler	Forest	1.19	0.02
1520	Male	Toddler	Forest	4.02	0.15
1521	Male	Toddler	Forest	8.96	0.05
1522	Male	Toddler	Forest	0.87	4.48
1523	Male	Old	Forest	0.18	3.94
1524	Male	Old	Forest	0	0
1525	Male	Old	Forest	4.15	8.17
1526	Male	Old	Forest	37.95	0.25
1527	Male	Old	Forest	0	0
1528	Male	Old	Forest	0.52	3.18
1529	Male	Old	Forest	0	0
1530	Male	Old	Forest	1.24	0.17
1531	Male	Old	Forest	1.32	4.84
1532	Male	Old	Forest	2.27	1.38
1533	Male	Old	Forest	0.45	1.03
1534	Male	Old	Forest	2.93	0.07
1535	Male	Old	Forest	0.27	0.15
1536	Male	Youth	Forest	2.89	3.86
1537	Male	Youth	Forest	0	0
1538	Male	Youth	Forest	25.8	0.17

1539	Male	Youth	Forest	1.32	0.01
1540	Male	Youth	Forest	11.69	0.52
1541	Male	Youth	Forest	3.42	3.23
1542	Male	Youth	Forest	0	0
1543	Male	Youth	Forest	7.34	2.04
1544	Male	Youth	Forest	1.58	0.45
1545	Male	Youth	Forest	1.67	1.51
1546	Male	Youth	Forest	7.09	5.18
1547	Male	Youth	Forest	0.74	0.82
1548	Male	Youth	Forest	1.81	19.38
1549	Male	Youth	Forest	1.95	3.56
1550	Male	Youth	Forest	0	0
1551	Male	Youth	Forest	5.96	0.97
1552	Female	Middle	Urban	0	0.47
1553	Female	Middle	Urban	0.67	0.61
1554	Female	Middle	Urban	1.26	6.74
1555	Female	Middle	Urban	0	0
1556	Female	Middle	Urban	0.78	2.28
1557	Female	Middle	Urban	3.68	3.77
1558	Female	Middle	Urban	31.89	25.63
1559	Female	Middle	Urban	0.26	7.76
1560	Female	Middle	Urban	0.05	2.82
1561	Female	Middle	Urban	2.22	7.11
1562	Female	Middle	Urban	0.43	8.91
1563	Female	Middle	Urban	10.45	4.06
1564	Female	Middle	Urban	1.55	2.07
1565	Female	Middle	Urban	0.09	1.33
1566	Female	Middle	Urban	0	0
1567	Female	Middle	Urban	0	0
1568	Female	Middle	Urban	0.82	0.01
1569	Female	Middle	Urban	2.08	1.39
1570	Female	Middle	Urban	1.65	0.56
1571	Female	Middle	Urban	24.96	1.88
1572	Female	Old	Urban	1.31	1.61
1573	Female	Old	Urban	0	0
1574	Female	Old	Urban	0.86	6.83
1575	Female	Old	Urban	1.24	69.54
1576	Female	Youth	Urban	35.09	2.05
1577	Female	Youth	Urban	5.65	3.72
1578	Female	Youth	Urban	0	0
1579	Female	Youth	Urban	6.17	10
1580	Female	Youth	Urban	4.12	12.63
1581	Female	Youth	Urban	1.28	6.16
1582	Female	Youth	Urban	0	0.01
1583	Female	Youth	Urban	1.1	3.21
1584	Female	Youth	Urban	9.94	0.45
1585	Female	Youth	Urban	18.84	2.66
1586	Female	Youth	Urban	13.79	23.21
1587	Female	Youth	Urban	0	0
1588	Female	Youth	Urban	6.09	3.24
1589	Female	Youth	Urban	11.42	1.85
1590	Female	Youth	Urban	0.4	0.18
1591	Male	Middle	Urban	0.77	0.22
1592	Male	Middle	Urban	0.34	0.29
1593	Male	Middle	Urban	0	0
1594	Male	Middle	Urban	14.02	4.43
1595	Male	Middle	Urban	15.59	0.03

1596	Male	Middle	Urban	2.04	0.37
1597	Male	Middle	Urban	1.02	3.94
1598	Male	Middle	Urban	0	0
1599	Male	Middle	Urban	1.04	1.21
1600	Male	Middle	Urban	4.46	3.88
1601	Male	Middle	Urban	3.37	10.28
1602	Male	Middle	Urban	2.03	0.8
1603	Male	Middle	Urban	0	0
1604	Male	Middle	Urban	1.73	30
1605	Male	Middle	Urban	21.48	0.86
1606	Male	Middle	Urban	0	0
1607	Male	Middle	Urban	6.39	22.62
1608	Male	Middle	Urban	0.92	0.36
1609	Male	Middle	Urban	0.2	0.56
1610	Male	Middle	Urban	0.67	16.72
1611	Male	Middle	Urban	0	0
1612	Male	Middle	Urban	0	0
1613	Male	Middle	Urban	0.04	0.4
1614	Male	Old	Urban	0	0
1615	Male	Old	Urban	0	0
1616	Male	Old	Urban	4.36	5.62
1617	Male	Old	Urban	0	0
1618	Male	Old	Urban	0.27	2.4
1619	Male	Old	Urban	0.14	0.32
1620	Male	Old	Urban	59.41	2.52
1621	Male	Old	Urban	6.49	24.72
1622	Male	Old	Urban	0	0
1623	Male	Old	Urban	0	0
1624	Male	Old	Urban	0.56	1.09
1625	Male	Old	Urban	1.2	3.45
1626	Male	Old	Urban	0	0
1627	Male	Youth	Urban	0.77	1
1628	Male	Youth	Urban	1.09	1.29
1629	Male	Youth	Urban	0.78	0.12
1630	Male	Youth	Urban	0.74	0.63
1631	Male	Youth	Urban	5.45	0.5
1632	Male	Youth	Urban	0.62	2.73
1633	Male	Youth	Urban	2.41	4.13
1634	Male	Youth	Urban	4.24	0.09
1635	Male	Youth	Urban	0.49	7.64
1636	Male	Youth	Urban	0.75	27.53
1637	Male	Youth	Urban	1.38	0.23
1638	Male	Youth	Urban	0.6	6.07
1639	Male	Youth	Urban	0.85	0.76
1640	Male	Youth	Urban	2.66	3.85
1641	Male	Youth	Urban	0	0
1642	Male	Youth	Urban	1.11	1.3
1643	Male	Youth	Urban	0.4	1.23
1644	Male	Youth	Urban	0.86	3.97
1645	Male	Youth	Urban	0	0
1646	Male	Youth	Urban	0	0
1647	Male	Youth	Urban	0.15	1.61
1648	Male	Youth	Urban	0.25	0.32
1649	Female	Middle	Urban	1.36	0.69
1650	Female	Middle	Urban	0.27	0.18
1651	Female	Middle	Urban	3.99	0.21
1652	Female	Middle	Urban	1.46	0.25

1653	Female	Middle	Urban	0.3	1.59
1654	Female	Middle	Urban	0.23	3.31
1655	Female	Middle	Urban	0.28	0.86
1656	Female	Middle	Urban	1.53	18.49
1657	Female	Middle	Urban	0.16	8.69
1658	Female	Middle	Urban	0.48	78.69
1659	Female	Middle	Urban	0	0
1660	Female	Middle	Urban	0.29	1.04
1661	Female	Middle	Urban	0.33	46.08
1662	Female	Middle	Urban	0	0
1663	Female	Middle	Urban	0.03	0.38
1664	Female	Middle	Urban	0.56	0.1
1665	Female	Middle	Urban	3.29	8.07
1666	Female	Middle	Urban	0.34	0.48
1667	Female	Middle	Urban	9.5	3.48
1668	Female	Middle	Urban	0.12	1.03
1669	Female	Middle	Urban	0.37	10.19
1670	Female	Middle	Urban	0	0
1671	Female	Middle	Urban	0.29	1.39
1672	Female	Middle	Urban	0.3	1.17
1673	Female	Toddler	Urban	0	0
1674	Female	Old	Urban	0.13	0.06
1675	Female	Old	Urban	0	0
1676	Female	Old	Urban	4.49	1.31
1677	Female	Old	Urban	0.01	46.61
1678	Female	Old	Urban	0	0
1679	Female	Old	Urban	0	0
1680	Female	Old	Urban	0.64	10.43
1681	Female	Old	Urban	0	0
1682	Female	Old	Urban	3.62	3.58
1683	Female	Old	Urban	0.19	6.08
1684	Female	Old	Urban	0.1	0.94
1685	Female	Youth	Urban	3.16	80.23
1686	Female	Youth	Urban	1.84	38.76
1687	Female	Youth	Urban	1.21	7.44
1688	Female	Youth	Urban	0.24	9.27
1689	Female	Youth	Urban	1.23	0.14
1690	Female	Youth	Urban	15.41	4.32
1691	Female	Youth	Urban	4.25	0.42
1692	Female	Youth	Urban	19.86	0.18
1693	Female	Youth	Urban	2.25	2.68
1694	Female	Youth	Urban	7.6	16.54
1695	Female	Youth	Urban	4.39	0.13
1696	Female	Youth	Urban	1.97	0.09
1697	Female	Youth	Urban	0.27	24.44
1698	Female	Youth	Urban	3.95	3.66
1699	Female	Youth	Urban	0.67	1.87
1700	Female	Youth	Urban	0.36	0.99
1701	Male	Middle	Urban	1.3	24.61
1702	Male	Middle	Urban	19.12	1.78
1703	Male	Middle	Urban	0.54	3.36
1704	Male	Middle	Urban	1.62	7.41
1705	Male	Middle	Urban	9.54	0.35
1706	Male	Middle	Urban	1.07	3.47
1707	Male	Middle	Urban	7.22	0.9
1708	Male	Middle	Urban	2.39	3.94
1709	Male	Middle	Urban	0	0

1710	Male	Middle	Urban	0	0
1711	Male	Middle	Urban	0	0
1712	Male	Middle	Urban	0	0
1713	Male	Middle	Urban	0.18	2.52
1714	Male	Middle	Urban	14.94	30.02
1715	Male	Middle	Urban	0	0
1716	Male	Middle	Urban	0	0
1717	Male	Old	Urban	15.84	3.4
1718	Male	Old	Urban	0	0
1719	Male	Old	Urban	0	0
1720	Male	Old	Urban	0.39	2.3
1721	Male	Old	Urban	0.44	0.34
1722	Male	Old	Urban	4.25	1.79
1723	Male	Old	Urban	0	0
1724	Male	Old	Urban	3.16	14.2
1725	Male	Old	Urban	2.25	0.18
1726	Male	Old	Urban	0.96	0.15
1727	Male	Old	Urban	1.53	0.4
1728	Male	Old	Urban	3.59	1.66
1729	Male	Youth	Urban	4.61	0.25
1730	Male	Youth	Urban	17.05	0.41
1731	Male	Youth	Urban	1.9	0.89
1732	Male	Youth	Urban	3.09	0.41
1733	Male	Youth	Urban	1.47	0.76
1734	Male	Youth	Urban	0	0
1735	Male	Youth	Urban	4.85	1.55
1736	Male	Youth	Urban	2.83	4.05
1737	Male	Youth	Urban	40.27	0.31
1738	Male	Youth	Urban	0.22	3.18
1739	Male	Youth	Urban	7.17	6.24
1740	Male	Youth	Urban	5.13	2.61
1741	Male	Youth	Urban	10.04	2.91
1742	Male	Youth	Urban	0.14	0.5
1743	Male	Youth	Urban	0.75	2.5
1744	Male	Youth	Urban	0	0
1745	Female	Middle	Urban	0.87	5.75
1746	Female	Middle	Urban	0.96	4.55
1747	Female	Middle	Urban	0.02	3.58
1748	Female	Middle	Urban	0.61	4.95
1749	Female	Middle	Urban	0.35	0.08
1750	Female	Middle	Urban	2.09	34.52
1751	Female	Middle	Urban	0	0
1752	Female	Middle	Urban	3.55	16.24
1753	Female	Middle	Urban	0.25	0.07
1754	Female	Middle	Urban	0.97	16.79
1755	Female	Middle	Urban	0.36	54.8
1756	Female	Middle	Urban	0.46	42.86
1757	Female	Middle	Urban	0.47	0.13
1758	Female	Middle	Urban	2.79	1.24
1759	Female	Middle	Urban	0.89	41.39
1760	Female	Middle	Urban	2.56	34.09
1761	Female	Middle	Urban	0.31	15.56
1762	Female	Middle	Urban	0.12	1.99
1763	Female	Middle	Urban	3.33	2.24
1764	Female	Old	Urban	0.11	12.99
1765	Female	Old	Urban	0.05	5.58
1766	Female	Old	Urban	0.09	9.91

1767	Female	Old	Urban	0.03	0.31
1768	Female	Old	Urban	0.04	4.72
1769	Female	Old	Urban	0.27	3.93
1770	Female	Old	Urban	0.31	0.48
1771	Female	Old	Urban	0	0
1772	Female	Old	Urban	0.31	0.42
1773	Female	Old	Urban	0.07	5.67
1774	Female	Old	Urban	0.29	0.17
1775	Female	Old	Urban	1.17	2.38
1776	Female	Old	Urban	0.97	0.03
1777	Female	Old	Urban	2.01	5.26
1778	Female	Old	Urban	0	0
1779	Female	Youth	Urban	18.11	8.33
1780	Female	Youth	Urban	0.07	2.74
1781	Female	Youth	Urban	0.8	39.79
1782	Female	Youth	Urban	2.02	1.16
1783	Female	Youth	Urban	0.03	3.93
1784	Female	Youth	Urban	0.03	0.16
1785	Female	Youth	Urban	0.35	8.92
1786	Female	Youth	Urban	2.97	0.08
1787	Female	Youth	Urban	0.03	10.61
1788	Female	Youth	Urban	11.25	0.37
1789	Female	Youth	Urban	2.94	6.21
1790	Female	Youth	Urban	1.22	2.88
1791	Female	Youth	Urban	0.4	1.28
1792	Female	Youth	Urban	0.76	0.28
1793	Male	Middle	Urban	0	0
1794	Male	Middle	Urban	0.13	0.59
1795	Male	Middle	Urban	0.43	1.35
1796	Male	Middle	Urban	11.61	10.52
1797	Male	Middle	Urban	8.48	8.23
1798	Male	Middle	Urban	29.63	6.55
1799	Male	Middle	Urban	0.13	42.18
1800	Male	Middle	Urban	4.72	3.74
1801	Male	Middle	Urban	2.21	1.55
1802	Male	Middle	Urban	6.89	3.01
1803	Male	Middle	Urban	0.28	1.79
1804	Male	Middle	Urban	0.95	2.18
1805	Male	Middle	Urban	0.74	15.67
1806	Male	Middle	Urban	0.61	0.43
1807	Male	Middle	Urban	0.24	2.2
1808	Male	Middle	Urban	0	0
1809	Male	Toddler	Urban	0.34	4.23
1810	Male	Old	Urban	9.55	0.37
1811	Male	Old	Urban	0.74	38.37
1812	Male	Old	Urban	0.06	61.18
1813	Male	Old	Urban	0.29	18.99
1814	Male	Old	Urban	0.35	0.15
1815	Male	Old	Urban	0.86	52.4
1816	Male	Old	Urban	0.48	4.06
1817	Male	Old	Urban	0.74	1.51
1818	Male	Old	Urban	0	0
1819	Male	Old	Urban	0	0
1820	Male	Old	Urban	0.06	0.83
1821	Male	Old	Urban	0.28	0.63
1822	Male	Old	Urban	0.73	4.4
1823	Male	Old	Urban	0.5	52.74

1824	Male	Old	Urban	0.38	1.53
1825	Male	Youth	Urban	0.88	20.55
1826	Male	Youth	Urban	1.04	2.12
1827	Male	Youth	Urban	0.05	39.43
1828	Male	Youth	Urban	0.33	0.43
1829	Male	Youth	Urban	0.1	6.5
1830	Male	Youth	Urban	13.28	4.89
1831	Male	Youth	Urban	1.58	1.5
1832	Male	Youth	Urban	10.35	1.38
1833	Male	Youth	Urban	3.55	11.05
1834	Male	Youth	Urban	1.86	1.62
1835	Male	Youth	Urban	0	0
1836	Male	Youth	Urban	0.28	34.01
1837	Male	Youth	Urban	17.79	1.14
1838	Male	Youth	Urban	2.43	1.49
1839	Male	Youth	Urban	2.06	1.61
1840	Male	Youth	Urban	0.07	3.09
1841	Male	Youth	Urban	33.67	1.62
1842	Female	Middle	Urban	0.13	0.17
1843	Female	Middle	Urban	0.2	1.68
1844	Female	Middle	Urban	0.05	0.13
1845	Female	Middle	Urban	0.06	7.85
1846	Female	Middle	Urban	0	0
1847	Female	Middle	Urban	0.08	0.16
1848	Female	Middle	Urban	0.42	53.7
1849	Female	Middle	Urban	0.57	19.17
1850	Female	Middle	Urban	1.72	0.54
1851	Female	Middle	Urban	3.86	11.14
1852	Female	Middle	Urban	0.5	0.06
1853	Female	Middle	Urban	1.05	0.92
1854	Female	Middle	Urban	6.8	0.05
1855	Female	Middle	Urban	0.19	70.22
1856	Female	Middle	Urban	4.2	6.53
1857	Female	Middle	Urban	4.81	0.05
1858	Female	Middle	Urban	2.72	5.33
1859	Female	Middle	Urban	0.66	1.52
1860	Female	Middle	Urban	10.49	0.91
1861	Female	Middle	Urban	0.75	26.68
1862	Female	Middle	Urban	1.85	9.29
1863	Female	Middle	Urban	0.04	0.31
1864	Female	Middle	Urban	0.27	0.47
1865	Female	Toddler	Urban	1.52	3.82
1866	Female	Toddler	Urban	0	0
1867	Female	Toddler	Urban	0.07	0
1868	Female	Toddler	Urban	0.13	0.04
1869	Female	Toddler	Urban	0.16	0.09
1870	Female	Toddler	Urban	0.32	0.71
1871	Female	Toddler	Urban	0.13	0.06
1872	Female	Old	Urban	29.35	0.45
1873	Female	Old	Urban	0.51	0.65
1874	Female	Old	Urban	4.44	5.57
1875	Female	Old	Urban	0	0
1876	Female	Old	Urban	1.75	15.08
1877	Female	Old	Urban	3.76	0.55
1878	Female	Old	Urban	0.2	3.94
1879	Female	Youth	Urban	21.26	1.04
1880	Female	Youth	Urban	10.39	0.26

1881	Female	Youth	Urban	0.63	0.74
1882	Female	Youth	Urban	1.5	3.15
1883	Female	Youth	Urban	0	0
1884	Female	Youth	Urban	5.5	1.86
1885	Female	Youth	Urban	0.13	0.17
1886	Female	Youth	Urban	0.75	1.24
1887	Female	Youth	Urban	0.23	2.89
1888	Female	Youth	Urban	3.62	23.85
1889	Female	Youth	Urban	27.84	4.98
1890	Female	Youth	Urban	0.06	0.9
1891	Female	Youth	Urban	0.01	0
1892	Female	Youth	Urban	0.76	15.3
1893	Female	Youth	Urban	1.49	3.71
1894	Female	Youth	Urban	0.09	4.44
1895	Female	Youth	Urban	0	0
1896	Female	Youth	Urban	0.31	0.18
1897	Female	Youth	Urban	9.27	0.04
1898	Female	Youth	Urban	0.42	0.01
1899	Female	Youth	Urban	26.08	4.07
1900	Female	Youth	Urban	4.78	0.64
1901	Female	Youth	Urban	5.97	7
1902	Female	Youth	Urban	1.58	6.45
1903	Female	Youth	Urban	0.29	0.45
1904	Female	Youth	Urban	0.91	0.44
1905	Female	Youth	Urban	0.33	1.53
1906	Female	Youth	Urban	0.2	0.01
1907	Female	Youth	Urban	1.14	1.89
1908	Female	Youth	Urban	16.61	1.23
1909	Female	Youth	Urban	25.22	39.91
1910	Female	Youth	Urban	7.69	3.35
1911	Female	Youth	Urban	0.21	0.03
1912	Female	Youth	Urban	0.18	0.31
1913	Female	Youth	Urban	5.27	0.03
1914	Female	Youth	Urban	0.26	0.05
1915	Female	Youth	Urban	0.13	0.01
1916	Female	Youth	Urban	0.05	0.01
1917	Female	Youth	Urban	1.5	13.02
1918	Female	Youth	Urban	0.77	0.49
1919	Female	Youth	Urban	6.64	0.6
1920	Female	Youth	Urban	1.98	2.85
1921	Female	Youth	Urban	0.09	0.04
1922	Female	Youth	Urban	1.19	23.83
1923	Female	Youth	Urban	0	0
1924	Female	Youth	Urban	2.59	0.1
1925	Female	Youth	Urban	0.07	0.05
1926	Female	Youth	Urban	0.33	11.52
1927	Female	Youth	Urban	4.16	0.12
1928	Female	Youth	Urban	0.08	85.09
1929	Female	Youth	Urban	12.26	5.7
1930	Female	Youth	Urban	14.29	2.44
1931	Female	Youth	Urban	3.24	8.82
1932	Female	Youth	Urban	4.05	0.3
1933	Female	Youth	Urban	4.99	3.77
1934	Female	Youth	Urban	4.23	8.22
1935	Female	Youth	Urban	1.53	3.51
1936	Female	Youth	Urban	0	0
1937	Female	Youth	Urban	2.85	0.01

1938	Female	Youth	Urban	1.22	1.19
1939	Female	Youth	Urban	5.04	11.78
1940	Female	Youth	Urban	0.24	16.93
1941	Female	Youth	Urban	0.33	47.74
1942	Female	Youth	Urban	9.08	1.63
1943	Female	Youth	Urban	0.15	0.02
1944	Female	Youth	Urban	19.03	9.16
1945	Female	Youth	Urban	0	0
1946	Female	Youth	Urban	8.43	9.53
1947	Female	Youth	Urban	6.51	2.52
1948	Female	Youth	Urban	4.57	0.27
1949	Female	Youth	Urban	1.23	0.11
1950	Male	Middle	Urban	0.72	0.01
1951	Male	Middle	Urban	2	1.36
1952	Male	Middle	Urban	0	0
1953	Male	Middle	Urban	0	0
1954	Male	Middle	Urban	0.02	2.15
1955	Male	Middle	Urban	0	0
1956	Male	Middle	Urban	3.21	0.32
1957	Male	Middle	Urban	0.3	5.38
1958	Male	Middle	Urban	0.98	17.02
1959	Male	Middle	Urban	0.94	6.86
1960	Male	Middle	Urban	1.08	4.64
1961	Male	Middle	Urban	0.07	0.29
1962	Male	Middle	Urban	1.18	37.75
1963	Male	Middle	Urban	0.19	2.26
1964	Male	Middle	Urban	0.27	1.4
1965	Male	Middle	Urban	0.08	0.53
1966	Male	Middle	Urban	2.82	19.43
1967	Male	Middle	Urban	0.26	0.39
1968	Male	Middle	Urban	0.18	18.2
1969	Male	Middle	Urban	0.08	0.08
1970	Male	Middle	Urban	15.9	1.98
1971	Male	Middle	Urban	1.15	8.81
1972	Male	Middle	Urban	3.9	0.12
1973	Male	Middle	Urban	0.3	6.87
1974	Male	Middle	Urban	0.27	0.07
1975	Male	Middle	Urban	0	0
1976	Male	Middle	Urban	0	0.03
1977	Male	Middle	Urban	0	0
1978	Male	Toddler	Urban	0.93	3.13
1979	Male	Toddler	Urban	0	0
1980	Male	Toddler	Urban	0.97	2.4
1981	Male	Toddler	Urban	1.32	0.16
1982	Male	Toddler	Urban	0.28	0.64
1983	Male	Old	Urban	0.14	0.05
1984	Male	Old	Urban	1.04	1.66
1985	Male	Old	Urban	1.52	2.12
1986	Male	Old	Urban	0.76	0.44
1987	Male	Old	Urban	1.75	1.68
1988	Male	Old	Urban	2.61	0.52
1989	Male	Old	Urban	0.25	0.01
1990	Male	Old	Urban	0.19	0.2
1991	Male	Old	Urban	0	0
1992	Male	Old	Urban	0	0
1993	Male	Old	Urban	1.9	0.47
1994	Male	Old	Urban	0	0

1995	Male	Old	Urban	0	0
1996	Male	Old	Urban	0.25	0.5
1997	Male	Old	Urban	0.15	0.25
1998	Male	Old	Urban	0.01	0.13
1999	Male	Old	Urban	11.83	0.26
2000	Male	Old	Urban	0.02	61.67
2001	Male	Old	Urban	0.3	1.55
2002	Male	Old	Urban	0	0
2003	Male	Old	Urban	2.12	1.92
2004	Male	Old	Urban	0.03	0.02
2005	Male	Old	Urban	0.04	0.22
2006	Male	Old	Urban	0.96	1.27
2007	Male	Youth	Urban	0.12	0.01
2008	Male	Youth	Urban	0.8	1.05
2009	Male	Youth	Urban	0	0
2010	Male	Youth	Urban	16.39	0.08
2011	Male	Youth	Urban	0.62	2.13
2012	Male	Youth	Urban	1.43	0.82
2013	Male	Youth	Urban	8.99	4.85
2014	Male	Youth	Urban	0.02	0
2015	Male	Youth	Urban	0.11	1.2
2016	Male	Youth	Urban	3.12	2.32
2017	Male	Youth	Urban	0.21	29.78
2018	Male	Youth	Urban	0	0
2019	Male	Youth	Urban	6.78	0.71
2020	Male	Youth	Urban	17.32	5.01
2021	Male	Youth	Urban	0	0
2022	Male	Youth	Urban	1.49	2.29
2023	Male	Youth	Urban	0.24	36.07
2024	Male	Youth	Urban	0.04	0.04
2025	Male	Youth	Urban	0	0
2026	Male	Youth	Urban	0	0
2027	Male	Youth	Urban	9.42	33.63
2028	Male	Youth	Urban	0.4	0.2
2029	Male	Youth	Urban	0.53	4.39
2030	Male	Youth	Urban	0.33	0.04
2031	Male	Youth	Urban	0.55	0.38
2032	Male	Youth	Urban	3.7	2.26
2033	Male	Youth	Urban	2.2	0.52
2034	Male	Youth	Urban	1.27	3.07
2035	Male	Youth	Urban	17.22	1.22
2036	Male	Youth	Urban	1.68	25.64
2037	Male	Youth	Urban	28.52	0.37
2038	Male	Youth	Urban	0.36	0.21
2039	Male	Youth	Urban	0	0
2040	Male	Youth	Urban	10.36	2.96
2041	Male	Youth	Urban	1.05	22.47
2042	Male	Youth	Urban	1.88	3.69
2043	Male	Youth	Urban	3.69	6.75
2044	Male	Youth	Urban	2.22	0.63
2045	Male	Youth	Urban	0.16	0.22
2046	Male	Youth	Urban	1.31	44.36
2047	Male	Youth	Urban	0.05	12.05
2048	Male	Youth	Urban	0.02	78.77
2049	Male	Youth	Urban	0	0
2050	Male	Youth	Urban	0.18	0.37
2051	Male	Youth	Urban	4.75	14.05

2052	Male	Youth	Urban	0.39	0.11
2053	Male	Youth	Urban	0.04	0.04
2054	Male	Youth	Urban	0.71	1.55
2055	Male	Youth	Urban	0.05	84.57
2056	Male	Youth	Urban	3.35	0.24
2057	Male	Youth	Urban	6.72	0.21
2058	Male	Youth	Urban	1.39	1.44
2059	Male	Youth	Urban	37.05	13.76
2060	Male	Youth	Urban	1.19	0.09
2061	Male	Youth	Urban	0.33	0.19
2062	Male	Youth	Urban	0.02	0
2063	Male	Youth	Urban	12.2	0.3
2064	Male	Youth	Urban	0.11	0.03
2065	Male	Youth	Urban	0	0
2066	Male	Youth	Urban	6.23	35.04
2067	Male	Youth	Urban	5.51	1.49
2068	Male	Youth	Urban	0	0
2069	Male	Youth	Urban	24.27	5.09
2070	Male	Youth	Urban	9.93	1.7
2071	Male	Youth	Urban	0.05	0.05
2072	Male	Youth	Urban	0	0
2073	Male	Youth	Urban	1.24	0.26
2074	Male	Youth	Urban	1.13	8.66
2075	Male	Youth	Urban	0.1	18.34
2076	Female	Middle	Urban	0	0
2077	Female	Middle	Urban	0	0
2078	Female	Middle	Urban	0.13	4.21
2079	Female	Middle	Urban	6.07	0.1
2080	Female	Middle	Urban	0.13	0.82
2081	Female	Middle	Urban	0	0
2082	Female	Middle	Urban	0.27	0.06
2083	Female	Middle	Urban	0.22	0.02
2084	Female	Middle	Urban	0.84	1.71
2085	Female	Middle	Urban	0.67	0.28
2086	Female	Middle	Urban	0.13	1.77
2087	Female	Middle	Urban	2.87	4.69
2088	Female	Middle	Urban	0.32	1.82
2089	Female	Middle	Urban	0.29	19.58
2090	Female	Middle	Urban	0.85	1.43
2091	Female	Middle	Urban	0.52	0.62
2092	Female	Middle	Urban	0.31	0.4
2093	Female	Middle	Urban	1.9	0.99
2094	Female	Middle	Urban	0.22	0.59
2095	Female	Middle	Urban	0.74	2.65
2096	Female	Middle	Urban	10.34	25.24
2097	Female	Middle	Urban	0.22	23.15
2098	Female	Middle	Urban	6.86	4.23
2099	Female	Middle	Urban	1.95	20.97
2100	Female	Middle	Urban	1.04	1.82
2101	Female	Middle	Urban	1.53	1.96
2102	Female	Middle	Urban	15.3	20.44
2103	Female	Middle	Urban	16.19	41.6
2104	Female	Middle	Urban	0.26	7.15
2105	Female	Middle	Urban	1.46	0.04
2106	Female	Middle	Urban	0.89	2.4
2107	Female	Middle	Urban	53.26	0.42
2108	Female	Middle	Urban	0.19	5.13

2109	Female	Middle	Urban	0.11	21.49
2110	Female	Middle	Urban	5.51	32.2
2111	Female	Middle	Urban	9.99	13.87
2112	Female	Middle	Urban	0.07	0.6
2113	Female	Middle	Urban	0.08	4.02
2114	Female	Middle	Urban	0.85	0.14
2115	Female	Middle	Urban	0.23	8.78
2116	Female	Middle	Urban	0	0
2117	Female	Middle	Urban	1.32	3.78
2118	Female	Middle	Urban	0.75	0.38
2119	Female	Middle	Urban	11.96	0.31
2120	Female	Middle	Urban	0.19	4.23
2121	Female	Middle	Urban	0.05	0.14
2122	Female	Middle	Urban	17.06	0.92
2123	Female	Middle	Urban	0	0
2124	Female	Middle	Urban	0.71	10.38
2125	Female	Middle	Urban	0.56	0.24
2126	Female	Middle	Urban	0.27	1.86
2127	Female	Middle	Urban	0.05	7.67
2128	Female	Middle	Urban	0	0
2129	Female	Middle	Urban	42.82	0.18
2130	Female	Middle	Urban	27.99	0.82
2131	Female	Middle	Urban	0	0
2132	Female	Middle	Urban	2.63	0.8
2133	Female	Middle	Urban	0.16	0.35
2134	Female	Middle	Urban	2.28	2.73
2135	Female	Middle	Urban	0.13	0.19
2136	Female	Middle	Urban	0.42	3.3
2137	Female	Middle	Urban	3.49	0.12
2138	Female	Middle	Urban	0.73	9.14
2139	Female	Middle	Urban	0.34	7.64
2140	Female	Middle	Urban	0.11	34.94
2141	Female	Middle	Urban	3.87	0.4
2142	Female	Middle	Urban	4.49	8.44
2143	Female	Middle	Urban	4.73	1.17
2144	Female	Middle	Urban	3.24	9.82
2145	Female	Middle	Urban	1.62	30.01
2146	Female	Middle	Urban	0.33	11.66
2147	Female	Middle	Urban	1.6	31.72
2148	Female	Middle	Urban	8.49	1
2149	Female	Middle	Urban	0.16	1.11
2150	Female	Middle	Urban	0.19	3.99
2151	Female	Middle	Urban	0.38	0.74
2152	Female	Middle	Urban	1.66	72.33
2153	Female	Middle	Urban	0	2.93
2154	Female	Middle	Urban	1.3	16.48
2155	Female	Middle	Urban	9.3	1.65
2156	Female	Middle	Urban	0	0
2157	Female	Middle	Urban	0.38	0.68
2158	Female	Middle	Urban	0.84	1.44
2159	Female	Middle	Urban	0.09	14.65
2160	Female	Middle	Urban	2.38	7.57
2161	Female	Middle	Urban	33.8	0.8
2162	Female	Middle	Urban	0.04	35.02
2163	Female	Middle	Urban	1.45	9.33
2164	Female	Middle	Urban	2.79	0.26
2165	Female	Middle	Urban	3.64	0.63

2166	Female	Middle	Urban	0	0
2167	Female	Middle	Urban	11.12	19.05
2168	Female	Middle	Urban	1.07	10.4
2169	Female	Middle	Urban	0.56	3.8
2170	Female	Middle	Urban	0.6	0.25
2171	Female	Middle	Urban	0.34	2.73
2172	Female	Middle	Urban	0	0
2173	Female	Toddler	Urban	0.19	0.09
2174	Female	Toddler	Urban	0.09	0.56
2175	Female	Toddler	Urban	3.71	2.07
2176	Female	Toddler	Urban	0.1	0.05
2177	Female	Toddler	Urban	0.08	0.05
2178	Female	Toddler	Urban	0.32	0.81
2179	Female	Toddler	Urban	0.22	13.3
2180	Female	Old	Urban	0.71	0.76
2181	Female	Old	Urban	9.93	0.29
2182	Female	Old	Urban	0.32	0.45
2183	Female	Old	Urban	0.37	0.5
2184	Female	Old	Urban	0.25	0.3
2185	Female	Old	Urban	2.91	1.72
2186	Female	Old	Urban	0.35	3.22
2187	Female	Old	Urban	0	0
2188	Female	Old	Urban	0	0
2189	Female	Old	Urban	1.14	0.46
2190	Female	Old	Urban	0.56	5.41
2191	Female	Old	Urban	0	0
2192	Female	Old	Urban	0.89	0.04
2193	Female	Old	Urban	3.21	15.01
2194	Female	Old	Urban	0.57	0.12
2195	Female	Old	Urban	0.97	5.77
2196	Female	Old	Urban	2.03	5.22
2197	Female	Old	Urban	2.38	32.02
2198	Female	Old	Urban	9.97	0.58
2199	Female	Old	Urban	0.85	0.21
2200	Female	Old	Urban	3.76	0.96
2201	Female	Old	Urban	0.49	0.42
2202	Female	Old	Urban	0.11	1.67
2203	Female	Old	Urban	1.61	0.25
2204	Female	Old	Urban	16.01	7.47
2205	Female	Old	Urban	0.36	6.65
2206	Female	Youth	Urban	33.02	1.37
2207	Female	Youth	Urban	0.4	22.03
2208	Female	Youth	Urban	0.1	0.27
2209	Female	Youth	Urban	0.48	0.11
2210	Female	Youth	Urban	0.19	6.6
2211	Female	Youth	Urban	0.06	0.03
2212	Female	Youth	Urban	0	0
2213	Female	Youth	Urban	0.88	1.03
2214	Female	Youth	Urban	0.21	11.3
2215	Female	Youth	Urban	0.15	34.53
2216	Female	Youth	Urban	8.18	9.85
2217	Female	Youth	Urban	0.03	2.86
2218	Female	Youth	Urban	0.56	3.18
2219	Female	Youth	Urban	0.05	6.79
2220	Female	Youth	Urban	0	0
2221	Female	Youth	Urban	0.08	4.86
2222	Female	Youth	Urban	0.06	5.58

2223	Female	Youth	Urban	14.4	0.58
2224	Female	Youth	Urban	0	0
2225	Female	Youth	Urban	5.3	0.38
2226	Female	Youth	Urban	1.46	5.19
2227	Female	Youth	Urban	4.8	1.48
2228	Female	Youth	Urban	0.82	1.79
2229	Female	Youth	Urban	5.98	1.36
2230	Female	Youth	Urban	11.65	16.72
2231	Female	Youth	Urban	0.67	3.83
2232	Female	Youth	Urban	20.77	4.73
2233	Female	Youth	Urban	0.13	0.09
2234	Female	Youth	Urban	3.91	0.07
2235	Female	Youth	Urban	3.38	1.55
2236	Female	Youth	Urban	0.6	6.93
2237	Female	Youth	Urban	0.31	1.1
2238	Female	Youth	Urban	1.27	1.08
2239	Female	Youth	Urban	17.06	14.36
2240	Female	Youth	Urban	4.8	0.81
2241	Female	Youth	Urban	15.57	2.42
2242	Female	Youth	Urban	8.76	1.52
2243	Female	Youth	Urban	29.91	19.9
2244	Female	Youth	Urban	0.13	0.42
2245	Female	Youth	Urban	0	0
2246	Female	Youth	Urban	58.45	1.29
2247	Female	Youth	Urban	21.77	0.72
2248	Female	Youth	Urban	0.91	22.1
2249	Female	Youth	Urban	2.56	8.94
2250	Female	Youth	Urban	33.23	25.38
2251	Female	Youth	Urban	1.25	0.08
2252	Female	Youth	Urban	2.32	0.32
2253	Female	Youth	Urban	0	0
2254	Female	Youth	Urban	2.93	1.71
2255	Female	Youth	Urban	6.02	3.81
2256	Female	Youth	Urban	58.44	0.34
2257	Female	Youth	Urban	18.11	8.33
2258	Female	Youth	Urban	0.5	22.52
2259	Female	Youth	Urban	17.34	0.5
2260	Female	Youth	Urban	0.47	2.3
2261	Female	Youth	Urban	0.66	0.07
2262	Female	Youth	Urban	2.63	2.17
2263	Female	Youth	Urban	0.35	0.96
2264	Female	Youth	Urban	0.12	21.34
2265	Female	Youth	Urban	0.24	0.02
2266	Female	Youth	Urban	0.39	0.28
2267	Female	Youth	Urban	1.83	4.36
2268	Female	Youth	Urban	1.87	0.67
2269	Female	Youth	Urban	0.95	6.28
2270	Female	Youth	Urban	1	1.66
2271	Female	Youth	Urban	3.71	17.94
2272	Female	Youth	Urban	1.02	0.08
2273	Female	Youth	Urban	2.35	0.31
2274	Female	Youth	Urban	1.97	1.88
2275	Female	Youth	Urban	0.96	0.62
2276	Female	Youth	Urban	33.59	4.08
2277	Female	Youth	Urban	0	0
2278	Female	Youth	Urban	27.03	1.55
2279	Female	Youth	Urban	0.05	0.47

2280	Female	Youth	Urban	6.94	1.92
2281	Female	Youth	Urban	8.98	2.56
2282	Female	Youth	Urban	0.61	6.56
2283	Female	Youth	Urban	3.06	3.19
2284	Female	Youth	Urban	0.52	7.36
2285	Female	Youth	Urban	0.23	5.36
2286	Female	Youth	Urban	0.35	0.92
2287	Female	Youth	Urban	0	0
2288	Female	Youth	Urban	12.17	0.57
2289	Female	Youth	Urban	0.97	41.39
2290	Female	Youth	Urban	0.23	0.42
2291	Female	Youth	Urban	0.86	1.36
2292	Female	Youth	Urban	5.23	3.03
2293	Female	Youth	Urban	1.62	1.27
2294	Female	Youth	Urban	1.54	10.76
2295	Female	Youth	Urban	4.91	0.1
2296	Female	Youth	Urban	10.9	1.37
2297	Female	Youth	Urban	2.68	5.51
2298	Female	Youth	Urban	0.06	40.35
2299	Female	Youth	Urban	0	0
2300	Female	Youth	Urban	0.07	0.43
2301	Female	Youth	Urban	2.36	0.35
2302	Female	Youth	Urban	0.51	1.36
2303	Female	Youth	Urban	0.66	12.34
2304	Female	Youth	Urban	0.29	0.76
2305	Female	Youth	Urban	2.4	0.95
2306	Female	Youth	Urban	20.41	0.84
2307	Female	Youth	Urban	0.14	5.07
2308	Female	Youth	Urban	1.83	1.24
2309	Female	Youth	Urban	13.62	1.22
2310	Female	Youth	Urban	2.14	2.29
2311	Female	Youth	Urban	1.87	17.48
2312	Female	Youth	Urban	6.3	0.14
2313	Female	Youth	Urban	0.13	6.41
2314	Female	Youth	Urban	0.34	0.68
2315	Male	Middle	Urban	2.7	0.2
2316	Male	Middle	Urban	4.9	0.59
2317	Male	Middle	Urban	1.12	7.92
2318	Male	Middle	Urban	11.71	0.89
2319	Male	Middle	Urban	12.45	0.22
2320	Male	Middle	Urban	0.57	0.12
2321	Male	Middle	Urban	0.25	0.12
2322	Male	Middle	Urban	0.16	3.66
2323	Male	Middle	Urban	1.05	0.56
2324	Male	Middle	Urban	5.04	5.77
2325	Male	Middle	Urban	11.72	1.87
2326	Male	Middle	Urban	1.62	7.41
2327	Male	Middle	Urban	0.81	4.46
2328	Male	Middle	Urban	9.19	10.41
2329	Male	Middle	Urban	0.35	0.89
2330	Male	Middle	Urban	0	0
2331	Male	Middle	Urban	0	0
2332	Male	Middle	Urban	0.23	3.42
2333	Male	Middle	Urban	4.34	0.56
2334	Male	Middle	Urban	1.36	0.67
2335	Male	Middle	Urban	2.88	2.89
2336	Male	Middle	Urban	3.68	1.66

2337	Male	Middle	Urban	0.95	0.91
2338	Male	Middle	Urban	6.96	3.84
2339	Male	Middle	Urban	1.75	0.8
2340	Male	Middle	Urban	2.42	1.38
2341	Male	Middle	Urban	2.48	0.11
2342	Male	Middle	Urban	3.81	2.62
2343	Male	Middle	Urban	0	0
2344	Male	Middle	Urban	0	0
2345	Male	Middle	Urban	1.18	6.52
2346	Male	Middle	Urban	0	0
2347	Male	Middle	Urban	0	0
2348	Male	Middle	Urban	0.94	0.16
2349	Male	Middle	Urban	0.55	0.04
2350	Male	Middle	Urban	4.67	0.56
2351	Male	Middle	Urban	0.34	0.12
2352	Male	Middle	Urban	0.49	0.2
2353	Male	Middle	Urban	0	0
2354	Male	Middle	Urban	4.46	8.11
2355	Male	Middle	Urban	5.12	2.23
2356	Male	Middle	Urban	0.14	0.58
2357	Male	Middle	Urban	0.53	1.17
2358	Male	Middle	Urban	0	0
2359	Male	Middle	Urban	53.92	1.01
2360	Male	Middle	Urban	1.64	0.07
2361	Male	Middle	Urban	0.64	0.27
2362	Male	Middle	Urban	24.71	0.33
2363	Male	Middle	Urban	0.36	0.1
2364	Male	Middle	Urban	1.9	12.22
2365	Male	Middle	Urban	0.29	0.76
2366	Male	Middle	Urban	2.39	35.12
2367	Male	Middle	Urban	1.17	3.07
2368	Male	Middle	Urban	0	0
2369	Male	Middle	Urban	2.69	4.24
2370	Male	Middle	Urban	0.04	9.09
2371	Male	Middle	Urban	2.11	0.07
2372	Male	Middle	Urban	8.51	2.48
2373	Male	Middle	Urban	15.77	4.77
2374	Male	Middle	Urban	0.11	0.28
2375	Male	Middle	Urban	0	0
2376	Male	Middle	Urban	0.08	1.59
2377	Male	Middle	Urban	2.33	6.26
2378	Male	Middle	Urban	25.09	1.24
2379	Male	Middle	Urban	0.27	1.28
2380	Male	Middle	Urban	0.4	13.7
2381	Male	Middle	Urban	1.12	1.46
2382	Male	Middle	Urban	0	0
2383	Male	Middle	Urban	0.4	0.83
2384	Male	Middle	Urban	45.11	0.35
2385	Male	Middle	Urban	16.42	2.15
2386	Male	Middle	Urban	6.99	0.67
2387	Male	Middle	Urban	1.43	0.11
2388	Male	Middle	Urban	0.03	3.64
2389	Male	Toddler	Urban	0	0
2390	Male	Toddler	Urban	0	0
2391	Male	Toddler	Urban	0	0
2392	Male	Toddler	Urban	1.21	0.52
2393	Male	Toddler	Urban	1.6	4.55

2394	Male	Toddler	Urban	9.06	0.2
2395	Male	Toddler	Urban	0.06	0.01
2396	Male	Toddler	Urban	0	0
2397	Male	Toddler	Urban	1.73	10.38
2398	Male	Toddler	Urban	0.17	0.48
2399	Male	Toddler	Urban	2.3	33.76
2400	Male	Toddler	Urban	7	5.46
2401	Male	Toddler	Urban	15.27	2.01
2402	Male	Toddler	Urban	0	0
2403	Male	Toddler	Urban	0.27	8.9
2404	Male	Toddler	Urban	0.39	1.08
2405	Male	Toddler	Urban	0	0
2406	Male	Toddler	Urban	3.42	0.1
2407	Male	Old	Urban	3.53	10.82
2408	Male	Old	Urban	0.29	8.99
2409	Male	Old	Urban	6.22	7.84
2410	Male	Old	Urban	8.68	6.4
2411	Male	Old	Urban	2.09	2.06
2412	Male	Old	Urban	4.14	4.54
2413	Male	Old	Urban	0.1	0.1
2414	Male	Old	Urban	3.89	0.89
2415	Male	Old	Urban	0.07	17.42
2416	Male	Old	Urban	0	0
2417	Male	Old	Urban	1.18	3.13
2418	Male	Old	Urban	6.01	1.66
2419	Male	Old	Urban	0	0
2420	Male	Old	Urban	0	0
2421	Male	Old	Urban	0.19	0.95
2422	Male	Old	Urban	0.29	0.42
2423	Male	Old	Urban	0	0
2424	Male	Old	Urban	0.25	0.62
2425	Male	Old	Urban	0	0
2426	Male	Old	Urban	0.63	1.14
2427	Male	Old	Urban	0	0
2428	Male	Old	Urban	0	0
2429	Male	Old	Urban	0.05	0.56
2430	Male	Old	Urban	0	0
2431	Male	Old	Urban	6.96	10.27
2432	Male	Old	Urban	0	0
2433	Male	Old	Urban	0	0
2434	Male	Old	Urban	0	0
2435	Male	Old	Urban	1.13	0.59
2436	Male	Old	Urban	3.09	1.27
2437	Male	Old	Urban	0	0
2438	Male	Old	Urban	0.84	2.19
2439	Male	Old	Urban	3.83	9.52
2440	Male	Old	Urban	5.55	4.37
2441	Male	Old	Urban	0	0
2442	Male	Old	Urban	0	0
2443	Male	Old	Urban	0	0
2444	Male	Old	Urban	0	0
2445	Male	Old	Urban	1.99	16.67
2446	Male	Old	Urban	1.54	9.94
2447	Male	Old	Urban	13.45	6.89
2448	Male	Old	Urban	0	0
2449	Male	Old	Urban	0.56	0.5
2450	Male	Old	Urban	0.05	0.98

2451	Male	Old	Urban	0.5	0.79
2452	Male	Old	Urban	3.7	0.87
2453	Male	Old	Urban	0	0
2454	Male	Old	Urban	0	0
2455	Male	Old	Urban	0	0
2456	Male	Old	Urban	2.29	0.44
2457	Male	Old	Urban	0	0
2458	Male	Old	Urban	0	0
2459	Male	Old	Urban	1.42	1.21
2460	Male	Old	Urban	0.55	1.85
2461	Male	Old	Urban	0.85	7.17
2462	Male	Old	Urban	0.51	0.64
2463	Male	Old	Urban	9.39	0.85
2464	Male	Old	Urban	1.5	3.89
2465	Male	Old	Urban	6.43	1.18
2466	Male	Youth	Urban	20.79	15.05
2467	Male	Youth	Urban	1.08	1.65
2468	Male	Youth	Urban	23.69	4.83
2469	Male	Youth	Urban	0.11	0.25
2470	Male	Youth	Urban	5.57	1.01
2471	Male	Youth	Urban	6.07	1.82
2472	Male	Youth	Urban	0.8	12.01
2473	Male	Youth	Urban	5.22	27.81
2474	Male	Youth	Urban	0	0
2475	Male	Youth	Urban	1.25	0.54
2476	Male	Youth	Urban	0.64	53.39
2477	Male	Youth	Urban	0	0
2478	Male	Youth	Urban	0	0
2479	Male	Youth	Urban	0.64	0.51
2480	Male	Youth	Urban	24.29	2.03
2481	Male	Youth	Urban	0	0
2482	Male	Youth	Urban	0.24	0.84
2483	Male	Youth	Urban	0.83	1.48
2484	Male	Youth	Urban	6.1	9.95
2485	Male	Youth	Urban	0.58	0.28
2486	Male	Youth	Urban	0	0
2487	Male	Youth	Urban	0	0
2488	Male	Youth	Urban	1.56	0.21
2489	Male	Youth	Urban	0	0
2490	Male	Youth	Urban	1.88	0.01
2491	Male	Youth	Urban	0	0
2492	Male	Youth	Urban	4.93	0.26
2493	Male	Youth	Urban	0	0
2494	Male	Youth	Urban	22.74	3.39
2495	Male	Youth	Urban	4.45	0.77
2496	Male	Youth	Urban	55.43	9.32
2497	Male	Youth	Urban	2.48	1.41
2498	Male	Youth	Urban	6.95	0.01
2499	Male	Youth	Urban	0.03	0.62
2500	Male	Youth	Urban	0.48	0.59
2501	Male	Youth	Urban	2.96	2.15
2502	Male	Youth	Urban	0.15	2.73
2503	Male	Youth	Urban	21.18	3.64
2504	Male	Youth	Urban	0.28	0.01
2505	Male	Youth	Urban	10.2	2.07
2506	Male	Youth	Urban	0	0
2507	Male	Youth	Urban	15.48	0.87

2508	Male	Youth	Urban	30.1	0.97
2509	Male	Youth	Urban	1.12	0.16
2510	Male	Youth	Urban	19.8	0.03
2511	Male	Youth	Urban	1.16	34.61
2512	Male	Youth	Urban	0	0
2513	Male	Youth	Urban	0.09	12.74
2514	Male	Youth	Urban	0.77	2.94
2515	Male	Youth	Urban	18.61	0.17
2516	Male	Youth	Urban	2.69	0.29
2517	Male	Youth	Urban	15.24	34.77
2518	Male	Youth	Urban	0	0
2519	Male	Youth	Urban	34.08	0.29
2520	Male	Youth	Urban	0.43	0.02
2521	Male	Youth	Urban	4.55	0.19
2522	Male	Youth	Urban	0.29	37.55
2523	Male	Youth	Urban	5.92	2.25
2524	Male	Youth	Urban	0	0
2525	Male	Youth	Urban	22.82	5.46
2526	Male	Youth	Urban	0.56	0.53
2527	Male	Youth	Urban	1.12	4.11
2528	Male	Youth	Urban	0.67	31.4
2529	Male	Youth	Urban	4.82	0.15
2530	Male	Youth	Urban	0	0
2531	Male	Youth	Urban	0	0
2532	Male	Youth	Urban	0.28	0.05
2533	Male	Youth	Urban	15.34	2.6
2534	Male	Youth	Urban	0.32	1.24
2535	Male	Youth	Urban	0.7	8.05
2536	Male	Youth	Urban	0	0
2537	Male	Youth	Urban	12.6	4.99
2538	Male	Youth	Urban	10.27	11.01
2539	Male	Youth	Urban	13.58	5.35
2540	Male	Youth	Urban	1.56	0.72
2541	Male	Youth	Urban	0.72	8.57
2542	Male	Youth	Urban	1.91	3.81
2543	Male	Youth	Urban	0	0
2544	Male	Youth	Urban	1.35	2.96
2545	Male	Youth	Urban	25.08	0.19
2546	Male	Youth	Urban	24.65	0.16
2547	Male	Youth	Urban	27.71	10.23
2548	Male	Youth	Urban	0.22	9.02
2549	Male	Youth	Urban	1.6	0.07
2550	Male	Youth	Urban	3.31	0.07
2551	Male	Youth	Urban	30.16	0.33
2552	Male	Youth	Urban	0.15	34.73
2553	Male	Youth	Urban	47.3	1.74
2554	Male	Youth	Urban	46.74	0.69
2555	Male	Youth	Urban	14.21	4.83
2556	Male	Youth	Urban	0.51	0.51
2557	Male	Youth	Urban	0.04	0.29
2558	Male	Youth	Urban	0.49	0.03
2559	Male	Youth	Urban	0.05	0.52
2560	Male	Youth	Urban	6.03	1.93
2561	Male	Youth	Urban	0.04	3.79
2562	Male	Youth	Urban	0.19	30.95
2563	Male	Youth	Urban	1.12	1.21
2564	Male	Youth	Urban	2.45	0.43

2565	Male	Youth	Urban	1.62	5.65
2566	Male	Youth	Urban	0.16	7.46
2567	Male	Youth	Urban	0.28	0.05
2568	Male	Youth	Urban	10.8	6.37
2569	Male	Youth	Urban	18.52	0.62
2570	Male	Youth	Urban	0.38	0.1
2571	Male	Youth	Urban	0	0
2572	Male	Youth	Urban	29.93	0.71
2573	Male	Youth	Urban	0.17	0.04
2574	Male	Youth	Urban	1.99	1.57
2575	Male	Youth	Urban	0	0
2576	Male	Youth	Urban	0.13	0.98
2577	Male	Youth	Urban	1.36	7.9
2578	Male	Youth	Urban	1.24	2.8
2579	Male	Youth	Urban	4.02	1.54
2580	Female	Middle	Urban	0.78	1.65
2581	Female	Middle	Urban	1.02	5.2
2582	Female	Middle	Urban	0.32	4.7
2583	Female	Middle	Urban	0.37	3.38
2584	Female	Middle	Urban	0	0
2585	Female	Middle	Urban	2.25	13.81
2586	Female	Middle	Urban	1.42	3.85
2587	Female	Middle	Urban	0.37	35.84
2588	Female	Middle	Urban	0.07	23.66
2589	Female	Middle	Urban	0	0
2590	Female	Middle	Urban	1.1	13.67
2591	Female	Middle	Urban	0.01	0.25
2592	Female	Middle	Urban	0.19	0.96
2593	Female	Middle	Urban	0.1	3.87
2594	Female	Middle	Urban	0.94	9.53
2595	Female	Middle	Urban	0.91	2.2
2596	Female	Middle	Urban	7.81	6.02
2597	Female	Middle	Urban	0	0
2598	Female	Middle	Urban	19.82	5.06
2599	Female	Middle	Urban	3.67	0.36
2600	Female	Middle	Urban	1.04	5.7
2601	Female	Middle	Urban	0.26	9.71
2602	Female	Middle	Urban	0.18	0.29
2603	Female	Middle	Urban	0.02	3.77
2604	Female	Middle	Urban	0.16	62.44
2605	Female	Middle	Urban	0.21	7.7
2606	Female	Middle	Urban	3.4	0.39
2607	Female	Middle	Urban	0	0
2608	Female	Toddler	Urban	2.18	1.14
2609	Female	Toddler	Urban	0.11	4.78
2610	Female	Old	Urban	0.44	0.26
2611	Female	Old	Urban	0.32	1.47
2612	Female	Old	Urban	0	0
2613	Female	Old	Urban	0.44	15.39
2614	Female	Old	Urban	0.32	1.42
2615	Female	Old	Urban	0	0
2616	Female	Old	Urban	0	0
2617	Female	Old	Urban	0.06	0.37
2618	Female	Old	Urban	0.63	6.25
2619	Female	Old	Urban	1.87	1.42
2620	Female	Old	Urban	0.64	5.58
2621	Female	Old	Urban	8.27	0.73

2622	Female	Old	Urban	1.1	21.42
2623	Female	Old	Urban	0	0
2624	Female	Youth	Urban	0.1	7.38
2625	Female	Youth	Urban	2	0.36
2626	Female	Youth	Urban	0.35	0.66
2627	Female	Youth	Urban	4.71	15.43
2628	Female	Youth	Urban	0.19	20.92
2629	Female	Youth	Urban	0.39	2.69
2630	Female	Youth	Urban	31.81	0.46
2631	Female	Youth	Urban	0.31	1.5
2632	Female	Youth	Urban	0.07	2.56
2633	Female	Youth	Urban	8.31	3.29
2634	Male	Middle	Urban	1.38	22.34
2635	Male	Middle	Urban	0.19	2.09
2636	Male	Middle	Urban	0.85	0.58
2637	Male	Middle	Urban	3.81	2.94
2638	Male	Middle	Urban	0.19	1.96
2639	Male	Middle	Urban	0.33	11.76
2640	Male	Middle	Urban	0.49	3.7
2641	Male	Middle	Urban	1.9	17.61
2642	Male	Middle	Urban	0.05	13.5
2643	Male	Middle	Urban	0.38	3.62
2644	Male	Middle	Urban	2.8	9.46
2645	Male	Middle	Urban	1.52	2.62
2646	Male	Middle	Urban	0.12	0.99
2647	Male	Middle	Urban	0.75	0.26
2648	Male	Middle	Urban	5.05	0.5
2649	Male	Middle	Urban	0	0
2650	Male	Middle	Urban	0.41	4.22
2651	Male	Middle	Urban	7.37	2.71
2652	Male	Middle	Urban	1.37	1.05
2653	Male	Middle	Urban	0.4	4.54
2654	Male	Middle	Urban	0	0
2655	Male	Middle	Urban	0.75	0.13
2656	Male	Middle	Urban	2.33	7.17
2657	Male	Middle	Urban	1.41	5.28
2658	Male	Middle	Urban	0.09	0.39
2659	Male	Middle	Urban	0.01	0.75
2660	Male	Middle	Urban	0.09	72
2661	Male	Middle	Urban	2.39	0.08
2662	Male	Middle	Urban	0	0
2663	Male	Toddler	Urban	0.03	0.13
2664	Male	Old	Urban	2.63	0.3
2665	Male	Old	Urban	0.43	0.94
2666	Male	Old	Urban	0	0
2667	Male	Old	Urban	1.22	22.42
2668	Male	Old	Urban	0.49	0.7
2669	Male	Old	Urban	0.31	18.05
2670	Male	Old	Urban	0	0
2671	Male	Old	Urban	0	0
2672	Male	Old	Urban	0.11	4.65
2673	Male	Old	Urban	0.85	0.05
2674	Male	Old	Urban	0.15	10.44
2675	Male	Old	Urban	1.63	34.73
2676	Male	Old	Urban	0.57	0.71
2677	Male	Old	Urban	0.07	2.68
2678	Male	Old	Urban	0.22	2.66

2679	Male	Old	Urban	0.91	0.98
2680	Male	Old	Urban	0.25	0.16
2681	Male	Old	Urban	0.15	0.78
2682	Male	Old	Urban	0	0
2683	Male	Old	Urban	0	0
2684	Male	Old	Urban	0.21	29.92
2685	Male	Old	Urban	0	0
2686	Male	Old	Urban	0	0
2687	Male	Old	Urban	2.6	0.27
2688	Male	Old	Urban	0	0
2689	Male	Old	Urban	0	0
2690	Male	Old	Urban	0.13	0.03
2691	Male	Old	Urban	1.05	0.03
2692	Male	Old	Urban	0.13	2.14
2693	Male	Old	Urban	2	1.42
2694	Male	Old	Urban	3.44	50.56
2695	Male	Old	Urban	0.84	1.59
2696	Male	Old	Urban	0.37	8.17
2697	Male	Old	Urban	0.19	1.97
2698	Male	Old	Urban	0	0
2699	Male	Old	Urban	2.47	27.3
2700	Male	Youth	Urban	0.6	0.4
2701	Male	Youth	Urban	0.73	4.14
2702	Male	Youth	Urban	0.94	0.01
2703	Male	Youth	Urban	0.06	1.32
2704	Male	Youth	Urban	0.14	0.57
2705	Male	Youth	Urban	0.67	31.4
2706	Male	Youth	Urban	0.21	1.36
2707	Male	Youth	Urban	0	0
2708	Male	Youth	Urban	0	0
2709	Male	Youth	Urban	4.45	14.07
2710	Male	Youth	Urban	15.21	1.28
2711	Male	Youth	Urban	0	0
2712	Male	Youth	Urban	0	0
2713	Male	Youth	Urban	0.85	3.12
2714	Male	Youth	Urban	9.2	14.67
2715	Male	Youth	Urban	0.23	1.61
2716	Male	Youth	Urban	0.04	6.81
2717	Male	Youth	Urban	0.67	0.47
2718	Female	Middle	Urban	0.41	0.66
2719	Female	Middle	Urban	1.41	0.18
2720	Female	Middle	Urban	9.34	4.49
2721	Female	Middle	Urban	0	0
2722	Female	Middle	Urban	6.38	7.15
2723	Female	Middle	Urban	0	0
2724	Female	Middle	Urban	0.68	0.45
2725	Female	Middle	Urban	0	0
2726	Female	Toddler	Urban	28.59	0.37
2727	Female	Toddler	Urban	58.37	0.34
2728	Female	Old	Urban	21.61	1.31
2729	Female	Old	Urban	7.75	0.37
2730	Female	Old	Urban	12.26	11.31
2731	Female	Youth	Urban	10.92	2.47
2732	Female	Youth	Urban	3	0.26
2733	Female	Youth	Urban	15.05	0.18
2734	Female	Youth	Urban	0	0
2735	Male	Middle	Urban	0.59	0.68

2736	Male	Middle	Urban	1.66	2.23
2737	Male	Middle	Urban	29.52	2.17
2738	Male	Toddler	Urban	41.79	0.01
2739	Male	Old	Urban	0	0
2740	Male	Old	Urban	16.05	0.23
2741	Male	Old	Urban	27.86	1.78
2742	Male	Old	Urban	0	0
2743	Male	Old	Urban	0	0
2744	Male	Old	Urban	0	0
2745	Male	Old	Urban	0	0
2746	Male	Youth	Urban	5.04	0.58
2747	Male	Youth	Urban	9.56	1.45
2748	Female	Middle	Urban	2.98	5.26
2749	Female	Middle	Urban	2.94	5.93
2750	Female	Middle	Urban	9.41	2.56
2751	Female	Middle	Urban	1.01	32.23
2752	Female	Middle	Urban	17.52	10.23
2753	Female	Middle	Urban	0	0
2754	Female	Middle	Urban	35.74	0.08
2755	Female	Middle	Urban	33.66	11.27
2756	Female	Middle	Urban	33.54	0.06
2757	Female	Middle	Urban	0.43	1.21
2758	Female	Middle	Urban	14.01	1.55
2759	Female	Middle	Urban	1.06	0.04
2760	Female	Middle	Urban	2.34	0.62
2761	Female	Middle	Urban	0.81	1.01
2762	Female	Middle	Urban	27.04	0.78
2763	Female	Middle	Urban	63.99	0.83
2764	Female	Middle	Urban	4	0.08
2765	Female	Middle	Urban	67.97	1.73
2766	Female	Middle	Urban	4.15	0.19
2767	Female	Middle	Urban	1.33	0.17
2768	Female	Middle	Urban	36.23	1.54
2769	Female	Middle	Urban	1.61	0.25
2770	Female	Middle	Urban	30.19	0.86
2771	Female	Toddler	Urban	2.25	0.88
2772	Female	Toddler	Urban	1.79	0.02
2773	Female	Toddler	Urban	3.96	0.02
2774	Female	Toddler	Urban	40.96	1.37
2775	Female	Old	Urban	0.9	1.39
2776	Female	Old	Urban	1.98	0.13
2777	Female	Old	Urban	0	0
2778	Female	Old	Urban	5.17	12.5
2779	Female	Old	Urban	1.82	3.66
2780	Female	Old	Urban	2.71	9.17
2781	Female	Youth	Urban	0.4	0.26
2782	Female	Youth	Urban	39.69	6.26
2783	Female	Youth	Urban	48.53	0.96
2784	Female	Youth	Urban	0	0
2785	Female	Youth	Urban	63.04	0.61
2786	Female	Youth	Urban	15.31	34.96
2787	Female	Youth	Urban	0	0
2788	Female	Youth	Urban	72.87	1.58
2789	Female	Youth	Urban	16.07	12.87
2790	Female	Youth	Urban	8.18	9.85
2791	Female	Youth	Urban	2.17	27.92
2792	Female	Youth	Urban	6.72	3.2

2793	Female	Youth	Urban	2.56	10.88
2794	Female	Youth	Urban	1.91	7.16
2795	Female	Youth	Urban	0.3	7.29
2796	Female	Youth	Urban	31.64	0.49
2797	Female	Youth	Urban	0	0
2798	Female	Youth	Urban	0	0
2799	Female	Youth	Urban	46.29	7.08
2800	Female	Youth	Urban	7.95	0.57
2801	Female	Youth	Urban	19.43	1.7
2802	Female	Youth	Urban	7.3	3.5
2803	Female	Youth	Urban	0	0
2804	Female	Youth	Urban	1.95	0.03
2805	Male	Middle	Urban	6.08	0.91
2806	Male	Middle	Urban	0	0
2807	Male	Middle	Urban	2.83	20.69
2808	Male	Middle	Urban	4.41	0.05
2809	Male	Middle	Urban	5.5	1.33
2810	Male	Middle	Urban	9.21	0.11
2811	Male	Middle	Urban	5.4	2.78
2812	Male	Middle	Urban	5.29	31.37
2813	Male	Middle	Urban	2.08	0.67
2814	Male	Middle	Urban	0	0
2815	Male	Middle	Urban	5.94	2.86
2816	Male	Middle	Urban	71.66	7.9
2817	Male	Middle	Urban	6.52	0.64
2818	Male	Middle	Urban	0	0
2819	Male	Middle	Urban	0	0
2820	Male	Middle	Urban	6.66	0.1
2821	Male	Middle	Urban	16.13	2.12
2822	Male	Middle	Urban	6.58	1.11
2823	Male	Middle	Urban	1.14	0.55
2824	Male	Middle	Urban	0	0
2825	Male	Middle	Urban	17.9	10.55
2826	Male	Middle	Urban	0	0
2827	Male	Middle	Urban	0	0
2828	Male	Middle	Urban	3.27	0.5
2829	Male	Middle	Urban	28.29	16.89
2830	Male	Middle	Urban	2.72	0.14
2831	Male	Middle	Urban	4.46	3.27
2832	Male	Middle	Urban	3.05	0.23
2833	Male	Middle	Urban	1.38	1.26
2834	Male	Middle	Urban	11.6	11.06
2835	Male	Middle	Urban	1.17	0.67
2836	Male	Middle	Urban	5.09	0.36
2837	Male	Middle	Urban	0.52	5.25
2838	Male	Middle	Urban	12.95	0.59
2839	Male	Middle	Urban	17.89	0.93
2840	Male	Middle	Urban	2.14	0.07
2841	Male	Middle	Urban	1.22	1.63
2842	Male	Middle	Urban	1.39	0.11
2843	Male	Toddler	Urban	5.06	3.34
2844	Male	Toddler	Urban	58.37	0.27
2845	Male	Toddler	Urban	0	0
2846	Male	Toddler	Urban	4.78	0.5
2847	Male	Toddler	Urban	7.09	5.04
2848	Male	Toddler	Urban	5.29	2.96
2849	Male	Old	Urban	0.49	16.18

2850	Male	Old	Urban	3.29	0.09
2851	Male	Old	Urban	2.08	8.03
2852	Male	Old	Urban	0	0
2853	Male	Old	Urban	0.19	0.4
2854	Male	Old	Urban	0	0
2855	Male	Old	Urban	76.85	1.96
2856	Male	Old	Urban	0.4	20.07
2857	Male	Old	Urban	2.77	12.05
2858	Male	Old	Urban	2.71	1.27
2859	Male	Old	Urban	9.27	11.37
2860	Male	Old	Urban	0	0
2861	Male	Old	Urban	15.89	1.65
2862	Male	Old	Urban	11.14	3.92
2863	Male	Youth	Urban	34.14	3.14
2864	Male	Youth	Urban	24.13	0.19
2865	Male	Youth	Urban	10.2	0.11
2866	Male	Youth	Urban	5.38	0.68
2867	Male	Youth	Urban	0.66	0.25
2868	Male	Youth	Urban	0	0
2869	Male	Youth	Urban	0	0
2870	Male	Youth	Urban	14.39	1.99
2871	Male	Youth	Urban	68.05	0.22
2872	Male	Youth	Urban	0	0
2873	Male	Youth	Urban	0	0
2874	Male	Youth	Urban	0	0
2875	Male	Youth	Urban	9.29	5.69
2876	Male	Youth	Urban	0	0
2877	Male	Youth	Urban	1.64	34.06
2878	Male	Youth	Urban	1.84	1.27
2879	Male	Youth	Urban	32.97	7.19
2880	Male	Youth	Urban	0.13	2.75
2881	Male	Youth	Urban	3.12	67.36
2882	Male	Youth	Urban	0	0
2883	Male	Youth	Urban	33.04	2.92
2884	Male	Youth	Urban	14.58	3.61
2885	Male	Youth	Urban	1.08	3.7
2886	Male	Youth	Urban	5.26	2.06
